# Discovery of Flavone Derivatives Containing Carboxamide Fragments as Novel Antiviral Agents

**DOI:** 10.3390/molecules28052179

**Published:** 2023-02-26

**Authors:** Bobo Zhao, Jiali Wang, Lu Wang, Ziwen Wang, Aidang Lu

**Affiliations:** 1 School of Chemical Engineering and Technology, Hebei University of Technology, Tianjin 300401, China; 2 Tianjin Key Laboratory of Structure and Performance for Functional Molecules, College of Chemistry, Tianjin Normal University, Tianjin 300387, China

**Keywords:** natural product, flavone derivatives, carboxamide fragment, anti-TMV activity, mode of action

## Abstract

Plant virus diseases seriously affect the yield and quality of agricultural products, and their prevention and control are difficult. It is urgent to develop new and efficient antiviral agents. In this work, a series of flavone derivatives containing carboxamide fragments were designed, synthesized, and systematically evaluated for their antiviral activities against tobacco mosaic virus (TMV) on the basis of a structural–diversity–derivation strategy. All the target compounds were characterized by ^1^H-NMR, ^13^C-NMR, and HRMS techniques. Most of these derivatives displayed excellent in vivo antiviral activities against TMV, especially **4m** (inactivation inhibitory effect, 58%; curative inhibitory effect, 57%; and protection inhibitory effect, 59%), which displayed similar activity to ningnanmycin (inactivation inhibitory effect, 61%; curative inhibitory effect, 57%; and protection inhibitory effect, 58%) at 500 μg mL^−1^; thus, it emerged as a new lead compound for antiviral research against TMV. Antiviral mechanism research by molecular docking demonstrated that compounds **4m**, **5a**, and **6b** could interact with TMV CP and disturb virus assembly.

## 1. Introduction

Plant diseases caused by fungal and viral pathogens are extremely destructive to crops, seriously affecting the growth and maturity of crops, causing huge economic losses and triggering a food crisis [1,2,3]. Tobacco mosaic virus (TMV), known as “plant cancer”, is one of the earliest and most extensively researched model viruses [4]. It has a very wide host range and can infect more than 800 kinds of plants in 65 families, including tobacco, pepper, tomato, eggplant, etc. [5]. Once infected, the virus will transfer from the infected cells to the adjacent healthy cells, step-by-step destroying the host’s defense system. At present, it is still difficult to prevent and treat it [6,7,8]. Ribavirin is widely used as a plant virus inhibitor to prevent plant virus diseases, but its inhibitory effect is less than 50% at 500 μg/mL. To date, no effective agrochemicals can absolutely restrain TMV once it has infected plants [9,10]. Therefore, it is of great significance to develop new antiviral agents. Natural products (NPs) have a long history as a source of compounds and inspiration for pharmaceuticals and crop protection compounds [11,12,13,14,15,16]. However, NPs often lack the necessary physicochemical properties, efficacy, and acceptable biological and environmental profiles that constrain their suitability for use in agricultural settings, so only a few NPs can be directly used [17]. Nevertheless, NPs have had their greatest impact as inspiration or models for crop protection products to develop agrochemicals [17]. For example, Dufulin is a biological antiviral agent inspired by an α-aminophosphonic derivative in sheep [18,19].

**Figure 1:**
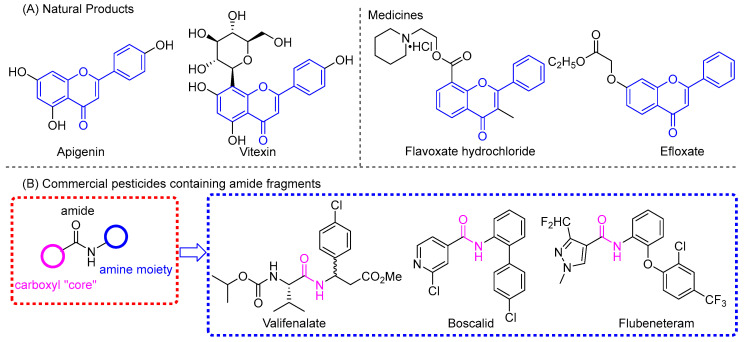
Chemical structures of flavonoids (**A**) and carboxamide fragments (**B**) with biological activities.

Flavonoids are plant secondary metabolites with a C_6_-C_3_-C_6_ skeleton, which widely exist in plants and play an important role in plant breeding and metabolism [20]. According to their chemical structures, flavonoids can be divided into chalcones, flavanones, flavanonols, flavones, flavonols, isoflavones, etc. [21]. Flavonoids have many pharmacological effects including antioxidation, anti-inflammatory, anti-carcinogenic, anti-viral, and fungicidal effects [22,23,24,25,26,27], and have always been used as lead compounds in the development of medicines and pesticides. For instance, the flavone skeleton has been embedded in the marketed medicines flavoxate hydrochloride and efloxate (Figure 1). The anti-TMV activities of vitexin (Figure 1), quercetin, fistulaflavonoids, and flavonoid glycosides have been confirmed [28,29,30]. Chen et al. isolated some isoflavones from tobacco roots and stems, and applied them to control plant viruses. They found that some of the isoflavones had better antiviral properties than that of commercial agents [31]. Two new flavones, 8-hydroxy-7-(3-hydroxypropyl)-2′-methoxyflavone and 8-hydroxy-7-(2-hydroxyethyl)-2′-methoxyflavone, were isolated from the whole plants of *Cassia pumila* and found to have potential anti-TMV activities with inhibition rates of 30.2% and 28.2%, respectively [32,33]. Encouraged by these results, a series of oxazinyl flavonoids were synthesized and evaluated against TMV in our previous work [34]. The carboxamide group plays an important role in commercialized registered pesticides. There is no “absent” amide bond in the chemical structure of commercial succinate dehydrogenase inhibitor (SDHIs) fungicides, so they are also called carboxamide fungicides, such as the marketed fungicides valifenalate, boscalid, and flubeneteram, etc. (Figure 1). Among these compounds, amide bonds act as an important bridge connecting the carboxylic acid parts and the amine moiety. The hydrophobic tail is mostly consisted of aromatic amines of five or six members, while the structural types of polar moiety are relatively diverse, such as pyrazole, pyridine, pyrazine, benzene ring, etc. [35,36] Many methods for obtaining amide functions have been improved or developed, which makes the synthesis of these compounds more convenient and greener [37,38].

**Figure 2:**
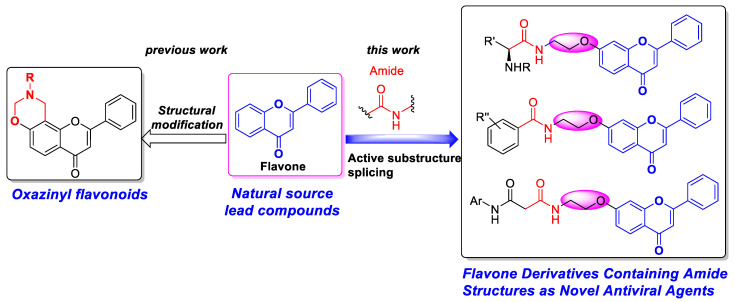
Design of flavone derivatives containing amide fragments.

In our previous work, we synthesized a series of oxazinyl flavonoids (Figure 2) [34], which displayed higher anti-TMV activities than that of the natural product apigenin (Figure 1). The anti-TMV activity of 7-(benzyloxy)-2-phenyl-4*H*-chromen-4-one (**2c**) synthesized in our previous work was significantly higher than that of 7-hydroxy flavone (**1**) (Table 1). It was found that substituents of hydroxyl oxygen atom had a significant effect on the activity. In this paper, a series of flavone derivatives containing the carboxamide fragments were designed and synthesized based on structural diversity derivation with flavonoid as the mother nucleus structure (Figure 2), and their anti-TMV activities were systematically investigated to find new high-activity compounds for plant protection. The structures of all target compounds were confirmed by ^1^H NMR, ^13^C NMR, and HRMS. Using 7-hydroxy flavone as the lead compound, carboxylic acid parts were introduced on the position of 7-OH through a bridge of the amide bond. Further antiviral mechanism research exhibited that flavone derivatives containing an amide fragment could interact with TMV CP (coat protein) and inhibit virus assembly.

## 2. Results

### 2.1. Chemistry

**Scheme 1:**
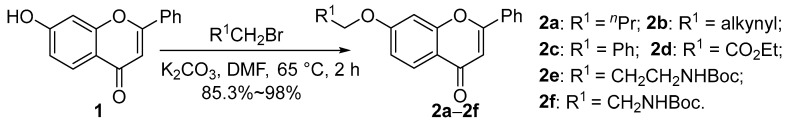
Synthesis of **2a**–**2f**.

7-Hydroxyacetophenone was synthesized according to the method in the literature [34,39]. Compounds **2a**–**2f** were synthesized from 7-hydroxyflavone by reacting with different substituted halogenated hydrocarbons in high yields (Scheme 1).

**Scheme 2:**
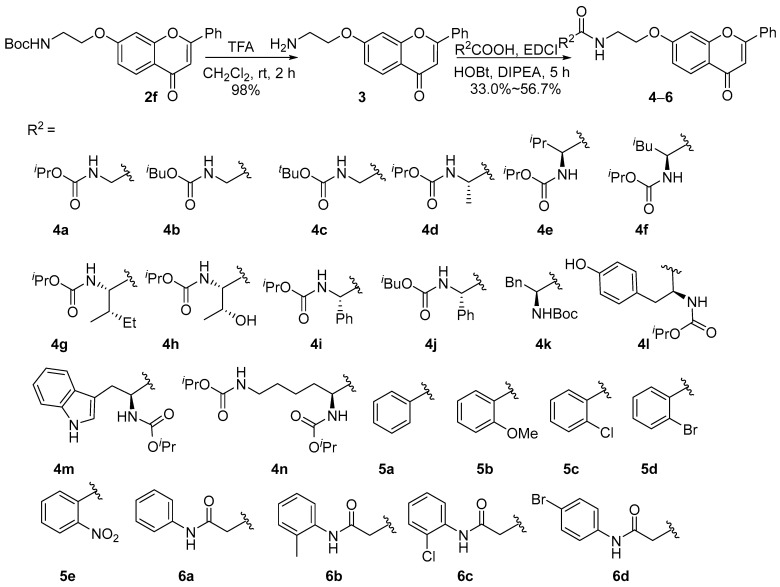
Synthesis of **4a**–**4n**, **5a**–**5e**, and **6a**–**6d**.

With compound **2f** in hand, deprotection of the *tert*-butyl carbonyl group of **2f** with trifluoroacetic acid resulted in amine **3**. Compounds **4**–**6** were successfully obtained by amidation reaction in the presence of 1-ethyl-3-(3-dimethylaminopropyl)carbodiimide hydrochloride (EDCI) and 1-hydroxybenzotriazole (HOBt) (Scheme 2).

### 2.2. Phytotoxic Activity

According to the phytotoxic activity tests, the target compounds were found to be harmless to plants at a concentration of 500 μg/mL. At this concentration, the leaves of tobacco were not found to be rotten or spotted, which could grow healthily and normally. Detailed testing procedures can be seen in our previous report [5,7] and can be found in the Supplementary Materials.

### 2.3. Antiviral Activity

#### In Vivo Anti-TMV Activity

The anti-TMV activities of designed compounds are shown in Table 1 with commercial ribavirin and ningnanmycin as the control drugs. According to the results in Table 1, compounds **2a**–**2f**, **4a**–**4n**, **5a**–**5e**, and **6a**–**6d** exhibited moderate to excellent anti-TMV activities, except for **4e** and **4f**. Almost all the target compounds showed higher anti-TMV activities than ribavirin. Compound **4m** exhibited the highest anti-TMV activity at 500 μg/mL (inactivation activity, 58%; curative activity, 57%; protection activity, 59%), which was significantly higher than that of ribavirin (inactivation activity, 39%; curative activity, 40%; protection activity, 39%) and similar to that of ningnamycin (inactivation activity, 61%; curative activity, 57%; protection activity, 58%).

### 2.4. Mode of Action Studies

#### Docking Studies

To further study the mechanism of the interaction between flavone derivatives and TMV CP, we use AutoDock Vina 1.1.2 for molecular docking [40]. The docking poses are ranked according to their docking sites, and the lowest binding energy of the macromolecule–ligand complex is considered the best. It can be proven that there are some H-bond interactions and strong binding affinity between flavone derivatives containing amide fragments and TMV CP.

## 3. Discussion

### 3.1. Synthesis

According to the reported method [39,41], the 7-position hydroxyl group of 7-hydroxyacetophenone was smoothly reacted with bromo compounds in basic K_2_CO_3_ to gain the compounds **2a**–**2f** (Scheme 1). Starting from intermediate **2f**, compound **3** can be successfully obtained by treating it with trifluoroacetic acid. The addition of isopropyl chloroformate, isobutyl chloroformate, or di-tert-butyl dicarbonate to a solution of amino acids in aqueous sodium hydroxide produced amino acid derivatives in good yield [42]. Compounds **4**–**6** can be successfully gained by the amidation reaction of compound **3** with amino acid derivatives [43], benzoic acid and its derivatives, and malonic acid derivatives [44] in the presence of 1-ethyl-3-(3-dimethylaminopropyl)carbodiimide hydrochloride (EDCI) and 1-hydroxybenzotriazole (HOBt) (Scheme 2). We mainly introduced structural diversity functional groups into C-7 of 7-hydroxyflavone by the active structures splicing strategy. Amino acids, aromatic carboxylic acids, and malonic acid derivatives fragments were introduced into the flavonoid skeleton to achieve structural diversity derivation of flavonoid compounds.

The structures of obtained compounds were confirmed by ^1^H, ^13^C NMR spectroscopy, and HRMS analysis. 7-Hydroxyflavone (**1**) was obtained by our previously reported method [34], which is consistent with the literature report [41]. Compound **4a** was used as an example for chemical structure characterization analysis. The spectrum of ^1^H NMR (Figure S15) exhibited one methyl signal at *δ*_H_ 1.16, three signals of methylene at *δ*_H_ 3.51, *δ*_H_ 3.60, and *δ*_H_ 4.17, one signal of oxygenated methine at *δ*_H_ 4.63–4.83. Moreover, as described in Figure S16, the data of ^13^C NMR for **4a** indicated 19 signals of carbon, including five saturated carbons (*δ*_C_ 67.7, 67.5, 43.9, 38.5, and 22.5). These spectroscopic features confirmed that the amino acid fragment was successfully introduced into 7-hydroxyflavone.

### 3.2. Structure–Activity Relationship of the Antiviral Activity

The antiviral activities of compounds **2a**–**2f**, **4a**–**4n**, **5a**–**5e**, and **6a**–**6d** were listed in Table 1, with commercial plant virucides ningnanmycin and ribavirin as controls. Most of these compounds showed higher antiviral activities than ribavirin and were obviously superior to compound **1**. As shown in Table 1, compounds **2a**–**2f** with alkyl substitutions on the 7-position of the O atom were higher than that of ribavirin. In contrast, the anti-TMV activity of the substituent containing the ester group (**2d**), the Boc (*t*-butyloxycarbonyl) protected amino group (**2e** and **2f**), and the amino group (**3**) were obviously better than that of compounds containing the simple alkyl substituent (**2a**, **2b**, and **2c**).

Amino acids play important roles both as building blocks of peptides and proteins and as important constituents of biologically active compounds, such as benthiavalicarb, iprovalicarb, and valiphenal [45]. As early as 1951, Li et al. reported that some amino acids had certain effects on the reproduction of TMV [46]. Amino acid gossypol Schiff bases were evaluated for their anti-TMV activities by Zhang et al. [47]. To systematically investigate the effect of amino acid fragments with different structures on the anti-TMV activity, 10 kinds of cheap and commercial amino acids, including simple glycine and *L*-tryptophan containing indole, were used as starting materials to react with different chloroformates to obtain corresponding carboxylic acids and the target compounds **4a**–**4n**. In general, compounds **4a**–**4n** containing amino acid fragments at the 7-position of the O atom displayed higher levels of anti-TMV activity than compounds with smaller groups at the same position. Among compounds **4a**, **4d**–**4e**, **4f**–**4g**, **4i**, **4l**, and **4n**, protected by the isopropoxycarbonyl group, glycine derivative **4a** had the best antiviral activity. *L*-valine derivatives (**4e**), *L*-leucine derivatives (**4f**), *L*-isoleucine derivatives (**4g**), *L*-2-phenylglycine derivatives (**4i**), and *L*-2-(4-OH-benzyl)glycine derivatives (**4l**) were not conducive to anti-TMV activity (**4a** > **4n** > **4d** > **4i** > **4e** > **4g** ≈ **4l** > **4e** ≈ **4f**). In particular, the inhibition rate of compounds **4e** and **4f** are lower than 40% (**4e**: inactivation inhibitory effect, 39%; curative inhibitory effect, 37%; and protection inhibitory effect, 39%; **4f**: inactivation inhibitory effect, 38%; curative inhibitory effect, 36%; and protection inhibitory effect, 36%) at 500 μg mL^−1^. However, compound **4m**, starting from (isopropoxycarbonyl)-*L*-tryptophan, showed excellent anti-TMV activity (inactivation inhibitory effect, 58%; curative inhibitory effect, 57%; and protection inhibitory effect, 59%), which is similar to that of ningnanmycin (inactivation inhibitory effect, 61%; curative inhibitory effect, 57%; and protection inhibitory effect, 58%) at 500 μg mL^−1^. The different protective groups of amino acids also had certain effects on antiviral activity. Compared with compounds **4a**, **4b**, and **4c**, when the amino protecting group was isopropoxycarbonyl, it was superior to *tert*-butyloxycarbonyl and *iso*-butyloxycarbonyl. This rule was consistent with the anti-TMV activity of compounds **4i** and **4j**.

Compounds **5a**–**5e**, prepared from benzoic acid and its derivatives with compound **3**, have higher anti-TMV activity than ribavirin, but the antiviral activity was greatly reduced when the ortho position of the benzene ring of carboxyamide group contained methoxy (**5b**: inactivation inhibitory effect, 45%; curative inhibitory effect, 44%; and protection inhibitory effect, 46%) at 500 μg mL^−1^. Compounds **6a**–**6e**, synthesized from 3-oxo-3-(phenylamino)propanoic acid, exhibited higher anti-TMV activity than that of ribavirin. Different substituents on the benzene ring of the amino group were beneficial to the improvement of activity and could increase the anti-TMV activity. In particular, compound **6b** (inactivation activity, 54%; curative activity, 57%; protection activity, 54%) exhibited significantly higher activity than ribavirin (inactivation activity, 39%; curative activity, 40%; protection activity, 39%) at 500 μg/mL.

Summarizing the structure–activity relationship, it was found that carboxylic acid parts introduced on the position of 7-OH through a bridge of the amide bond could improve the anti-TMV activity.

### 3.3. Study on the Mechanism of Anti-TMV Activity

#### Molecular Docking Study

**Figure 3:**
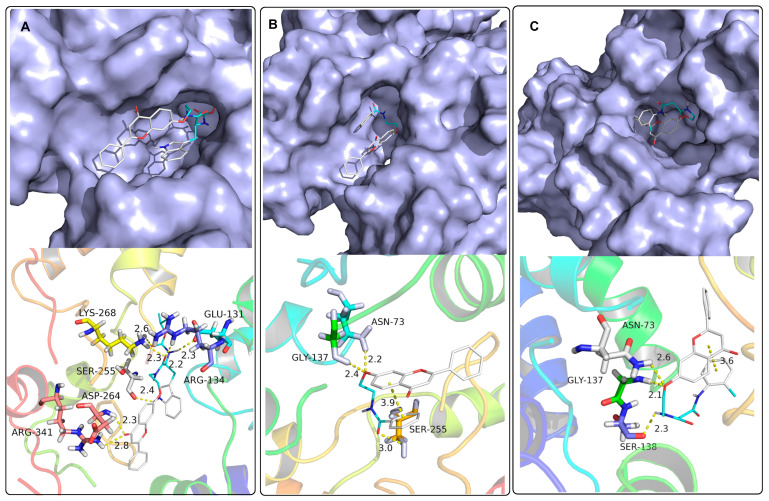
Molecule docking results of **4m** (**A**), **5a** (**B**), and **6b** (**C**) with TMV CP.

To further investigate and evaluate the detailed intermolecular interactions between flavone derivatives and potential targets, molecular docking was performed. The detailed calculation procedures for molecular docking research were carried out according to the literature and are described in the Supplementary Materials [48]. Compounds **4m**, **5a**, and **6b** were selected for molecular docking with TMV CP (PDB code 1EI7) [49]. For comparison, compounds **1** and **2a** were also chosen for molecular docking with TMV CP, and the results were in the Supplementary Materials (Figure S61). As depicted in Figure 3, compounds **4m**, **5a**, and **6b** folded similar to a hairpin and entered into the hole-shaped active binding pocket created by TMV CP (Figure 3). The results showed that compound **4m** was laid into the TMV CP active pocket and formed seven conventional hydrogen bonds with the active sites of ARG 134 (2.8 Å and 2.2 Å), ASP 264 (2.3 Å), SER 255 (2.4 Å), LYS 268 (2.6 Å and 2.3 Å), GLU 131 (2.3 Å) (Figure 3A). Due to the folding of compound **4m**, the carbonyl groups and amino groups were fully exposed, which made it easier to interact with amino acid residues. As shown in Figure 3A, not only the flavonoid structure and carboxamide group can interact with TMV CP, but also the indole has a hydrogen bond with TMV CP. The active O atom at position 7 of compounds **5a** and **6b** can interact with free amine N-H of the adjacent two amino acids ASN 73 and GLY137 to form hydrogen bonds (Figure 3B,C). Compound **5a** can also provide an O atom of the carboxyl group to form a hydrogen bond with SER 255, while compound **6b** bonds with SER 138 as seen in Figure 3B,C. In particular, the two aromatic rings of compounds **5a** and **6b** were almost parallel, forming the additional pi–pi interactions effect. Compared with Figure S61A, although compound **1** will also be embedded in the same cavity, its interaction mode is obviously different, and the benzene ring of the flavonoid structure was at the innermost end. Compound **1** provided an O atom to form two hydrogen bonds with LYS 268 and ARG 134, and supplied -OH to form a hydrogen bond with ARG 134. As shown in Figure S61B, after the alkylation of the hydroxyl group, the method of flavonoid embedding into the TMV CP cavity had changed significantly. Compound **2a** was laid into the TMV CP active pocket and formed two conventional hydrogen bonds with the active sites of GLN 257 (2.7 Å) and ASN 73 (2.4 Å).

The binding free energies of compounds **4m**, **5a**, and **6b** to TMV CP were −8.6 kcal/mol, −8.1 kcal/mol, and −8.4 kcal/mol, respectively. In contrast, the binding free energies of compounds **1** and **2a** to TMV CP were only −7.3 kcal/mol and −7.5 kcal/mol, respectively. The lower the binding free energy, the higher the affinity between the receptor and the ligand [50]. The results of molecular docking showed that the binding energy of amino acid, aromatic formic acid, and malonic acid derivatives introduced at the oxygen atom through the amide bond were lower than that of oxazinyl flavonoids [34], which were more conducive to anti-TMV activity.

The results of molecular docking showed that the introduction of ethyl made the molecular structure more flexible, and it could be embedded into the cavity of TMV CP as stably as a hairpin. Through the bridging of the amide bond, it can not only provide the interaction sites with TMV CP, but also facilitate the introduction of other active groups. Compounds **4**–**6** bearing different groups could interact with TMV CP to disturb the assembly of TMV virus particles, thus showing good anti-TMV activity. They were also consistent with the activity test.

## 4. Materials and Methods

### 4.1. Synthetic Procedures

#### 4.1.1. Reagents and Instruments

All reagents used were analytical reagent (AR) grade or chemically pure (CR), which were purchased from commercial sources (Tianjin Guangda Chemical Reagents Ltd., Tianjin, China). The melting point of the target compounds were measured on an X-4 binocular microscope (Beijing Zhongke Instrument Co., Ltd., Beijing, China). NMR spectra were acquired with a 400 MHz (100 MHz for ^13^C) instrument (Bruker, Billerica, MA, USA) at room temperature. Chemical shifts were measured relative to residual solvent peaks of CDCl_3_ (^1^H: *δ* = 7.26 ppm; ^13^C: *δ* = 77.0 ppm) and DMSO-*d*_6_ (^1^H: *δ* = 2.5 and 3.3 ppm; ^13^C: *δ* = 39.9 ppm) as internal standards. The following abbreviations are used to designate chemical shift multiplicities: s = singlet, d = doublet, dd = doublet of doublets, t = triplet, m = multiplet, and brs = broad singlet. HRMS data were recorded with a QFT-ESI instrument (Varian, Palo Alto, CA, USA).

#### 4.1.2. Synthesis of Compounds **2a**–**2f**

Synthesis of compounds **2a**–**2e**. 7-Hydroxyflavone (0.119 g, 0.5 mmol) and anhydrous K_2_CO_3_ (0.414 g, 3 mmol) were added into *N*,*N*-dimethylformamide (DMF) (3 mL), then the mixture was stirred at room temperature for 30 min. The mixture continued to stir and control the temperature at 70 °C for 2 h after adding halohydrocarbon compounds (1.5 mmol). After 7-hydroxyflavone was completely consumed (monitored by Thin Layer chromatography, TLC), the mixture was diluted with water (10 mL) and washed with ethyl acetate (3 × 15 mL). The combined organic phase was dried with anhydrous sodium sulfate. The crude product was obtained after removing the solvent in vacuo and purified by column chromatography.

7-Butoxy-2-phenyl-4*H*-chromen-4-one (**2a**). Light yellow solid, 92.5% yield, m.p. 82–85 °C; ^1^H NMR (400 MHz, CDCl_3_) *δ* 8.13 (d, *J* = 8.7 Hz, 1H), 8.01–7.83 (m, 2H), 7.59–7.45 (m, 3H), 6.98 (d, *J* = 11.1 Hz, 2H), 6.77 (s, 1H), 4.10 (s, 2H), 1.84 (d, *J* = 6.5 Hz, 2H), 1.53 (h, *J* = 7.3 Hz, 2H), 1.01 (t, *J* = 7.4 Hz, 3H); ^13^C NMR (100 MHz, CDCl_3_) *δ* 177.9, 163.8, 163.0, 158.0, 131.9, 131.4, 129.0, 127.0, 126.2, 117.7, 114.8, 107.5, 100.9, 68.5, 31.0, 19.2, 13.8; HR-MS (ESI): calcd for C_19_H_18_O_3_ [M + H]^+^ 295.1329, found (ESI^+^) 295.1336.
2-Phenyl-7-(prop-2-yn-1-yloxy)-4*H*-chromen-4-one (**2b**). Light brown solid, 95.0% yield, m.p. 164–169 °C; ^1^H NMR (400 MHz, CDCl_3_) *δ* 8.20 (d, *J* = 8.8 Hz, 1H), 7.97–7.91 (m, 2H), 7.59–7.52 (m, 3H), 7.14–7.05 (m, 2H), 6.81 (s, 1H), 4.86 (s, 2H), 2.65 (s, 1H); ^13^C NMR (100 MHz, CDCl_3_) *δ* 177.8, 163.2, 161.9, 157.7, 131.8, 131.5, 129.0, 127.2, 126.2, 118.5, 114.7, 107.6, 101.8, 77.4, 76.6, 56.2; HR-MS (ESI): calcd for C_18_H_12_O_3_ [M + H]^+^ 277.0859, found (ESI^+^) 277.0862.
7-(Benzyloxy)-2-phenyl-4*H*-chromen-4-one (**2c**). White solid, 95% yield, m.p. 173–180 °C; ^1^H NMR (400 MHz, CDCl_3_) *δ* 8.16 (d, *J* = 8.6 Hz, 1H), 7.96–7.88 (m, 2H), 7.52 (d, *J* = 5.7 Hz, 3H), 7.43–7.48 (m, 5H), 7.07 (d, *J* = 9.7 Hz, 2H), 6.78 (s, 1H), 5.20 (s, 2H); ^13^C NMR (100 MHz, CDCl_3_) *δ* 177.9, 163.4, 163.2, 158.0, 135.8, 131.9, 131.5, 129.1, 128.8, 128.5, 127.6, 127.2, 126.3, 118.0, 115.0, 107.6, 101.6, 70.6; HR-MS (ESI): calcd for C_22_H_17_O_3_ [M + H]^+^ 329.1172, found (ESI^+^) 329.1176.
Ethyl 2-((4-oxo-2-phenyl-4*H*-chromen-7-yl)oxy)acetate (**2d**). Light brown solid, 85.3% yield, m.p. 89–93 °C; ^1^H NMR (400 MHz, CDCl_3_) *δ* 8.16 (d, *J* = 8.8 Hz, 1H), 7.97–7.71 (m, 2H), 7.52 (d, *J* = 5.5 Hz, 3H), 7.08–6.98 (m, 1H), 6.99–6.89 (m, 1H), 6.77 (s, 1H), 4.75 (s, 2H), 4.31 (q, *J* = 7.1 Hz, 2H), 1.32 (t, *J* = 7.1 Hz, 3H); ^13^C NMR (100 MHz, CDCl_3_) *δ* 177.8, 168.0, 163.3, 162.2, 157.8, 131.8, 131.6, 129.1, 127.5, 126.2, 118.7, 114.3, 107.7, 101.7, 65.1, 61.8, 14.2; HR-MS (ESI): calcd for C_19_H_16_O_5_ [M + H]^+^ 325.1071, found (ESI^+^) 325.1069.
Tert-butyl(3-((4-oxo-2-phenyl-4*H*-chromen-7-yl)oxy)propyl)carbamate (**2e**). White solid, 90.2% yield, m.p. 144–150 °C; ^1^H NMR (400 MHz, CDCl_3_) *δ* 8.13 (d, *J* = 9.0 Hz, 1H), 7.92 (d, *J* = 6.4 Hz, 2H), 7.53 (s, 3H), 6.99 (d, *J* = 7.7 Hz, 2H), 6.86 (s, 1H), 4.15 (t, *J* = 5.6 Hz, 2H), 3.36 (s, 2H), 2.11–2.01 (m, 2H), 1.45 (s, 9H); ^13^C NMR (100 MHz, CDCl_3_) *δ* 177.9, 163.4, 163.0, 157.9, 156.1, 131.8, 131.4, 129.0, 127.0, 126.2, 117.8, 114.7, 107.5, 100.9, 79.3, 66.4, 37.7, 29.5, 28.4; HR-MS (ESI): calcd for C_23_H_25_NO_5_ [M + H]^+^ 396.1806, found (ESI^+^) 396.1802.
Tert-butyl (2-((4-oxo-2-phenyl-4*H*-chromen-7-yl)oxy)ethyl)carbamate (**2f**). White solid, 98.0% yield, m.p. 129–132 °C; ^1^H NMR (400 MHz, CDCl_3_) *δ* 8.13 (d, *J* = 8.4 Hz, 1H), 7.93–7.87 (m, 2H), 7.52 (d, *J* = 4.9 Hz, 3H), 6.98 (d, *J* = 9.0 Hz, 2H), 6.76 (s, 1H), 5.02 (s, 1H), 4.16 (s, 2H), 3.60 (s, 2H), 1.46 (s, 9H); ^13^C NMR (100 MHz, CDCl_3_) *δ* 177.8, 163.1, 163.1, 157.9, 155.9, 131.7, 131.5, 129.0, 127.1, 126.2, 118.0, 114.7, 107.5, 101.0, 79.8, 67.8, 39.9, 28.4; HR-MS (ESI): calcd for C_19_H_16_O_5_ [M + H]^+^ 382.1649, found (ESI^+^) 382.1648.

#### 4.1.3. Synthesis of Compounds **4a**–**4n**, **5a**–**5e**, and **6a**–**6d**

7-(2-Aminoethoxy)-2-phenyl-4*H*-chromen-4-one (**3**). A solution of compound **2f** (0.381 g, 1.0 mmol, 1.0 equiv.) in CH_2_Cl_2_ (3 mL) was added to CF_3_COOH (TFA, 3 mL) and stirred at room temperature for 2 h. After the completion of the reaction, the solvent was removed in vacuum, and the crude product was purified by flash chromatography to obtain crude product **3**·CF_3_COOH. The crude product was washed by saturated NaHCO_3_ solution to obtain compound **3**. White solid, 98% yield; ^1^H NMR (400 MHz, DMSO-*d*_6_) *δ* 8.10 (d, *J* = 6.7 Hz, 2H), 7.94 (d, *J* = 8.8 Hz, 1H), 7.59 (d, *J* = 6.4 Hz, 3H), 7.32 (s, 1H), 7.07 (d, *J* = 8.4 Hz, 1H), 6.96 (s, 1H), 4.09 (t, *J* = 5.4 Hz, 2H), 3.17 (s, 2H), 2.94 (s, 2H); ^13^C NMR (100 MHz, DMSO-*d*_6_) *δ* 176.9, 163.2, 162.7, 157.9, 132.2, 129.6, 126.8, 126.7, 117.9, 115.6, 107.3, 102.1, 68.0.

Compounds **4a**–**4n**: To a stirred solution of **3**·CF_3_COOH (0.395 g, 1 mmol) in CH_2_Cl_2_ (3 mL), diisopropylethylamine (0.387 g, 3 mmol), 1-hydroxybenzotriazole (HOBt) (0.203 g, 1.5 mmol), and 1-(3-dimethylaminopropyl)-3-ethylcarbodiimide hydrochloride (EDCI) (0.288 g, 1.5 mmol) were added, and the reaction mixture was stirred for 30 min at 0 °C. Subsequently, carboxylic acid (1.2 mmol) was added, and the resulting solution was stirred at room temperature for 5 h. After the completion of the reaction, the organic phase was washed with water (10 mL) and dried with MgSO_4_. The crude product was purified by recrystallization to obtain compounds **4a**–**4n**.

Isopropyl(2-oxo-2-((2-((4-oxo-2-phenyl-4*H*-chromen-7-yl)oxy)ethyl)amino)ethyl)carbamate (**4a**). White solid, 56.7% yield, m.p. 143–146 °C; ^1^H NMR (400 MHz, DMSO-*d*_6_) *δ* 8.09 (d, *J* = 7.8 Hz, 3H), 7.94 (d, *J* = 8.8 Hz, 1H), 7.58 (d, *J* = 6.7 Hz, 3H), 7.34 (s, 1H), 7.17 (t, *J* = 5.4 Hz, 1H), 7.06 (d, *J* = 8.8 Hz, 1H), 6.97 (s, 1H), 4.83–4.63 (m, 1H), 4.17 (t, *J* = 4.9 Hz, 2H), 3.60 (d, *J* = 5.8 Hz, 2H), 3.51 (d, *J* = 5.3 Hz, 2H), 1.16 (d, *J* = 6.1 Hz, 6H); ^13^C NMR (100 MHz, DMSO-*d*_6_) *δ* 176.9, 170.2, 163.5, 162.7, 157.9, 156.7, 132.2, 131.7, 129.6, 126.7, 117.7, 115.5, 107.3, 102.1, 67.7, 67.5, 43.9, 38.5, 22.5; HR-MS (ESI): calcd for C_23_H_24_N_2_O_6_ [M + H]^+^ 425.1707, found (ESI^+^) 425.1704.
Isobutyl(2-oxo-2-((2-((4-oxo-2-phenyl-4*H*-chromen-7-yl)oxy)ethyl)amino)ethyl)carbamate (**4b**). White solid, 53.5% yield, m.p. 141–143 °C; ^1^H NMR (400 MHz, DMSO-*d*_6_) *δ* 8.10 (d, *J* = 7.3 Hz, 3H), 7.95 (d, *J* = 8.8 Hz, 1H), 7.59 (d, *J* = 6.6 Hz, 3H), 7.35 (s, 1H), 7.28 (t, *J* = 5.8 Hz, 1H), 7.07 (d, *J* = 8.7 Hz, 1H), 6.98 (s, 1H), 4.17 (t, *J* = 4.7 Hz, 2H), 3.72 (d, *J* = 6.5 Hz, 2H), 3.60 (d, *J* = 5.7 Hz, 2H), 3.51 (d, *J* = 5.1 Hz, 2H), 1.82 (dt, *J* = 13.6, 6.7 Hz, 1H), 0.87 (d, *J* = 6.5 Hz, 6H); ^13^C NMR (100 MHz, DMSO-*d*_6_) *δ* 177.0, 170.2, 163.5, 162.8, 157.9, 157.3, 129.6, 126.8, 117.7, 115.6, 107.3, 102.1, 70.5, 67.7, 43.9, 38.5, 28.1, 19.4; HR-MS (ESI): calcd for C_24_H_26_N_2_O_6_ [M + H]^+^ 439.1864, found (ESI^+^) 439.1898.
Tert-butyl(2-oxo-2-((2-((4-oxo-2-phenyl-4*H*-chromen-7-yl)oxy)ethyl)amino)ethyl)carbamate (**4c**). White solid, 43.5% yield, m.p. 151–153 °C; ^1^H NMR (400 MHz, DMSO-*d*_6_) *δ* 8.09 (t, *J* = 8.0 Hz, 3H), 7.95 (d, *J* = 8.8 Hz, 1H), 7.59 (d, *J* = 6.5 Hz, 3H), 7.35 (s, 1H), 7.07 (d, *J* = 8.6 Hz, 1H), 6.97 (d, *J* = 5.4 Hz, 2H), 4.17 (s, 2H), 3.53 (dd, *J* = 15.0, 5.5 Hz, 4H), 1.37 (s, 9H); ^13^C NMR (100 MHz, DMSO-*d*_6_) *δ* 177.0, 170.3, 163.5, 162.7, 158.0, 156.3, 132.2, 131.6, 129.6, 126.7, 117.7, 115.6, 107.2, 102.0, 78.6, 67.7, 43.7, 38.5, 28.7; HR-MS (ESI): calcd for C_24_H_26_N_2_O_6_ [M + H]^+^ 439.1864, found (ESI^+^) 439.1893.
(*S*)-Isopropyl(1-oxo-1-((2-((4-oxo-2-phenyl-4*H*-chromen-7-yl)oxy)ethyl)amino)propan-2-yl)carbamate (**4d**). White solid, 37.5% yield, m.p. 134–136 °C; ${\left[\alpha \right]}_{D}^{21}$ = +0.6° (*c* 1.0, CH_3_OH); ^1^H NMR (400 MHz, DMSO-*d*_6_) *δ* 8.08 (d, *J* = 6.8 Hz, 3H), 7.94 (d, *J* = 8.7 Hz, 1H), 7.58 (d, *J* = 6.4 Hz, 3H), 7.32 (s, 1H), 7.13 (d, *J* = 6.9 Hz, 1H), 7.05 (d, *J* = 8.8 Hz, 1H), 6.96 (s, 1H), 4.87 –4.54 (m, 1H), 4.16 (s, 2H), 4.10–3.94 (m, 1H), 3.61–3.43 (m, 2H), 1.18 (d, *J* = 7.0 Hz, 3H), 1.14 (d, *J* = 5.7 Hz, 6H); ^13^C NMR (100 MHz, DMSO-*d*_6_) *δ* 176.9, 173.6, 163.5, 162.7, 157.9, 155.9, 132.1, 131.6, 129.5, 126.7, 126.6, 117.7, 115.5, 107.2, 102.0, 67.6, 67.4, 50.4, 38.5, 22.5, 18.7; HR-MS (ESI): calcd for C_24_H_26_N_2_O_6_ [M + H]^+^ 439.1864, found (ESI^+^) 439.1891.
(*S*)-Isopropyl(3-methyl-1-oxo-1-((2-((4-oxo-2-phenyl-4*H*-chromen-7-yl)oxy)ethyl)amino) butan-2-yl) carbamate (**4e**). White solid, 41.3% yield, m.p. 164–168 °C; ${\left[\alpha \right]}_{D}^{21}$ = +3.2° (*c* 1.0, CH_3_OH); ^1^H NMR (400 MHz, DMSO-*d*_6_) *δ* 8.17 (s, 1H), 8.10 (d, *J* = 6.9 Hz, 2H), 7.95 (d, *J* = 8.8 Hz, 1H), 7.59 (d, *J* = 6.4 Hz, 3H), 7.34 (s, 1H), 7.04 (d, *J* = 8.8 Hz, 1H), 6.97 (s, 1H), 6.92 (d, *J* = 8.4 Hz, 1H), 4.71 (p, *J* = 5.9 Hz, 1H), 4.17 (s, 2H), 3.81 (t, *J* = 7.6 Hz, 1H), 3.52 (ddd, *J* = 45.7, 14.0, 5.8 Hz, 2H), 2.01–1.81 (m, 1H), 1.14 (d, *J* = 5.1 Hz, 6H), 0.81 (d, 6H); ^13^C NMR (100 MHz, DMSO-*d*_6_) *δ* 176.4, 171.7, 163.0, 162.2, 157.4, 155.8, 131.6, 131.1, 129.1, 126.2, 117.2, 115.0, 106.7, 101.5, 66.9, 60.1, 37.9, 30.2, 22.0, 19.1, 18.2; HR-MS (ESI): calcd for C_26_H_30_N_2_O_6_ [M + H]^+^ 467.2177, found (ESI^+^) 467.2179.
(*S*)-Isopropyl(4-methyl-1-oxo-1-((2-((4-oxo-2-phenyl-4*H*-chromen-7-yl)oxy)ethyl)amino) pentan-2-yl) carbamate (**4f**). White solid, 40.5% yield, m.p. 132–136 °C; ${\left[\alpha \right]}_{D}^{21}$ = −7.6° (*c* 1.0, CH_3_OH); ^1^H NMR (400 MHz, DMSO-*d*_6_) *δ* 8.19–8.04 (m, 3H), 7.95 (d, *J* = 8.8 Hz, 1H), 7.59 (d, *J* = 6.3 Hz, 3H), 7.33 (s, 1H), 7.06 (t, *J* = 9.9 Hz, 2H), 6.97 (s, 1H), 4.70 (dt, *J* = 11.9, 5.9 Hz, 1H), 4.17 (s, 2H), 3.99 (d, *J* = 6.0 Hz, 1H), 3.49 (dd, *J* = 20.4, 3.9 Hz, 2H), 1.61–1.51 (m, 1H), 1.38 (dt, *J* = 14.0, 7.8 Hz, 2H), 1.13 (d, *J* = 5.7 Hz, 6H), 0.81 (t, *J* = 6.5 Hz, 6H); ^13^C NMR (100 MHz, DMSO-*d*_6_) *δ* 176.4, 172.9, 163.0, 162.2, 157.4, 155.6, 131.6, 131.1, 129.1, 126.2, 117.2, 115.0, 106.7, 101.5, 67.1, 66.9, 53.0, 40.8, 38.0, 24.2, 22.8, 21.5; HR-MS (ESI): calcd for C_27_H_32_N_2_O_6_ [M + H]^+^ 481.2333, found (ESI^+^) 481.2343.
Isopropyl((2*S*,3*S*)-3-methyl-1-oxo-1-((2-((4-oxo-2-phenyl-4*H*-chromen-7-yl)oxy)ethyl) amino)pentan-2-yl)carbamate (**4g**). White solid, 37.5% yield, m.p. 180–187 °C; ${\left[\alpha \right]}_{D}^{21}$ = +1.6° (*c* 1.0, CH_3_OH); ^1^H NMR (400 MHz, DMSO-*d*_6_) *δ* 8.18 (s, 1H), 8.10 (d, *J* = 6.8 Hz, 2H), 7.95 (d, *J* = 8.9 Hz, 1H), 7.59 (d, *J* = 6.4 Hz, 3H), 7.34 (s, 1H), 7.04 (d, *J* = 8.7 Hz, 1H), 7.00–6.89 (m, 2H), 4.71 (dt, *J* = 11.7, 5.8 Hz, 1H), 4.17 (s, 2H), 3.84 (t, *J* = 7.8 Hz, 1H), 3.51 (dd, *J* = 35.8, 4.9 Hz, 2H), 1.66 (s, 1H), 1.39 (s, 2H), 1.13 (d, *J* = 5.1 Hz, 6H), 0.82–0.74 (m, 6H); ^13^C NMR (100 MHz, DMSO-*d*_6_) *δ* 176.9, 172.2, 163.5, 162.7, 157.9, 156.2, 132.2, 131.6, 129.6, 126.7, 117.7, 115.5, 107.2, 102.0, 67.6, 67.4, 59.5, 38.4, 36.8, 24.9, 22.5, 15.7, 11.4; HR-MS (ESI): calcd for C_27_H_32_N_2_O_6_ [M + H]^+^ 481.2333, found (ESI^+^) 481.2338.
(Isopropyl((2*S*,3*R*)-3-hydroxy-1-oxo-1-((2-((4-oxo-2-phenyl-4*H*-chromen-7-yl)oxy)ethyl) amino)butan-2-yl)carbamate (**4h**). White solid, 33.0% yield, m.p. 134–137 °C; ${\left[\alpha \right]}_{D}^{21}$ = +4.5° (*c* 1.0, CH_3_OH); ^1^H NMR (400 MHz, DMSO-*d*_6_) *δ* 8.12 (d, *J* = 5.8 Hz, 3H), 7.97 (d, *J* = 8.8 Hz, 1H), 7.62 (s, 3H), 7.36 (s, 1H), 7.08 (d, *J* = 8.5 Hz, 1H), 6.99 (s, 1H), 6.58 (d, *J* = 7.4 Hz, 1H), 4.92–4.65 (m, 2H), 4.20 (s, 2H), 3.93 (d, *J* = 6.2 Hz, 2H), 3.54 (dd, *J* = 18.9, 5.0 Hz, 2H), 1.28–1.12 (m, 6H), 1.05 (d, *J* = 4.6 Hz, 3H); ^13^C NMR (100 MHz, DMSO-*d*_6_) *δ* 171.3, 163.5, 162.7, 156.3, 132.2, 131.7, 129.6, 126.7, 115.6, 107.3, 67.7, 67.7, 67.2, 61.1, 38.6, 22.5, 20.5; HR-MS (ESI): calcd for C_25_H_28_N_2_O_7_ [M + H]^+^ 469.1970, found (ESI^+^) 469.1977.
(*S*)-Isopropyl(2-oxo-2-((2-((4-oxo-2-phenyl-4*H*-chromen-7-yl)oxy)ethyl)amino)-1-phenylethyl)carbamate (**4i**).White solid, 47.5% yield, m.p. 131–134 °C; ${\left[\alpha \right]}_{D}^{21}$ = +17.0° (*c* 1.0, CH_3_OH); ^1^H NMR (400 MHz, DMSO-*d*_6_) *δ* 8.48 (s, 1H), 8.09 (s, 2H), 7.94 (d, *J* = 8.7 Hz, 1H), 7.60 (s, 4H), 7.41 (d, *J* = 7.0 Hz, 2H), 7.32–7.21 (m, 4H), 7.02 (d, *J* = 8.7 Hz, 2H), 5.25 (d, *J* = 8.0 Hz, 1H), 4.92–4.53 (m, 1H), 4.16 (s, 2H), 3.50 (s, 2H), 1.20–1.07 (m, 6H); ^13^C NMR (100 MHz, DMSO-*d*_6_) *δ* 177.0, 170.9, 163.4, 162.7, 157.9, 155.8, 139.1, 131.6, 129.6, 128.7, 128.0, 127.6, 126.7, 126.6, 117.7, 115.5, 107.2, 101.9, 67.7, 67.5, 58.4, 38.7, 22.4; HR-MS (ESI): calcd for C_29_H_28_N_2_O_6_ [M + H]^+^ 501.2020, found (ESI^+^) 501.2023.
(*S*)-Isobutyl(2-oxo-2-((2-((4-oxo-2-phenyl-4*H*-chromen-7-yl)oxy)ethyl)amino)-1-phenylethyl)carbamate (**4j**). White solid, 49.0% yield, m.p. 130–133 °C; ${\left[\alpha \right]}_{D}^{21}$ = +26.0° (*c* 1.0, CH_3_OH); ^1^H NMR (400 MHz, DMSO-*d*_6_) *δ* 8.52 (s, 1H), 8.10 (d, *J* = 5.9 Hz, 2H), 7.94 (d, *J* = 8.7 Hz, 1H), 7.69 (d, *J* = 7.7 Hz, 1H), 7.60 (s, 3H), 7.42 (d, *J* = 7.1 Hz, 2H), 7.33–7.19 (m, 4H), 7.01 (d, *J* = 8.5 Hz, 1H), 6.97 (s, 1H), 5.25 (d, *J* = 8.2 Hz, 1H), 4.16 (s, 2H), 3.72 (d, *J* = 6.1 Hz, 2H), 3.51 (s, 2H), 1.98–1.68 (m, 1H), 0.86 (d, *J* = 5.5 Hz, 6H); ^13^C NMR (100MHz, DMSO-*d*_6_) *δ* 176.92, 170.85, 163.41, 162.68, 157.88, 156.35, 139.04, 132.15, 131.61, 129.56, 128.66, 127.62, 126.66, 117.67, 115.48, 107.23, 102.01, 70.56, 67.51, 58.53, 38.69, 28.05, 19.31; HR-MS (ESI): calcd for C_30_H_30_N_2_O_6_ [M + H]^+^ 515.2177, found (ESI^+^) 515.2170.
(*S*)-Tert-butyl(1-oxo-1-((2-((4-oxo-2-phenyl-4*H*-chromen-7-yl)oxy)ethyl)amino)-3-phenylpropan-2-yl)carbamate (**4k**). White solid, 42.5% yield, m.p. 120–125 °C; ${\left[\alpha \right]}_{D}^{21}$ = −8.8° (*c* 1.0, CH_3_OH); ^1^H NMR (400 MHz, DMSO-*d*_6_) *δ* 8.21 (s, 1H), 8.10 (d, *J* = 5.3 Hz, 2H), 7.96 (d, *J* = 8.7 Hz, 1H), 7.60 (s, 3H), 7.34 (s, 1H), 7.23 (s, 4H), 7.15 (s, 1H), 7.07 (d, *J* = 8.2 Hz, 1H), 6.97 (s, 1H), 6.91 (d, *J* = 8.1 Hz, 1H), 4.14 (s, 3H), 3.51 (d, *J* = 22.2 Hz, 2H), 3.04–2.62 (m, 2H), 1.28 (s, 9H); ^13^C NMR (100 MHz, DMSO-*d*_6_) *δ* 177.1, 172.6, 163.6, 162.8, 158.0, 155.7, 138.5, 132.3, 131.6, 129.6, 128.5, 126.7, 117.7, 115.6, 107.2, 102.0, 78.6, 67.6, 56.2, 38.6, 38.1, 28.6; HR-MS (ESI): calcd for C_31_H_32_N_2_O_6_ [M + H]^+^ 529.2333, found (ESI^+^) 529.2330.
(*S*)-Isopropyl(3-(4-hydroxyphenyl)-1-oxo-1-((2-((4-oxo-2-phenyl-4*H*-chromen-7-yl)oxy)ethyl)amino)propan-2-yl)carbamate (**4l**). White solid, 37.2% yield, m.p. 160–164 °C; ${\left[\alpha \right]}_{D}^{21}$ = −7.9° (*c* 1.0, CH_3_OH); ^1^H NMR (400 MHz, DMSO-*d*_6_) *δ* 8.30 (s, 1H), 8.20–8.07 (m, 2H), 7.98 (d, *J* = 8.8 Hz, 1H), 7.61 (d, *J* = 6.6 Hz, 3H), 7.36 (d, *J* = 2.1 Hz, 1H), 7.30 (d, *J* = 8.1 Hz, 2H), 7.22 (d, *J* = 8.5 Hz, 1H), 7.09 (d, *J* = 8.0 Hz, 3H), 7.00 (d, *J* = 1.7 Hz, 1H), 4.83 (p, *J* = 6.3 Hz, 1H), 4.65 (p, *J* = 6.4 Hz, 1H), 4.24 (s, 1H), 4.15 (q, *J* = 5.6 Hz, 2H), 3.35 (d, *J* = 1.9 Hz, 2H), 3.04–2.70 (m, 2H), 1.28 (d, *J* = 6.2 Hz, 6H); ^13^C NMR (100 MHz, DMSO-*d*_6_) *δ* 176.4, 163.0, 162.2, 157.4, 152.5, 149.2, 135.7, 131.7, 131.1, 130.2, 129.1, 126.2, 120.7, 117.2, 115.0, 106.7, 72.6, 66.9, 55.9, 21.3; HR-MS (ESI): calcd for C_30_H_31_N_2_O_7_ [M + H]^+^ 531.2126, found (ESI^+^) 531.2121.
(*S*)-Isopropyl(3-(1*H*-indol-3-yl)-1-oxo-1-((2-((4-oxo-2-phenyl-4*H*-chromen-7-yl)oxy)ethyl) amino)propan-2-yl)carbamate (**4m**). White solid, 54.3% yield, m.p. 105–115 °C; ${\left[\alpha \right]}_{D}^{21}$ = +2.5° (*c* 1.0, CH_3_OH); ^1^H NMR (400 MHz, DMSO-*d*_6_) *δ* 10.79 (s, 1H), 8.23 (s, 1H), 8.11 (d, *J* = 6.8 Hz, 2H), 7.95 (d, *J* = 8.7 Hz, 1H), 7.59 (d, *J* = 6.0 Hz, 4H), 7.30 (d, *J* = 6.7 Hz, 2H), 7.13 (s, 1H), 7.05 (d, *J* = 10.0 Hz, 3H), 6.96 (d, *J* = 10.6 Hz, 2H), 4.55–4.76 (m, 1H), 4.24 (d, *J* = 2.9 Hz, 1H), 4.10 (s, 2H), 3.47 (s, 2H), 3.11–2.84 (m, 2H), 1.08 (dd, *J* = 21.3, 5.8 Hz, 6H); ^13^C NMR (100 MHz, DMSO-*d*_6_) δ 176.5, 172.3, 163.0, 162.2, 157.4, 155.5, 136.0, 131.7, 131.1, 129.1, 127.2, 126.2, 123.7, 120.8, 118.4, 118.1, 117.2, 116.2, 115.0, 111.2, 110.0, 106.7, 101.5, 67.0, 66.9, 55.4, 38.0, 27.8, 21.9; HR-MS (ESI): calcd for C_32_H_31_N_3_O_6_ [M + H]^+^ 554.2286, found (ESI^+^) 554.2285.
(*S*)-Diisopropyl(6-oxo-6-((2-((4-oxo-2-phenyl-4*H*-chromen-7-yl)oxy)ethyl)amino)hexane-1,5-diyl)dicarbamate (**4n**). White solid, 43.6% yield, m.p. 169–173 °C; ${\left[\alpha \right]}_{D}^{21}$ = −15.3° (*c* 1.0, CH_3_OH); ^1^H NMR (400 MHz, DMSO-*d*_6_) *δ* 8.10 (d, *J* = 6.8 Hz, 3H), 7.95 (d, *J* = 8.8 Hz, 1H), 7.59 (d, *J* = 6.5 Hz, 3H), 7.34 (s, 1H), 7.05 (t, *J* = 8.0 Hz, 2H), 6.97 (s, 1H), 6.93 (s, 1H), 4.67–4.73 (m, 2H), 4.17 (s, 2H), 3.88–3.93 (m, 1H), 3.43–3.56 (m, 2H), 2.87–2.88 (m, 2H), 1.46–1.54 (m, 2H), 1.32 (d, *J* = 5.9 Hz, 2H), 1.28–1.17 (m, 2H), 1.12 (d, *J* = 6.2 Hz, 12H); ^13^C NMR (100 MHz, DMSO-*d*_6_) *δ* 177.0, 173.0, 163.5, 162.7, 157.9, 156.3, 156.2, 132.2, 131.6, 129.6, 126.7, 117.7, 115.5, 107.2, 102.0, 67.4, 66.9, 55.0, 38.4, 32.1, 29.6, 23.2, 22.5, 22.5; HR-MS (ESI): calcd for C_31_H_39_N_3_O_8_ [M + H]^+^ 582.2810, found (ESI^+^) 582.2807.
*N*-(2-((4-Oxo-2-phenyl-4*H*-chromen-7-yl)oxy)ethyl)benzamide (**5a**). White solid, 42.2% yield, m.p. 132–144 °C; ^1^H NMR (400 MHz, DMSO-*d*_6_) *δ* 8.76 (s, 1H), 8.09 (d, *J* = 6.2 Hz, 2H), 7.94 (d, *J* = 8.7 Hz, 1H), 7.87 (d, *J* = 7.4 Hz, 2H), 7.59 (d, *J* = 5.7 Hz, 3H), 7.52 (d, *J* = 7.1 Hz, 1H), 7.46 (t, *J* = 7.2 Hz, 2H), 7.39 (s, 1H), 7.09 (d, *J* = 8.8 Hz, 1H), 6.97 (s, 1H), 4.31 (d, *J* = 5.1 Hz, 2H), 3.71 (d, *J* = 5.1 Hz, 2H); ^13^C NMR (100 MHz, DMSO-*d*_6_) *δ* 177.0, 167.2, 163.6, 162.7, 156.0, 134.6, 132.2, 131.8, 131.6, 129.6, 128.8, 127.7, 126.7, 117.7, 115.6, 107.2, 102.1, 67.4, 39.2; HR-MS (ESI): calcd for C_24_H_19_NO_4_ [M + H]^+^ 386.1387, found (ESI^+^) 386.1395.
2-Methoxy-*N*-(2-((4-oxo-2-phenyl-4*H*-chromen-7-yl)oxy)ethyl)benzamide (**5b**). White solid, 43.5% yield, m.p. 95–100 °C; ^1^H NMR (400 MHz, DMSO-*d*_6_) *δ* 8.46 (s, 1H), 8.09 (d, *J* = 6.1 Hz, 2H), 7.95 (d, *J* = 8.7 Hz, 1H), 7.80 (d, *J* = 6.9 Hz, 1H), 7.59 (s, 3H), 7.47 (t, *J* = 7.2 Hz, 1H), 7.40 (s, 1H), 7.12 (t, *J* = 8.7 Hz, 2H), 7.03 (t, *J* = 7.3 Hz, 1H), 6.97 (s, 1H), 4.31 (s, 2H), 3.88 (s, 3H), 3.79–3.68 (m, 2H); ^13^C NMR (100 MHz, DMSO-*d*_6_) *δ* 177.1, 165.9, 163.6, 162.8, 158.0, 157.5, 133.1, 132.3, 131.6, 131.0, 129.6, 126.8, 126.7, 122.8, 121.1, 117.7, 115.6, 112.6, 107.2, 102.0, 67.6, 56.4, 38.9; HR-MS (ESI): calcd for C_25_H_21_NO_5_ [M + H]^+^ 416.1493, found (ESI^+^) 416.1485.
2-Chloro-*N*-(2-((4-oxo-2-phenyl-4*H*-chromen-7-yl)oxy)ethyl)benzamide (**5c**). White solid, 39.8% yield, m.p. 148–154 °C; ^1^H NMR (400 MHz, DMSO-*d*_6_) *δ* 8.74 (s, 1H), 8.10 (d, *J* = 6.0 Hz, 2H), 7.96 (d, *J* = 8.8 Hz, 1H), 7.58 (d, *J* = 6.1 Hz, 3H), 7.49 (d, *J* = 7.6 Hz, 1H), 7.45–7.41 (m, 2H), 7.37 (d, *J* = 8.1 Hz, 2H), 7.09 (d, *J* = 8.7 Hz, 1H), 6.97 (s, 1H), 4.30 (s, 2H), 3.68 (d, *J* = 5.1 Hz, 2H); ^13^C NMR (101 MHz, DMSO-*d*_6_) *δ* 177.0, 167.23, 163.5, 162.7, 158.0, 137.3, 132.2, 131.6, 131.3, 130.4, 130.1, 129.6, 129.3, 127.6, 126.7, 117.7, 115.6, 107.3, 102.0, 67.4, 39.0; HR-MS (ESI): calcd for C_24_H_18_ClNO_4_ [M + H]^+^ 420.0997, found (ESI^+^) 420.0995.
2-Bromo-*N*-(2-((4-oxo-2-phenyl-4*H*-chromen-7-yl)oxy)ethyl)benzamide (**5d**). White solid, 37.5% yield, m.p. 138–148 °C; ^1^H NMR (400 MHz, DMSO-*d*_6_) *δ* 8.72 (s, 1H), 8.10 (d, *J* = 6.0 Hz, 2H), 7.96 (d, *J* = 8.8 Hz, 1H), 7.66–7.53 (m, 4H), 7.39 (q, *J* = 12.7, 10.6 Hz, 4H), 7.09 (d, *J* = 8.8 Hz, 1H), 6.97 (s, 1H), 4.30 (s, 2H), 3.81–3.57 (m, 2H); ^13^C NMR (100 MHz, DMSO-*d*_6_) *δ* 177.0, 168.1, 163.5, 162.7, 156.0, 139.4, 133.2, 132.2, 131.6, 131.4, 129.6, 129.3, 128.0, 126.7, 119.4, 117.7, 115.6, 107.3, 102.0, 67.4, 39.0; HR-MS (ESI): calcd for C_24_H_18_BrNO_4_ [M + H]^+^ 464.0492, found (ESI^+^) 464.0496.
2-Nitro-*N*-(2-((4-oxo-2-phenyl-4*H*-chromen-7-yl)oxy)ethyl)benzamide (**5e**). White solid, 40.1% yield, m.p. 166–172 °C; ^1^H NMR (400 MHz, DMSO-*d*_6_) *δ* 9.01 (s, 1H), 8.10 (d, *J* = 6.4 Hz, 2H), 8.04 (d, *J* = 7.9 Hz, 1H), 7.96 (d, *J* = 8.6 Hz, 1H), 7.78 (t, *J* = 7.2 Hz, 1H), 7.70 (d, *J* = 7.4 Hz, 1H), 7.61 (d, *J* = 7.6 Hz, 4H), 7.40 (d, *J* = 8.1 Hz, 1H), 7.10 (d, *J* = 8.6 Hz, 1H), 6.98 (s, 1H), 4.30 (s, 2H), 3.77–3.60 (m, 2H); ^13^C NMR (100 MHz, DMSO-*d*_6_) *δ* 177.0, 166.5, 163.6, 162.8, 158.0, 147.5, 134.2, 132.8, 132.2, 131.6, 131.3, 129.6, 129.5, 126.7, 124.6, 117.7, 115.6, 107.2, 102.0, 67.4, 39.2; HR-MS (ESI): calcd for C_24_H_18_N_2_O_6_ [M + H]^+^ 431.1238, found (ESI^+^) 431.1236.
*N*^1^-(2-((4-Oxo-2-phenyl-4*H*-chromen-7-yl)oxy)ethyl)-*N*^3^-phenylmalonamide (**6a**). White solid, 44.5% yield, m.p. 173–178 °C; ^1^H NMR (400 MHz, DMSO-*d*_6_) *δ* 10.10 (s, 1H), 8.41 (s, 1H), 8.10 (d, *J* = 6.9 Hz, 2H), 7.96 (d, *J* = 8.8 Hz, 1H), 7.62–7.54 (m, 5H), 7.37 (s, 1H), 7.29 (t, *J* = 7.7 Hz, 2H), 7.06 (dt, *J* = 14.8, 8.0 Hz, 2H), 6.98 (s, 1H), 4.21 (s, 2H), 3.55 (d, *J* = 5.0 Hz, 2H), 3.30 (s, 2H); ^13^C NMR (100 MHz, DMSO-*d*_6_) *δ* 177.0, 167.6, 166.1, 163.5, 162.8, 158.0, 139.4, 132.2, 131.6, 129.6, 129.2, 126.7, 123.9, 119.6, 117.7, 115.6, 107.2, 102.1, 67.7, 45.0, 38.8; HR-MS (ESI): calcd for C_26_H_22_N_2_O_5_ [M + H]^+^ 443.1602, found (ESI^+^) 443.1605.
*N*^1^-(2-((4-Oxo-2-phenyl-4*H*-chromen-7-yl)oxy)ethyl)-*N*^3^-(o-tolyl)malonamide (**6b**). White solid, 46.3% yield, m.p. 166–169 °C; ^1^H NMR (400 MHz, DMSO-*d*_6_) *δ* 9.64 (s, 1H), 8.50 (s, 1H), 8.10 (d, *J* = 7.1 Hz, 2H), 7.95 (d, *J* = 8.8 Hz, 1H), 7.57 (dd, *J* = 14.6, 7.5 Hz, 4H), 7.36 (s, 1H), 7.19 (d, *J* = 7.3 Hz, 1H), 7.14 (t, *J* = 7.5 Hz, 1H), 7.06 (q, *J* = 8.4, 7.3 Hz, 2H), 6.97 (s, 1H), 4.22 (s, 2H), 3.57 (d, *J* = 5.0 Hz, 2H), 3.36 (s, 2H), 2.21 (s, 3H); ^13^C NMR (100 MHz, DMSO-*d*_6_) *δ* 176.9, 168.2, 165.9, 163.4, 162.7, 158.0, 136.7, 132.2, 131.7, 130.9, 130.8, 129.6, 126.7, 126.5, 125.3, 124.2, 117.8, 115.5, 107.3, 102.1, 67.7, 44.0, 38.8, 18.2; HR-MS (ESI): calcd for C_27_H_24_N_2_O_5_ [M + H]^+^ 457.1758, found (ESI^+^) 457.1755.
*N*^1^-(2-Chlorophenyl)-*N*^3^-(2-((4-oxo-2-phenyl-4*H*-chromen-7-yl)oxy)ethyl)malonamide (**6c**). White solid, 39.0% yield, m.p. 184–189 °C; ^1^H NMR (400 MHz, DMSO-*d*_6_) *δ* 10.18 (s, 1H), 8.60 (s, 1H), 8.10 (d, *J* = 6.8 Hz, 2H), 8.02 (d, *J* = 8.0 Hz, 1H), 7.94 (d, *J* = 8.8 Hz, 1H), 7.59 (d, *J* = 6.9 Hz, 3H), 7.48 (d, *J* = 8.0 Hz, 1H), 7.36 (s, 1H), 7.31 (t, *J* = 7.7 Hz, 1H), 7.13 (t, *J* = 7.6 Hz, 1H), 7.08 (d, *J* = 8.8 Hz, 1H), 6.97 (s, 1H), 4.30–4.15 (m, 2H), 3.58 (d, *J* = 4.9 Hz, 2H), 3.45 (s, 2H). ^13^C NMR (100 MHz, DMSO-*d*_6_) *δ* 177.0, 168.3, 166.1, 163.5, 162.8, 158.0, 135.2, 132.2, 131.6, 129.9, 129.6, 128.1, 126.7, 126.1, 124.7, 124.1, 117.7, 115.6, 107.2, 102.1, 67.7, 43.6, 38.8; HR-MS (ESI): calcd for C_26_H_21_ClN_2_O_5_ [M + H]^+^ 477.1212, found (ESI^+^) 477.1205.
*N*^1^-(4-Bromophenyl)-*N*3-(2-((4-oxo-2-phenyl-4*H*-chromen-7-yl)oxy)ethyl)malonamide (**6d**). White solid, 42.2% yield, m.p. 181–186 °C; ^1^H NMR (400 MHz, DMSO-*d*_6_) *δ* 10.30 (d, *J* = 5.0 Hz, 1H), 8.50–8.40 (m, 1H), 8.10 (d, *J* = 6.2 Hz, 2H), 7.95 (d, *J* = 8.8 Hz, 1H), 7.64–7.53 (m, 5H), 7.46 (d, *J* = 8.5 Hz, 2H), 7.36 (s, 1H), 7.08 (d, *J* = 8.7 Hz, 1H), 6.97 (s, 1H), 4.20 (s, 2H), 3.54 (d, *J* = 4.8 Hz, 2H), 3.33 (s, 2H); ^13^C NMR (100 MHz, DMSO-*d*_6_) *δ* 176.9, 167.3, 166.3, 163.5, 162.7, 158.0, 138.8, 132.2, 132.0, 131.7, 129.6, 126.7, 121.5, 117.7, 115.5, 115.4, 107.3, 102.1, 67.7, 45.1, 38.8; HR-MS (ESI): calcd for C_26_H_21_BrN_2_O_5_ [M + H]^+^ 521.0707, found (ESI^+^) 521.0713.

### 4.2. Biological Assays

Each test was repeated three times at 25 ± 1 °C. The active effect was expressed in percentage scale of 0–100 (0: no activity; 100: total inhibited). The specific test method for the anti-TMV activity was carried out by the literature method [34,51], and detailed bioassay procedures for the anti-TMV activity were described as follows. The in vivo inhibition rates of the compound were then calculated according to the following formula (1) (“av” means average, and controls were not treated with compound).

Inhibition rate (%) = [(av local lesion no. of control − av local lesion no. of drug-treated)/av local lesion no. of control] × 100%(1)

#### 4.2.1. Extraction of TMV

Using Gooding’s method [52] and our previous reports [7,34,51], the TMV viruses were propagated in *Nicotiana tabacum* L. The infected tobacco leaves were selected and soaked in phosphate buffer, and then filtered through double-layer pledget. The filtrate was centrifuged at 10,000× *g* for 5 min, treated with PEG twice, and centrifuged again, and the extract was processed at 4 °C. The absorbance value was estimated at 260 nm by an ultraviolet spectrophotometer. The concentration of TMV viruses was obtained using Equation (2).

(2) $$Virus\;concn=\left(A260\times dilution\;ratio\right)\;E{/}_{1\mathrm{cm}}^{0.1\%,260\mathrm{nm}}$$

#### 4.2.2. Inactivation Effect of Compounds against TMV In Vivo

After the compound and TMV viruses were mixed for 30 min, the tobacco leaves were sprinkled with diamonds. The mixture was then inoculated on the left side of the leaves of *N. tabacum* L., whereas the right side of the leaves were inoculated with the mixture of solvent and the virus for control. After incubating for half an hour, the leaves were rinsed with water and sequentially incubated at 25 °C in a greenhouse at 25 ± 2 °C. The local lesion numbers were counted after 3–4 days. There were three replicates for each compound.

#### 4.2.3. Cultivation Effect of Compounds against TMV In Vivo

Growing leaves of *N. tabacum* L. of the same ages were selected and sprinkled with diamonds. TMV (concentration of 6.0 × 10^−3^ mg/mL) was dipped and inoculated on the whole leaves. After inoculation with the TMV viruses for half an hour at 25 °C, the leaves were washed under running water, then allowed to air dry. The compound solution was smeared on the left side, and the solvent was smeared on the right side for control. The local lesion numbers were then counted and recorded 3–4 days after inoculation.

#### 4.2.4. Protective Effect of Compounds against TMV In Vivo

The compound solution was smeared on the left side and the solvent serving as the control was smeared on the right side of growing *N. tabacum* L. leaves of the same ages. The leaves were then inoculated with the virus after 12 h. A brush was dipped in TMV of 6 × 10^−3^ mg/mL to inoculate the leaves, which were previously scattered with silicon carbide. The leaves were rubbed softly along the nervature once or twice and then washed with water. The local lesion numbers appearing 3–4 days after inoculation were counted. There were three replicates for each compound.

### 4.3. Calculation Procedures for Molecular Docking Research

The calculation procedures for molecular docking research consisted of four steps according to the literature method [48,53] and our previously described method [7,34].

Receptor Preparation. The 3D crystal structure of TMV-CP (PDB code:1EI7) was downloaded from the protein data bank, and this was used as the receptor for molecular docking. Water molecules were removed from the target protein and hydrogen atoms were added using AutoDock Tools prior to molecular docking.

Ligand preparation. Target compounds are drawn using ChemOffice 2015 as ligands followed by management of its conformer and the minimization process.

Molecular Docking Using AutoDock Vina 1.1.2. The input files for AutoDock Vina were prepared using AutoDock Tools. The protein was placed in a grid box (grid parameters: center x = 5, center y = −20, center z = 0.8, size x = 60, size y = 60, size z = 56), using AutoDock Vina 1.1.2 at 1.00 Å to define the binding site. The docking procedure was performed using the instructed command prompts.

Analysing and Output Visualization using PyMOL 1.7.2.1. The docking poses were ranked according to their docking scores. The scoring function in Auto Dock was used to predict the binding affinity of one ligand to the receptor molecule. The conformation with the lowest binding affinity was selected for further analysis after the docking process. The docking results included the locations of hydrogen bonds, and closely interacting residues were performed by PyMOL software 1.7.2.1.

## 5. Conclusions

In this paper, a series of flavone derivatives containing carboxamide fragments were synthesized based on the natural product and evaluated for their antiviral activities against TMV. Most of these compounds displayed good to excellent anti-TMV activities in vivo. The structure–activity relationship revealed that the introduction of amino acids, aromatic carboxylic acids, and malonic acid derivative fragments to the mother nucleus structure on the 7-positions were favorable for their activities. In particular, compound **4m** (inactivation activity, 58%; curative activity, 57%; protection activity, 59%) even exhibited similar anti-TMV activity with ningnanmycin (inactivation activity, 61%; curative activity, 57%; protection activity, 58%) at 500 μg/mL, which was significantly higher than that of ribavirin (inactivation activity, 39%; curative activity, 40%; protection activity, 39%). Antiviral mechanism research by molecular docking demonstrated that these flavone derivatives containing carboxamide fragments could interact with TMV CP at multiple sites and inhibit virus assembly. In this study, it was found that the introduction of carboxamide fragments at the 7-position of the flavonoids greatly improved the antiviral activity, which has the potential to become a new type of anti-plant virus agent.

## Figures and Tables

**Table 1:** In vivo antiviral activity of compounds **2a**–**2e**, **4a**–**4n**, **5a**–**5e**, and **6a**–**6d** against TMV.

Compounds	Concentrations (mg/L)	Anti-TMV Activities (%) ^a^		
		Inactivation Effect	Curative Effect	Protection Effect
**1**	500	32 ± 1	30 ± 3	30 ± 3
	100	0	0	0
**2a**	500	44 ± 2	40 ± 2	41 ± 1
	100	23 ± 1	16 ± 2	18 ± 2
**2b**	500	47 ± 2	42 ± 1	43 ± 1
	100	23 ± 2	15 ± 2	18 ± 3
**2c**	500	43 ± 2	41 ± 2	41 ± 1
	100	17 ± 1	14 ± 2	16 ± 2
**2d**	500	53 ± 1	50 ± 2	52 ± 2
	100	20 ± 2	18 ± 3	20 ± 3
**2e**	500	49 ± 2	50 ± 1	53 ± 2
	100	24 ± 2	22 ± 3	21 ± 1
**2f**	500	50 ± 3	47 ± 1	48 ± 3
	100	18 ± 2	16 ± 2	19 ± 3
**4a**	500	55 ± 2	53 ± 2	53 ± 1
	100	19 ± 2	21 ± 3	23 ± 2
**4b**	500	52 ± 2	50 ± 2	50 ± 1
	100	36 ± 2	33 ± 1	35 ± 2
**4c**	500	50 ± 1	47 ± 1	48 ± 2
	100	18 ± 1	16 ± 1	19 ± 2
**4d**	500	48 ± 2	45 ± 1	47 ± 1
	100	15 ± 2	14 ± 2	14 ± 2
**4e**	500	39 ± 1	37 ± 2	39 ± 3
	100	0	0	0
**4f**	500	38 ± 1	36 ± 2	36 ± 1
	100	0	0	0
**4g**	500	43 ± 2	40 ± 1	41 ± 1
	100	16 ± 2	14 ± 2	15 ± 1
**4h**	500	52 ± 3	50 ± 2	50 ± 1
	100	34 ± 3	31 ± 2	32 ± 3
**4i**	500	48 ± 2	45 ± 1	47 ± 2
	100	16 ± 1	14 ± 2	16 ± 2
**4j**	500	42 ± 3	41 ± 1	43 ± 2
	100	12 ± 2	13 ± 3	11 ± 1
**4k**	500	47 ± 1	45 ± 1	46 ± 2
	100	19 ± 2	17 ± 3	16 ± 1
**4l**	500	44 ± 3	40 ± 2	41 ± 2
	100	14 ± 1	13 ± 2	15 ± 2
**4m**	500	58 ± 2	57 ± 2	59 ± 3
	100	23 ± 1	24 ± 3	27 ± 1
**4n**	500	52 ± 1	48 ± 2	51 ± 2
	100	29 ± 1	26 ± 1	27 ± 2
**5a**	500	53 ± 2	55 ± 1	54 ± 3
	100	20 ± 1	21 ± 2	20 ± 1
**5b**	500	45 ± 4	44 ± 2	46 ± 2
	100	18 ± 3	16 ± 1	17 ± 1
**5c**	500	49 ± 3	52 ± 2	51 ± 2
	100	20 ± 1	19 ± 3	18 ± 3
**5d**	500	48 ± 2	52 ± 4	50 ± 4
	100	15 ± 1	21 ± 2	18 ± 2
**5e**	500	53 ± 3	54 ± 4	52 ± 3
	100	20 ± 4	21 ± 3	19 ± 2
**6a**	500	43 ± 3	45 ± 4	43 ± 3
	100	19 ± 2	17 ± 3	15 ± 2
**6b**	500	54 ± 4	57 ± 3	54 ± 3
	100	20 ± 2	23 ± 2	19 ± 2
**6c**	500	46 ± 3	47 ± 1	45 ± 4
	100	15 ± 2	17 ± 2	15 ± 3
**6d**	500	45 ± 3	47 ± 2	49 ± 3
	100	20 ± 1	21 ± 3	22 ± 3
Ningnanmycin	500	61 ± 2	57 ± 2	58 ± 2
	100	27 ± 1	24 ± 3	26 ± 2
Ribavirin	500	39 ± 1	40 ± 2	39 ± 2
	100	44 ± 2	40 ± 2	41 ± 1

^a^ Average of three replicates. All results are expressed as mean ± SD.

## Data Availability

All data used to support the findings of this study are included within the article and Supplementary Materials.

## Supplementary Materials

The following supporting information can be downloaded at https://www.mdpi.com/article/10.3390/molecules28052179/s1, Section S1: Phytotoxic activity; Section S2: Copies of NMR spectra (Figures S1–S60); Section S3: Molecule docking results of **1** and **2a** with TMV CP (Figure S61). References [7,51] were cited in Supplementary Materials.

## Sample Availability

Samples of the compounds **2a**–**2e**, **4a**–**4n**, **5a**–**5e**, and **6a**–**6d** are available from the authors.

## References

[1] Savary S., Willocquet L., Pethybridge S.J., Esker P., Mcroberts N., Nelson A. (2019). The global burden of pathogens and pests on major food crops. Nat. Ecol. Evol..

[2] Abraham A., Philip S., Jacob M.K., Narayanan S.P., Jacob C.K., Kochupurackal J. (2015). Phenazine-1-carboxylic acid mediated anti-oomycete activity of the endophytic Alcaligenes sp. EIL-2 against *Phytophthora meadii*. Microbiol. Res..

[3] Huang D., Wang S., Song D., Cao X., Huang W., Ke S. (2020). Discovery of γ-Lactam alkaloid derivatives as potential fungicidal agents targeting steroid biosynthesis. J. Agric. Food Chem..

[4] Guo J., Hao Y., Ji X., Wang Z., Liu Y., Ma D., Li Y., Pang H., Ni J., Wang Q. (2019). Optimization, structure–activity relationship, and mode of action of nortopsentin analogues containing thiazole and oxazole moieties. J. Agric. Food Chem..

[5] Yang S., Wang T., Zhou Y., Shi L., Lu A., Wang Z. (2021). Discovery of cysteine and its derivatives as novel antiviral and antifungal agents. Molecules.

[6] Guo W., Yan H., Ren X., Tang R., Sun Y., Wang Y., Feng J. (2019). Berberine induces resistance against *tobacco mosaic virus* in tobacco. Pest Manag. Sci..

[7] Wang T., Yang S., Li H., Lu A., Wang Z., Yao Y., Wang Q. (2019). Discovery, structural optimization, and mode of action of essramycin alkaloid and its derivatives as anti-tobacco mosaic virus and anti-phytopathogenic fungus agents. J. Agric. Food Chem..

[8] Scholthof K.-B.G. (2004). Tobacco mosaic virus: A model system for plant biology. Annu. Rev. Phytopathol..

[9] Reichman M., Devash Y., Suhadolnik R.J., Sela I. (1983). Human leukocyte interferon and the antiviral factor (AVF) from virus-infected plants stimulate plant tissues to produce nucleotides with antiviral activity. Virology.

[10] Gan X., Wang Y., Hu D., Song B. (2017). Design, synthesis, and antiviral activity of novel chalcone derivatives containing a purine moiety. Chin. J. Chem..

[11] Cai L., Zhang W., Jia H., Feng H., Wei X., Chen H., Wang D., Xue Y., Sun X. (2020). Plant-derived compounds: A potential source of drugs against Tobacco mosaic virus. Pestic. Biochem. Physiol..

[12] Cantrell C.L., Dayan F.E., Duke S.O. (2012). Natural products as sources for new pesticides. J. Nat. Prod..

[13] Newman D.J., Cragg G.M. (2020). Natural products as sources of new drugs over the nearly four decades from 01/1981 to 09/2019. J. Nat. Prod..

[14] Rodrigues T., Reker D., Schneider P., Schneider G. (2016). Counting on natural products for drug design. Nat. Chem..

[15] Yao H., Liu J., Xu S., Zhu Z., Xu J. (2016). The structural modification of natural products for novel drug discovery. Expert Opin. Drug Discov..

[16] Mihorianu M., Franz M.H., Jones P.G., Freytag M., Kelter G., Fiebig H.-H., Tamm M., Neda I. (2016). N-Heterocyclic carbenes derived from imidazo-[1,5-*a*]pyridines related to natural products: Synthesis, structure and potential biological activity of some corresponding gold(I) and silver(I) complexes. Appl. Organomet. Chem..

[17] Sparks T.C., Duke S.O. (2021). Structure simplification of natural products as a lead generation approach in agrochemical discovery. J. Agric. Food Chem..

[18] Song B.A., Zhang G.P., Hu D.Y., Pang L., Yang S., Liu G., Wang H. (2005). N-Substituted Benzothiazolyl-1-substitutedphenyl-O,O-dialkyl-α-aminophosphonate Ester Derivatives Preparation and Application. China Patent.

[19] Chen Z., Li G.J., Fan H.T., Liu J.J., Bi L., Yang S., Song B.A., Hu D.Y. (2011). The study of efficiency of dufulin against southern rice black-streaked dwarf virus. Chin. Agr. Sci. Bull..

[20] Martens S., Mithöfer A. (2005). Flavones and flavone synthases. Phytochemistry.

[21] Shamsudin N.F., Ahmed Q.U., Mahmood S., Shah S.A.A., Khatib A., Mukhtar S., Alsharif M.A., Parveen H., Zakaria Z.A. (2022). Antibacterial effects of flavonoids and their structure-activity relationship study: A comparative interpretation. Molecules.

[22] Jin Y.-S. (2019). Recent advances in natural antifungal flavonoids and their derivatives. Bioorganic Med. Chem. Lett..

[23] Orhan D.D., Özçelik B., Özgen S., Ergun F. (2010). Antibacterial, antifungal, and antiviral activities of some flavonoids. Microbiol. Res..

[24] Al-Khayri J.M., Sahana G.R., Nagella P., Joseph B.V., Alessa F.M., Al-Mssallem M.Q. (2022). Flavonoids as potential anti-inflammatory molecules: A review. Molecules.

[25] Benavente-García O., Castillo J. (2008). Update on uses and properties of Citrus flavonolds: New findings in anticancer, cardiovascular, and anti-inflammatory activity. J. Agric. Food Chem..

[26] Jia L.-G., Sheng Z.-W., Xu W.-F., Li Y.-X., Liu Y.-G., Xia Y.-J., Zhang J.-H. (2012). Modulation of anti-oxidation ability by proanthocyanidins during germination of *Arabidopsis thaliana* seeds. Mol. Plant.

[27] Kashiwada Y., Aoshima A., Ikeshiro Y., Chen Y.-P., Furukawa H., Itoigawa M., Fujioka T., Mihashi K., Cosentino L.M., Morris-Natschke S.L. (2005). Anti-HIV benzylisoquinoline alkaloids and flavonoids from the leaves of *Nelumbo nucifera*, and structure–activity correlations with related alkaloids. Bioorganic Med. Chem..

[28] Krcatović E., Rusak G., Bezić N., Krajacić M. (2008). Inhibition of tobacco mosaic virus infection by quercetin and vitexin. Acta Virol..

[29] Zhao W., Zeng X., Zhang T., Wang L., Yang G., Chen Y.-K., Hu Q., Miao M. (2013). Flavonoids from the bark and stems of Cassia fistula and their anti-tobacco mosaic virus activities. Phytochem. Lett..

[30] Li Y., Ye S., Hu Z., Hao N., Bo X., Liang H., Tian X. (2021). Identification of anti- TMV active flavonoid glycosides and their mode of action on virus particles from *Clematis lasiandra* Maxim. Pest Manag. Sci..

[31] Chen Z., Tan J., Yang G., Miao M., Chen Y., Li T. (2012). Isoflavones from the roots and stems of Nicotiana Tabacum and their anti-tobacco mosaic virus activities. Phytochem. Lett..

[32] Kong W.-S., Xing H.-H., Li J., Ye L., Liu X., Li Y.-P., Rao G.-X., Zhou M., Yang G., Hu Q.-F. (2018). Two new flavones from *Cassia pumila* and their anti-tobacco mosaic virus activity. Chem. Nat. Compd..

[33] Yao J.M., Pu L.L., Rao J.R., Wang M., Guo C., Lei Z.W. (2021). Research progress of anti-plant virus agents based on structural diversification. Agrochemicals.

[34] Ma Y.C., Wang L., Lu A.D., Xue W. (2022). Synthesis and biological activity of novel oxazinyl flavonoids as antiviral and anti-phytopathogenic fungus agents. Molecules.

[35] Wei G., Gao M.Q., Zhu X.L., Yang G.F. (2019). Research progress on carboxamide fungicides targeting succinate dehydrogenase. Chin. J. Pestic. Sci..

[36] Luo B., Ning Y. (2022). Comprehensive overview of carboxamide derivatives as succinate dehydrogenase inhibitors. J. Agric. Food Chem..

[37] Chaparro S., Rojas H.A., Castillo J.C., Portilla J., Romanelli G.P., Pineda A., Elsharif A.M., Martinez J.J., Luque R. (2020). Solventless amide synthesis catalyzed by biogenic CaCO_3_ materials. ACS Sustain. Chem. Eng..

[38] Zeng S., Liu J., Anankanbil S., Chen M., Guo Z., Adams J.P., Snajdrova R., Li Z. (2018). Amide synthesis via aminolysis of ester or acid with an intracellular lipase. ACS Catal..

[39] Serdiuk I.E., Roshal A.D. (2015). Single and double intramolecular proton transfers in the electronically excited state of flavone derivatives. RSC Adv..

[40] Trott O., Olson A.J. (2010). AutoDock Vina: Improving the speed and accuracy of docking with a new scoring function, efficient optimization, and multithreading. J. Comput. Chem..

[41] Liu J., Taylor S.F., Dupart P.S., Arnold C.L., Sridhar J., Jiang Q., Wang Y., Skripnikova E.V., Zhao M., Maryam Foroozesh M. (2013). Pyranoflavones: A group of small-molecule probes for exploring the active site cavities of cytochrome P450 enzymes 1A1, 1A2, and 1B1. J. Med. Chem..

[42] Du X.-J., Bian Q., Wang H.-X., Yu S.-J., Kou J.-J., Wang Z.-P., Li Z.-M., Zhao W.-G. (2014). Design, synthesis, and fungicidal activity of novel carboxylic acid amides represented by *N*-benzhydryl valinamode carbamates. Org. Biomol. Chem..

[43] Sun M., Yang H.-H., Tian L., Li J.-Q., Zhao W.-G. (2015). Design, synthesis, and fungicidal activities of imino diacid analogs of valine amide fungicides. Bioorganic Med. Chem. Lett..

[44] Atalay S.S., Assad M.Y., Amagata T., Wu W. (2020). Mild, efficient, and solvent-free synthesis of 4-hydroxy-2-quinolinones. Tetrahedron Lett..

[45] Lamberth C. (2010). Amino acid chemistry in crop protection. Tetrahedron.

[46] Ryzhkov V.L. (1951). Effect of amino acids and related substances on reproduction of the tobacco mosaic virus. Dokl. Akad. Nauk SSSR.

[47] Zhang B., Li L., Liu Y., Wang Q. (2016). Antiviral mechanism study of gossypol and its Schiff base derivatives based on reactive oxygen species (ROS). RSC Adv..

[48] Seyedi S.S., Shukri M., Hassandarvish P., Oo A., Shankar E.M., Abubakar S., Zandi K. (2016). Computational approach towards exploring potential anti-chikungunya activity of selected flavonoids. Sci. Rep..

[49] Bhyravbhatla B., Watowich S.J., Caspar D.L. (1998). Refined atomic model of the four-layer aggregate of the tobacco mosaic virus coat protein at 2.4-Å resolution. Biophys. J..

[50] Takamatsu Y., Sugiyama A., Purqon A., Nagao H., Nishikawa K. (2006). Bingding free energy calculation and structural analysis for antigen-antibody complex. AIP Conf. Proc..

[51] Wang Z., Wei P., Wang L., Wang Q. (2012). Design, synthesis, and anti-tobacco mosaic virus (TMV) activity of phenanthroindolizidines and their analogues. J. Agric. Food Chem..

[52] Gooding G.V., Hebert T.T. (1967). A simple technique for purification of tobacco mosaic virus in large quantities. Phytopathology.

[53] Li S.Z., Wang D.M., Jiao S.M., Li S.Z. (1991). Pesticide Experiment Methods-Fungicide Sector.

