# Supplementary material for: Discovery of Flavone Derivatives Containing Carboxamide Fragments as Novel Antiviral Agents
Source: Molecules. 2023 Feb 26;28(5):2179. doi: 10.3390/molecules28052179 (PMC10004232; doi:10.3390/molecules28052179)
Supplement: Supplementary file 1 [file molecules-28-02179-s001.zip › molecules-2224281-supplementary.pdf]

molecules

MDPI

Supplementary Materials

Discovery of Flavone Derivatives Containing Carboxamide Fragments as Novel Antiviral Agents

Bobo Zhao [1,†], Jiali Wang [1,†], Lu Wang [1], Ziwen Wang [2,*] and Aidang Lu [1,*]

1. School of Chemical Engineering and Technology, Hebei University of Technology, Tianjin 300401, China
2. Tianjin Key Laboratory of Structure and Performance for Functional Molecules, College of Chemistry, Tianjin Normal University, Tianjin 300387, China

* Correspondence: hxxywzw@tjnu.edu.cn (Z.W.); luaidang@hebut.edu.cn (A.L.); Tel.: +86-22-23766531 (Z.W.); +86-22-60202812 (A.L.)

† These authors contributed equally to this work.

Contents

Section S1: Phytotoxic Activity 3

Section S2: Copies of NMR spectra (Figures S1–S60) 4

Figures S1–S2. ^{1}H NMR and ^{13}C NMR spectra of **2a** 4

Figures S3–S4. ^{1}H NMR and ^{13}C NMR spectra of **2b** 5

Figures S5–S6. ^{1}H NMR and ^{13}C NMR spectra of **2c** 6

Figures S7–S8. ^{1}H NMR and ^{13}C NMR spectra of **2d** 7

Figures S9–S10. ^{1}H NMR and ^{13}C NMR spectra of **2e** 8

Figures S11–S12. ^{1}H NMR and ^{13}C NMR spectra of **2f** 9

Figures S13–S14. ^{1}H NMR and ^{13}C NMR spectra of **3** 10

Figures S15–S16. ^{1}H NMR and ^{13}C NMR spectra of **4a** 11

Figures S17–S18. ^{1}H NMR and ^{13}C NMR spectra of **4b** 12

Figures S19–S20. ^{1}H NMR and ^{13}C NMR spectra of **4c** 13

Figures S21–S22. ^{1}H NMR and ^{13}C NMR spectra of **4d** 14

Figures S23–S24. ^{1}H NMR and ^{13}C NMR spectra of **4e** 15

Figures S25–S26. ^{1}H NMR and ^{13}C NMR spectra of **4f** 16

Figures S27–S28. ^{1}H NMR and ^{13}C NMR spectra of **4g** 18

Figures S29–S30. ^{1}H NMR and ^{13}C NMR spectra of **4h** 18

Figures S31–S32. ^{1}H NMR and ^{13}C NMR spectra of **4i** 19

Figures S33–S34. ^{1}H NMR and ^{13}C NMR spectra of **4j** 20

Figures S35–S36. ^{1}H NMR and ^{13}C NMR spectra of **4k** 21

Figures S37–S38. ^{1}H NMR and ^{13}C NMR spectra of **4l** 22

Figures S39–S40. ^{1}H NMR and ^{13}C NMR spectra of **4m** 23

Figures S41–S42. ^{1}H NMR and ^{13}C NMR spectra of **4n** 24

Figures S43–S44. ^{1}H NMR and ^{13}C NMR spectra of **5a** 25

Figures S45–S46. ^{1}H NMR and ^{13}C NMR spectra of **5b** 26

Figures S47–S48. ^{1}H NMR and ^{13}C NMR spectra of **5c** 27

Figures S49–S50. ^{1}H NMR and ^{13}C NMR spectra of **5d** 28

Figures S51–S52. ^{1}H NMR and ^{13}C NMR spectra of **5e** 29

Figures S53–S54. ^{1}H NMR and ^{13}C NMR spectra of **6a** 30

Figures S55–S56. ^{1}H NMR and ^{13}C NMR spectra of **6b** 31

Figures S57–S58. ^{1}H NMR and ^{13}C NMR spectra of **6c** 32

Figures S59–S60. ^{1}H NMR and ^{13}C NMR spectra of **6d** 33

Section S3: Molecule docking results of 1 and 2a with TMV CP (Figure S61). 34

Section S1. Phytotoxic Activity

The growing 5–6 leaf stage tobaccos (*Nicotiana tabacum var Xanthi nc*) were selected. The compound solution (500 μg/mL) was smeared on the leaves and calculated the number of lesions after 0, 3, 7 and 10 days respectively. [7,51] There are three replicates for each compound.

Section S3. Copies of NMR spectra (Figures S1–S60)

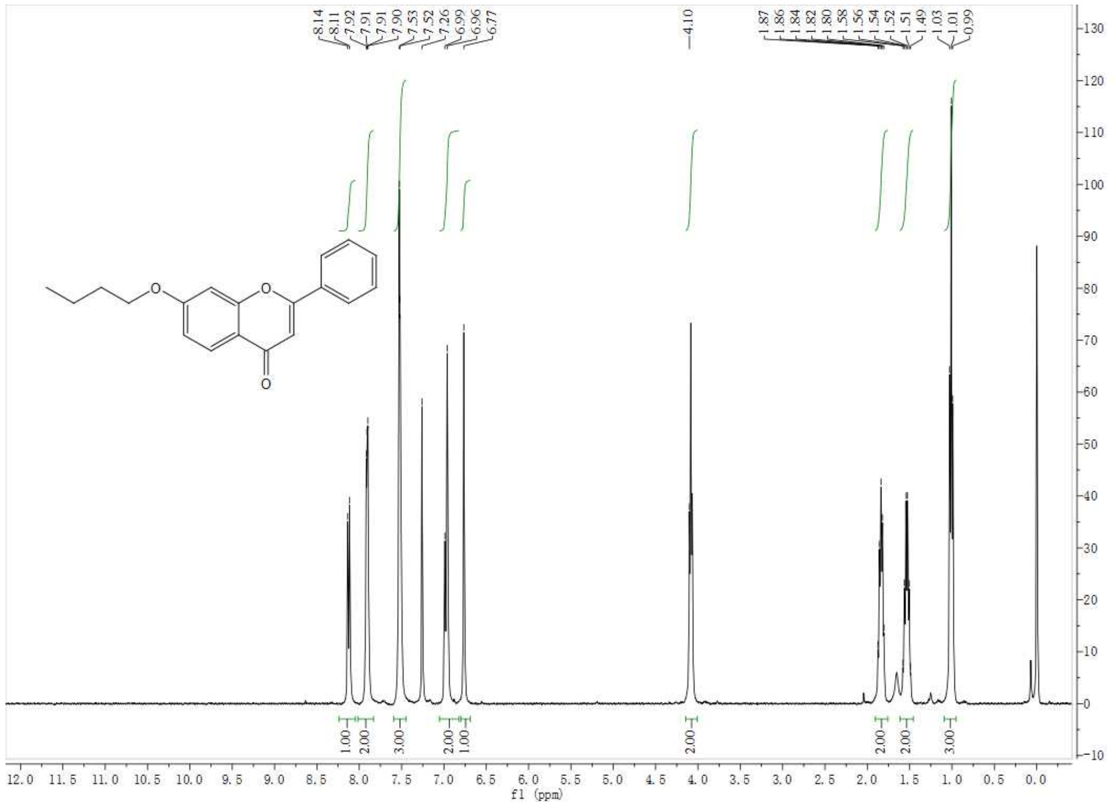

Figure S1. ^{1}H NMR spectrum of **2a**

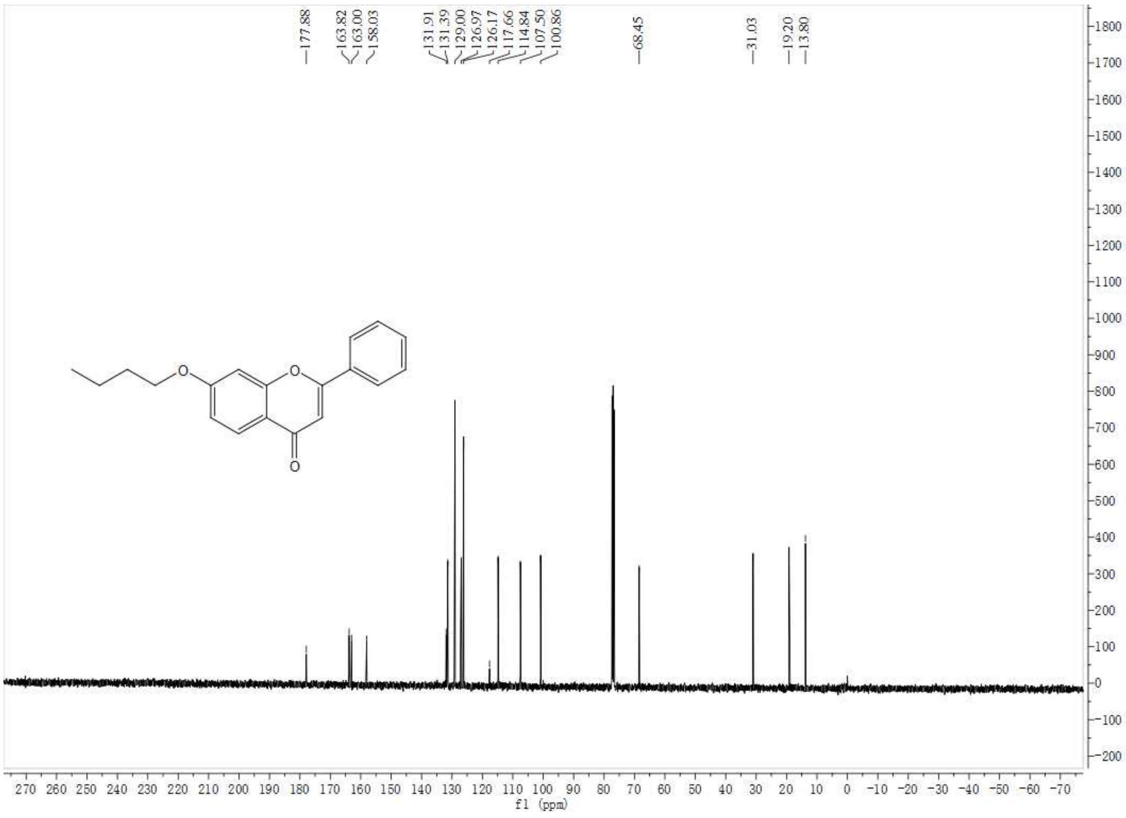

Figure S2. ^{13}C NMR spectrum of **2a**

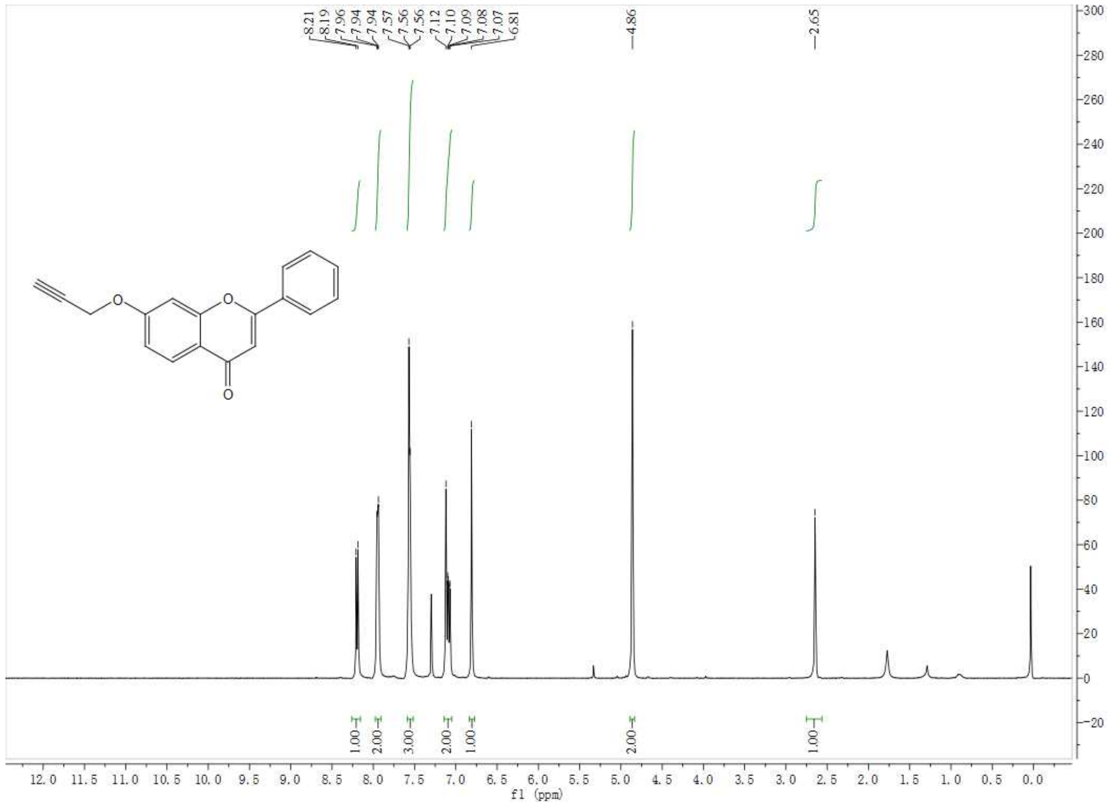

Figure S3. ^{1}H NMR spectrum of **2b**

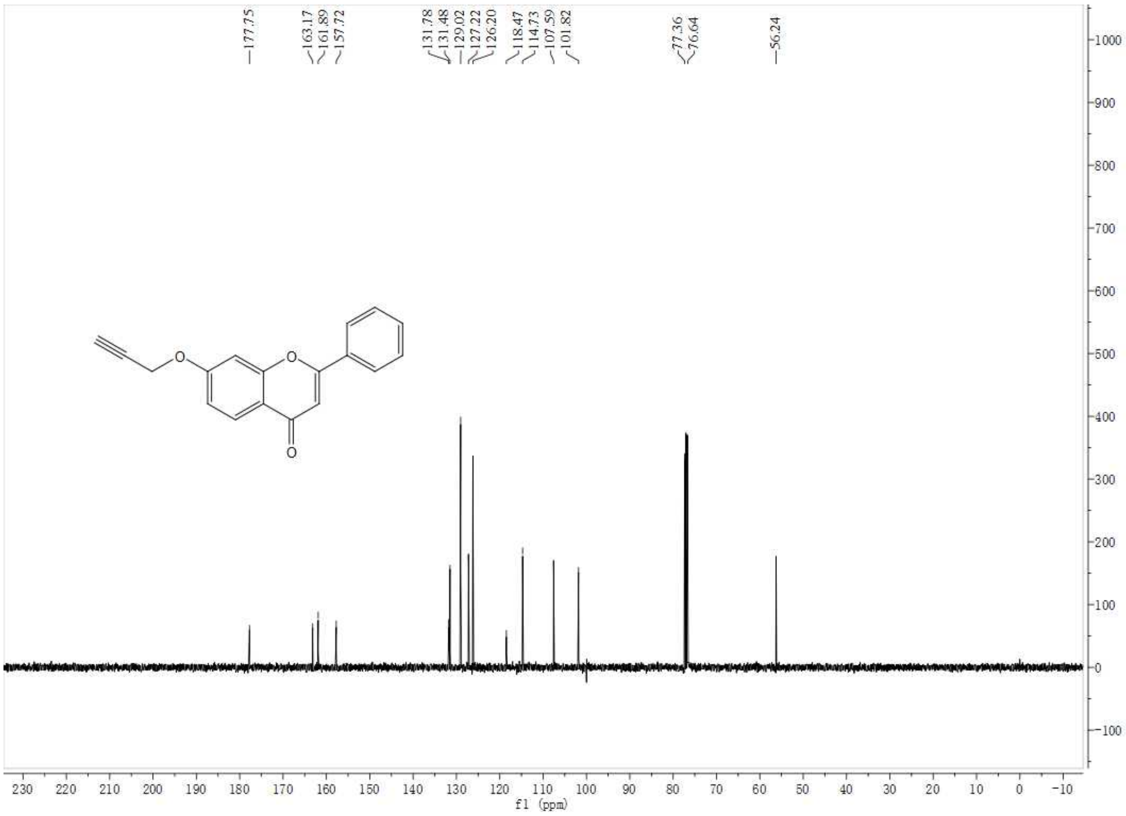

Figure S4. ^{13}C NMR spectrum of **2b**

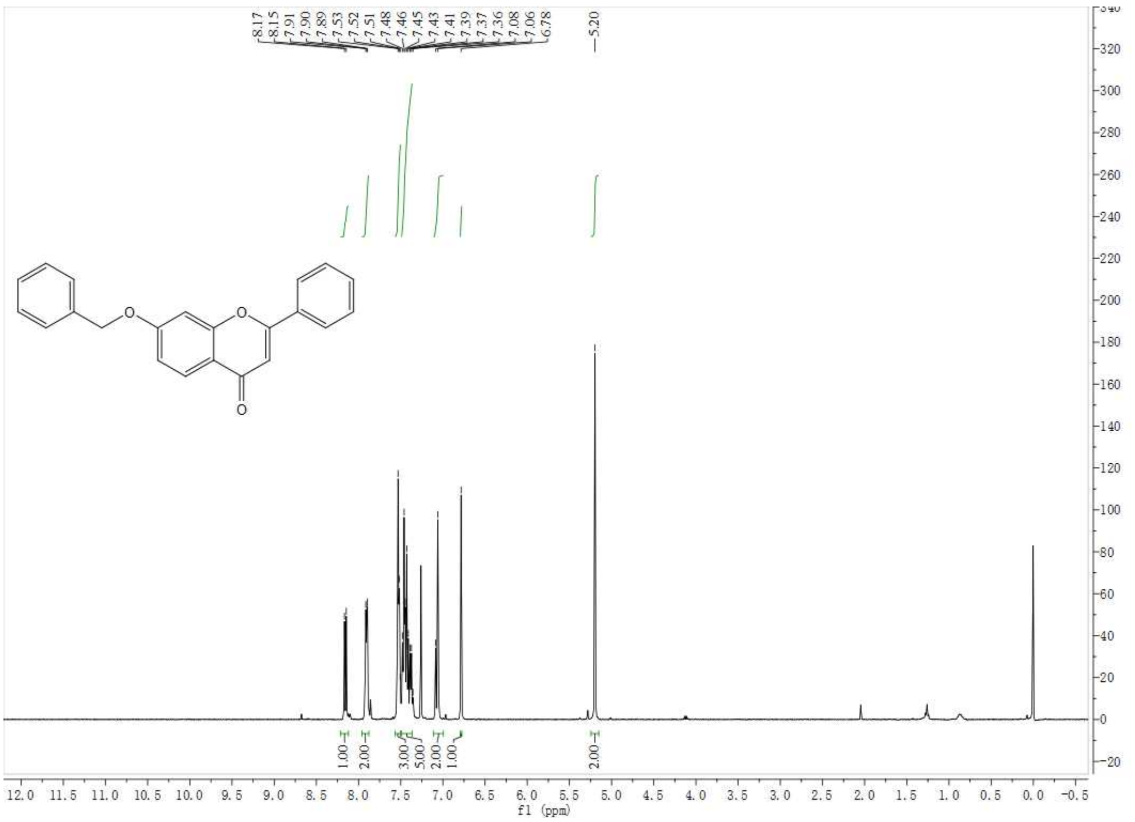

Figure S5. ^{1}H NMR spectrum of **2c**

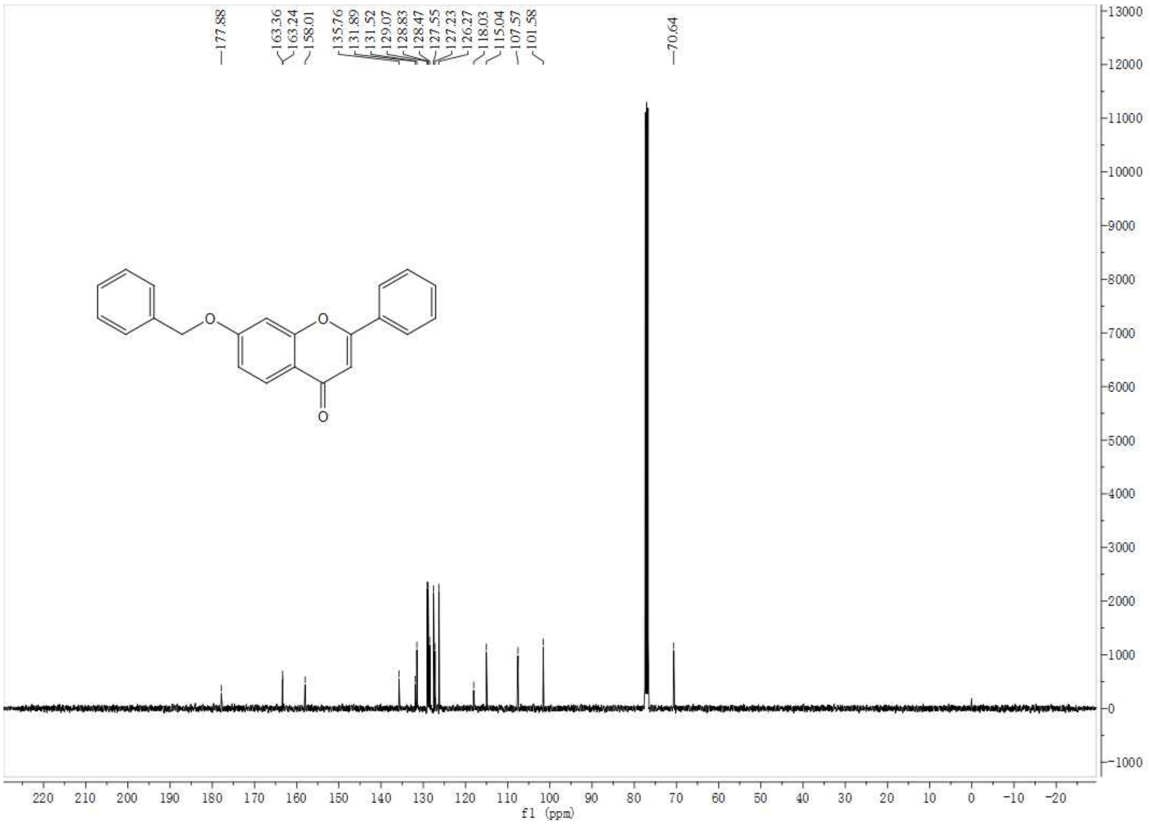

Figure S6. ^{13}C NMR spectrum of **2c**

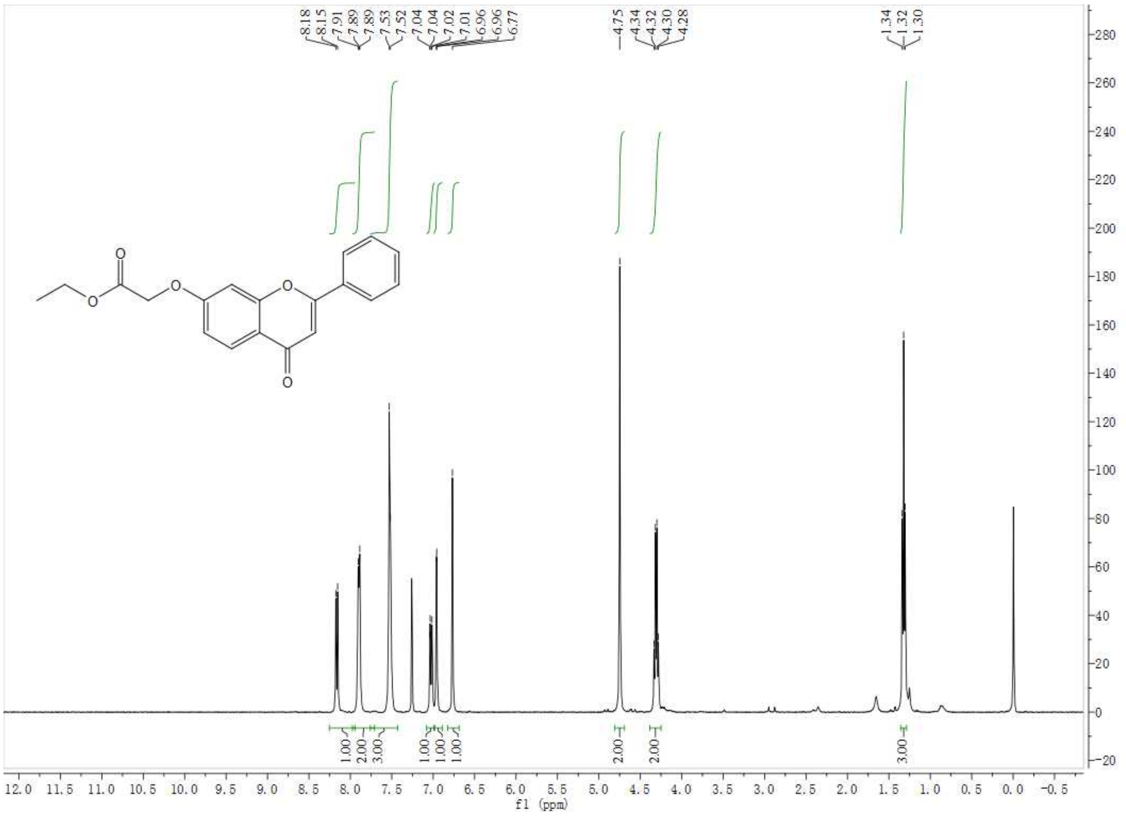

Figure S7. ^{1}H NMR spectrum of **2d**

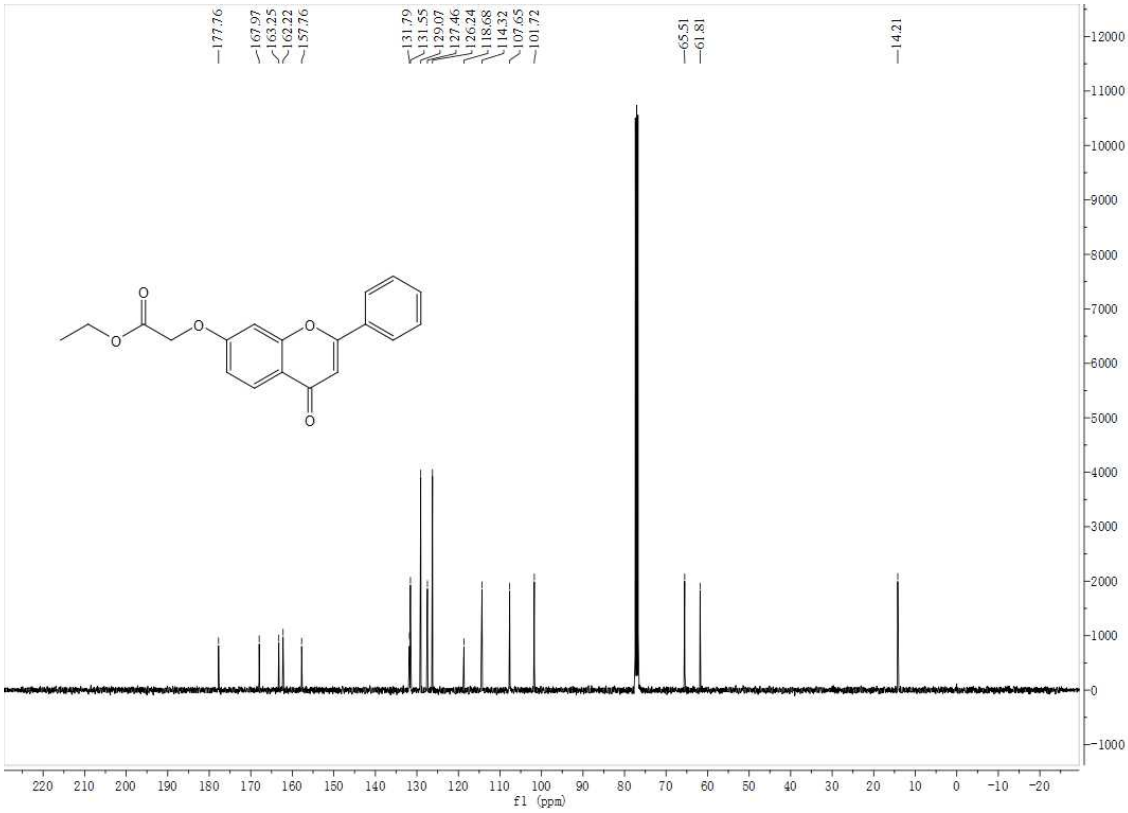

Figure S8. ^{13}C NMR spectrum of **2d**

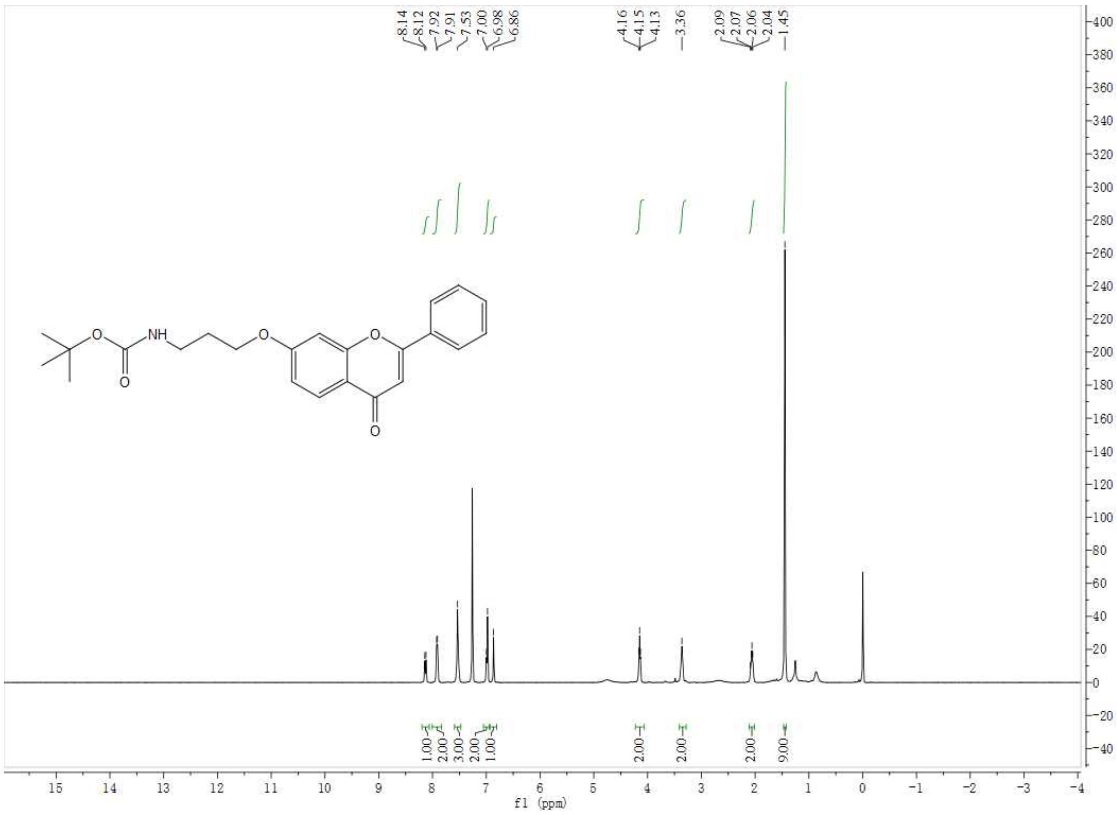

Figure S9. ^{1}H NMR spectrum of **2e**

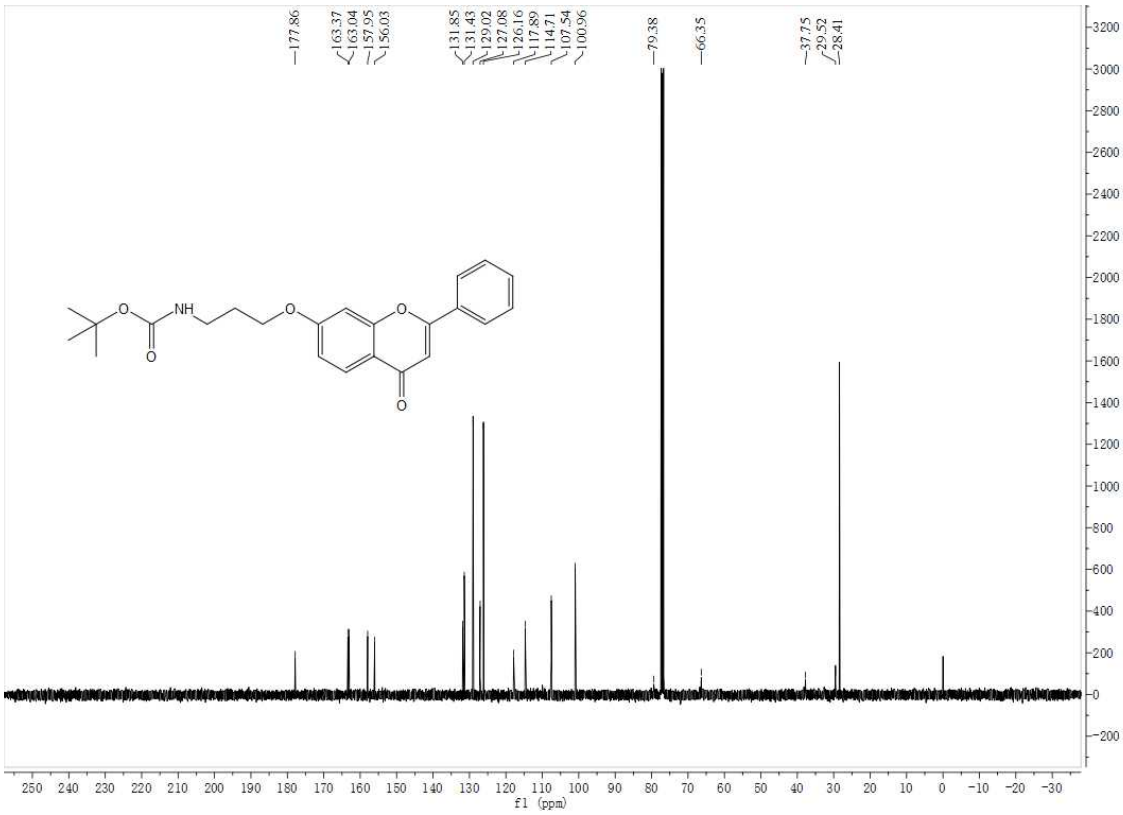

Figure S10. ^{13}C NMR spectrum of **2e**

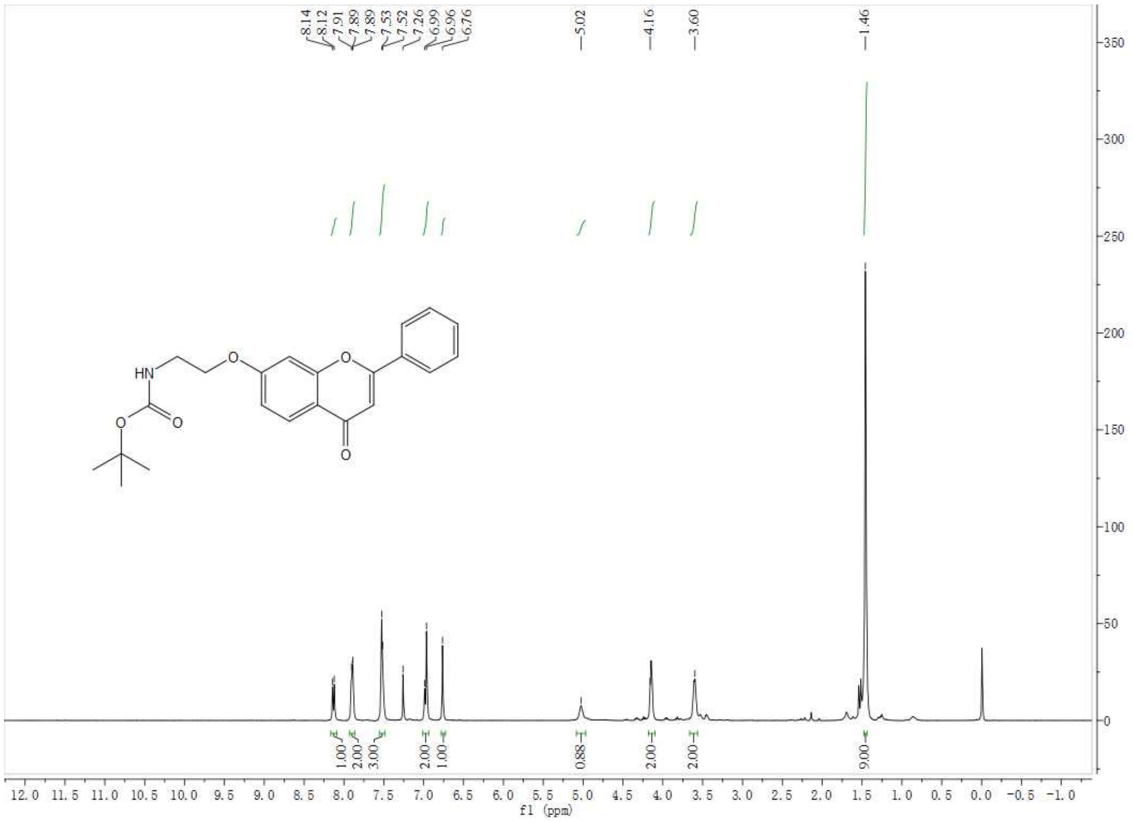

Figure S11. ^{1}H NMR spectrum of **2f**

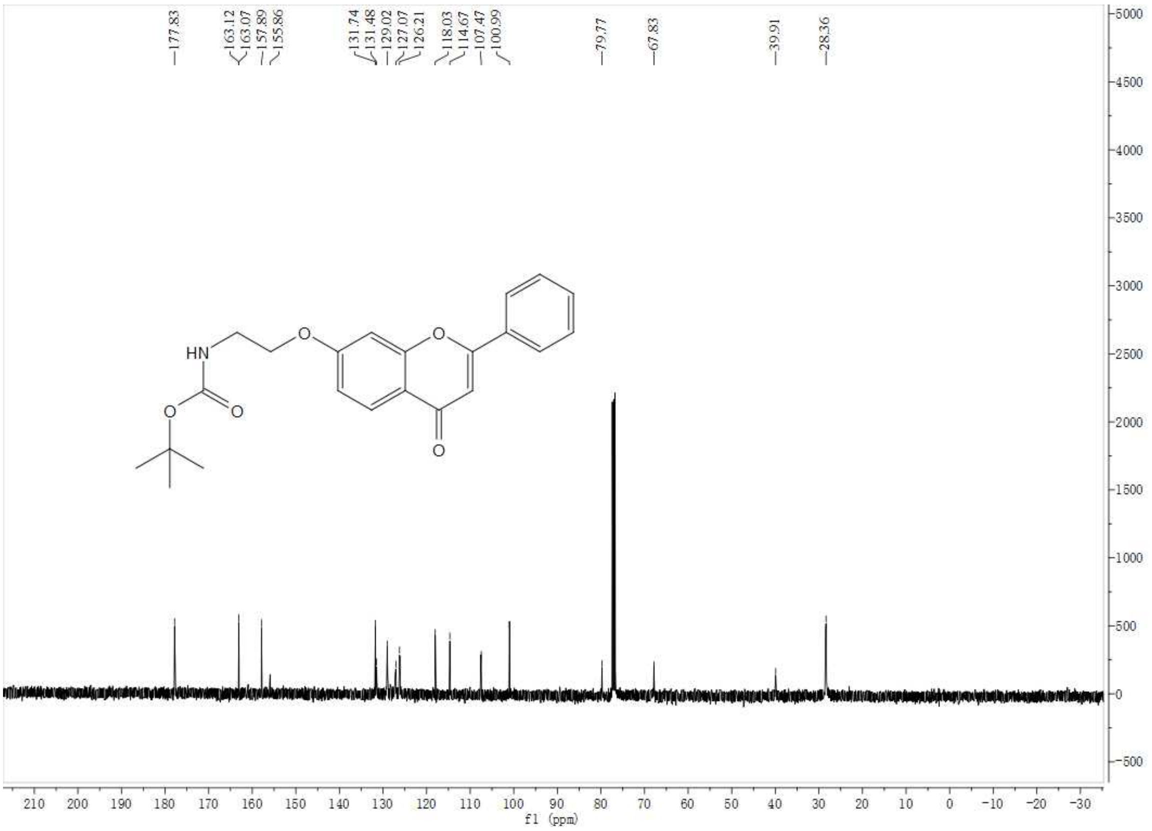

Figure S12. ^{13}C NMR spectrum of **2f**

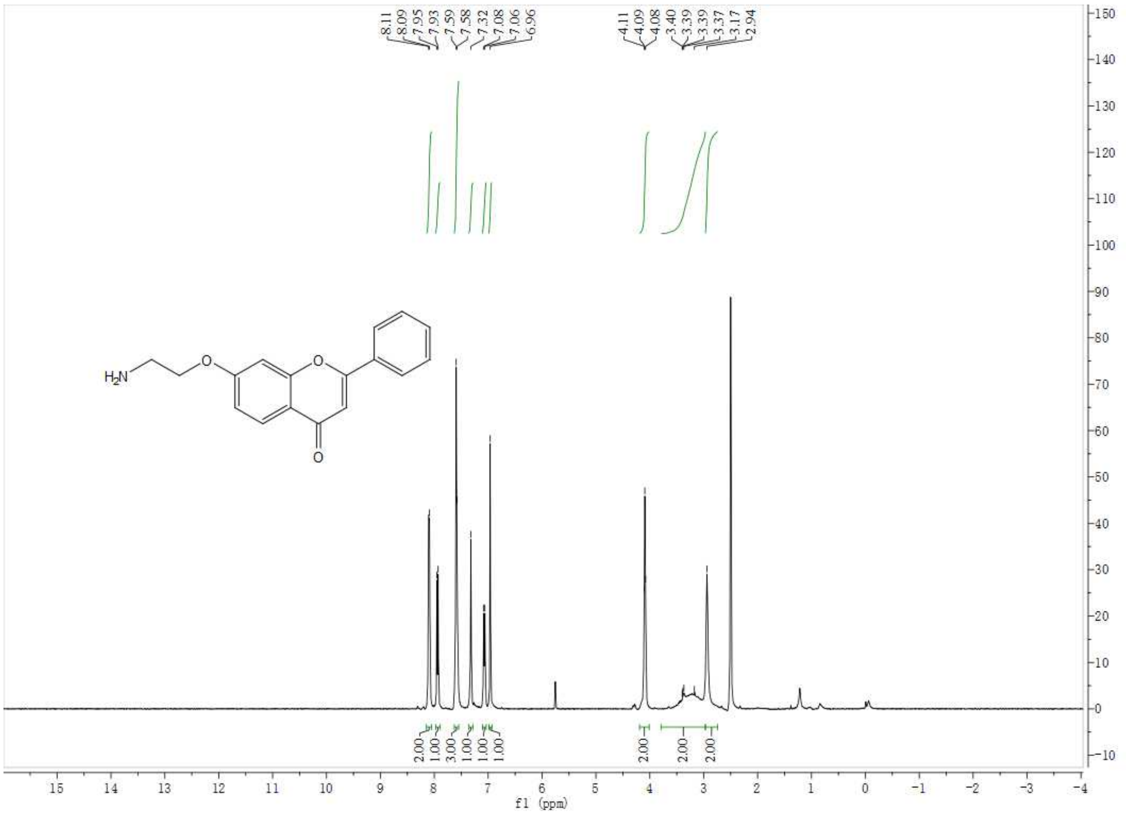

Figure S13. ^{1}H NMR spectrum of **3**

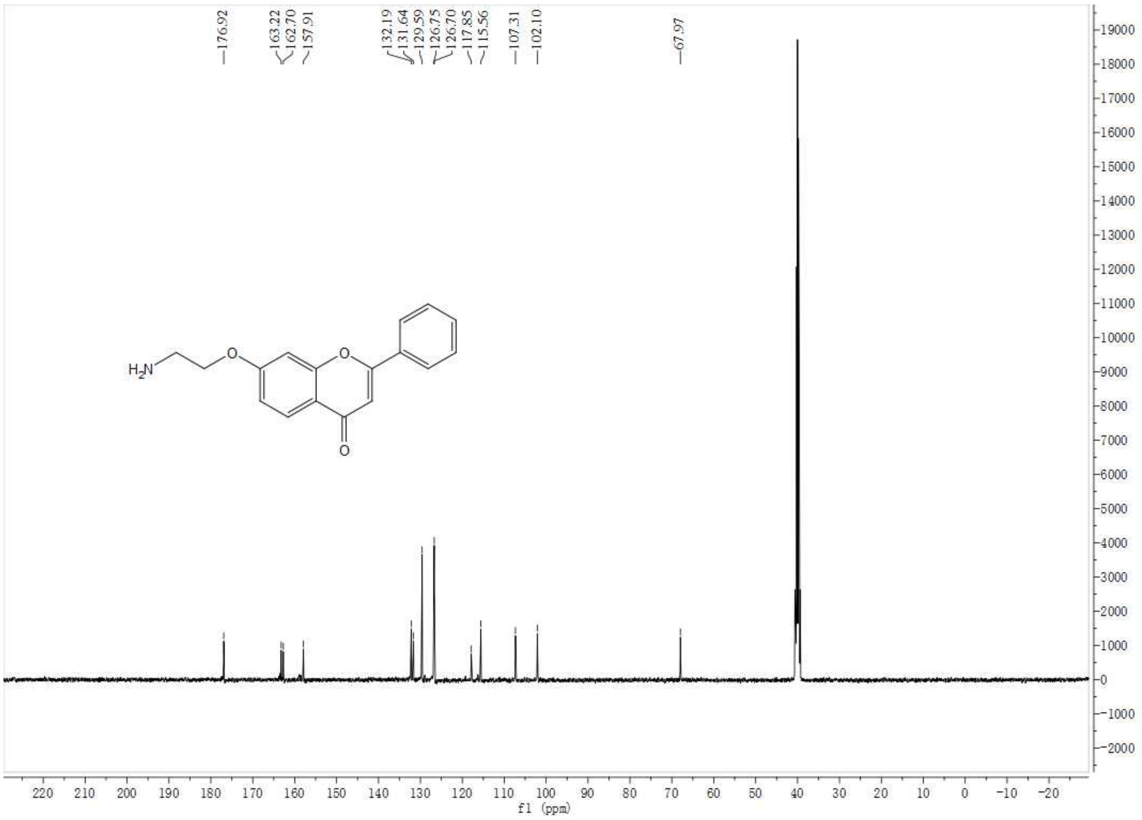

Figure S14. ^{13}C NMR spectrum of **3**

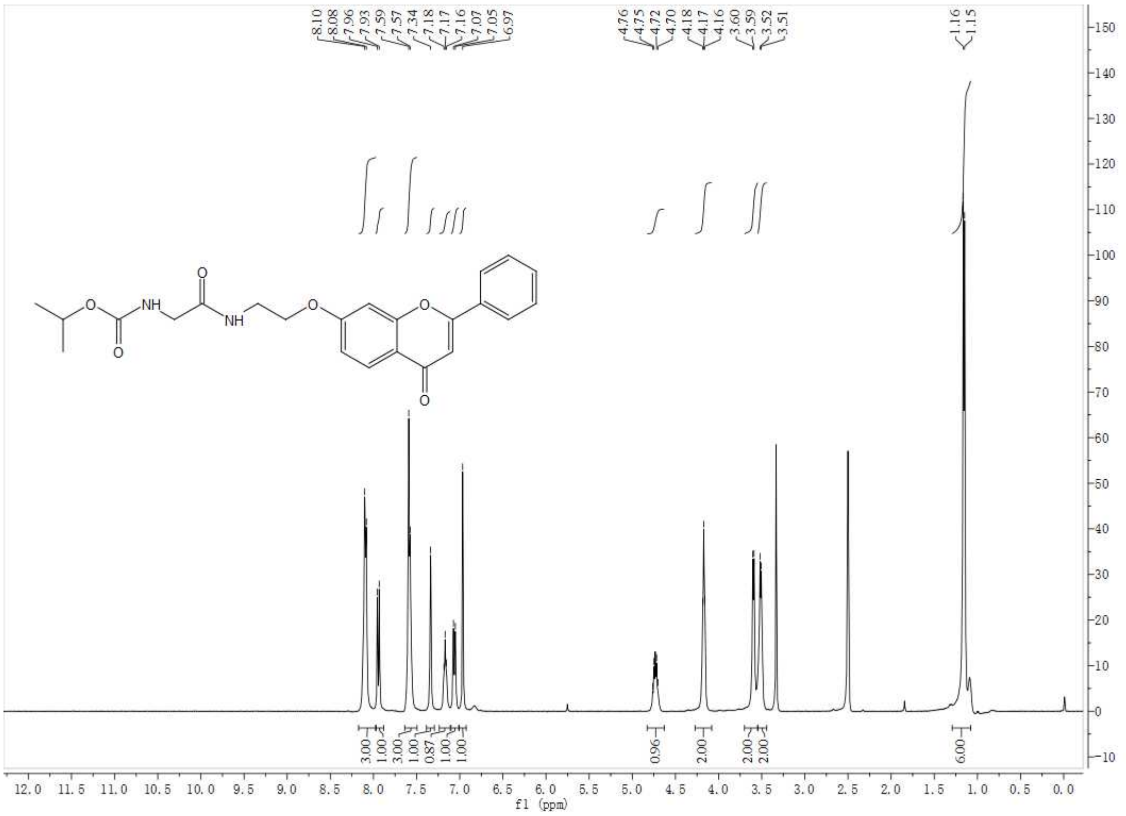

Figure S15. ^{1}H NMR spectrum of **4a**

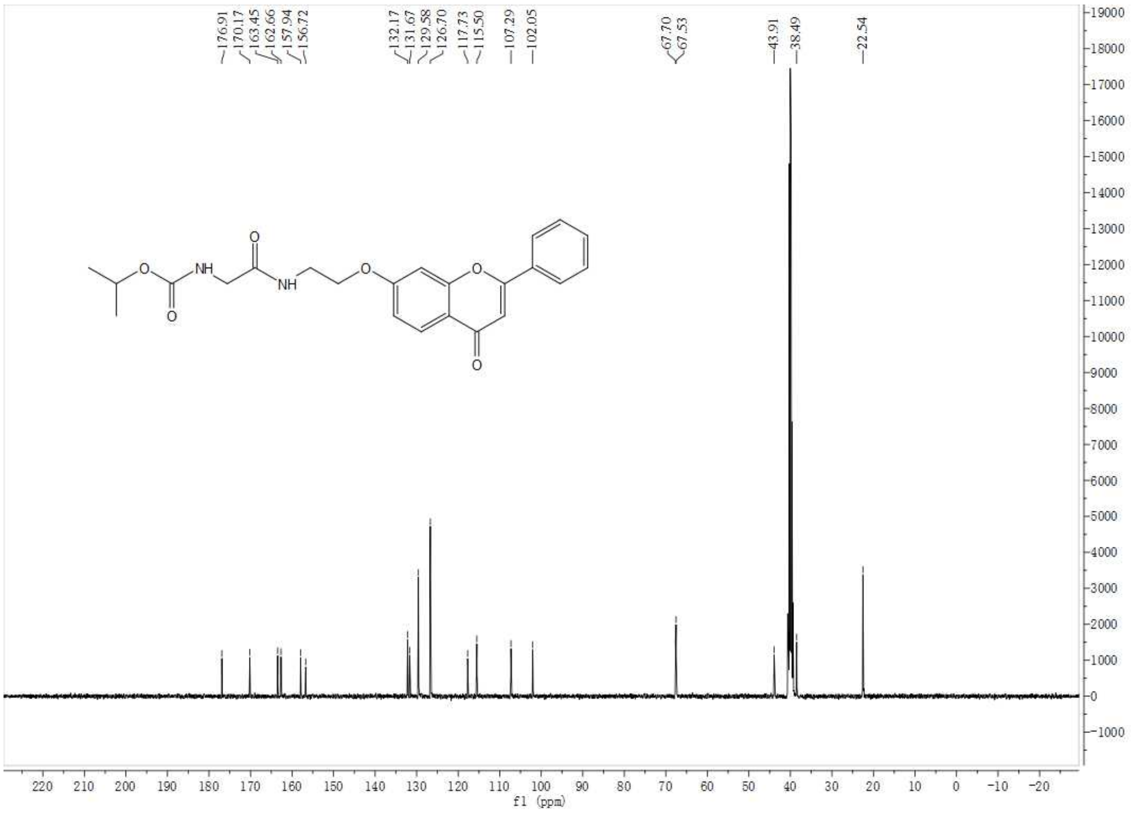

Figure S16. ^{13}C NMR spectrum of **4a**

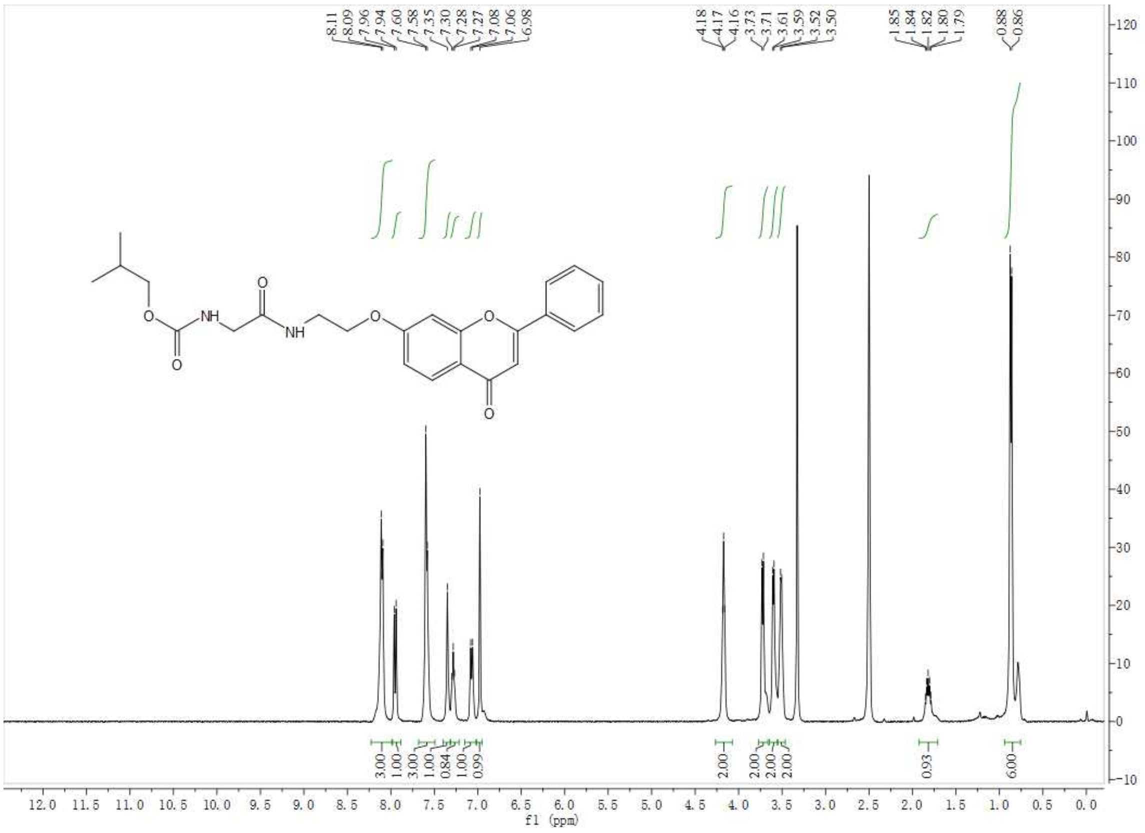

Figure S17. ^{1}H NMR spectrum of **4b**

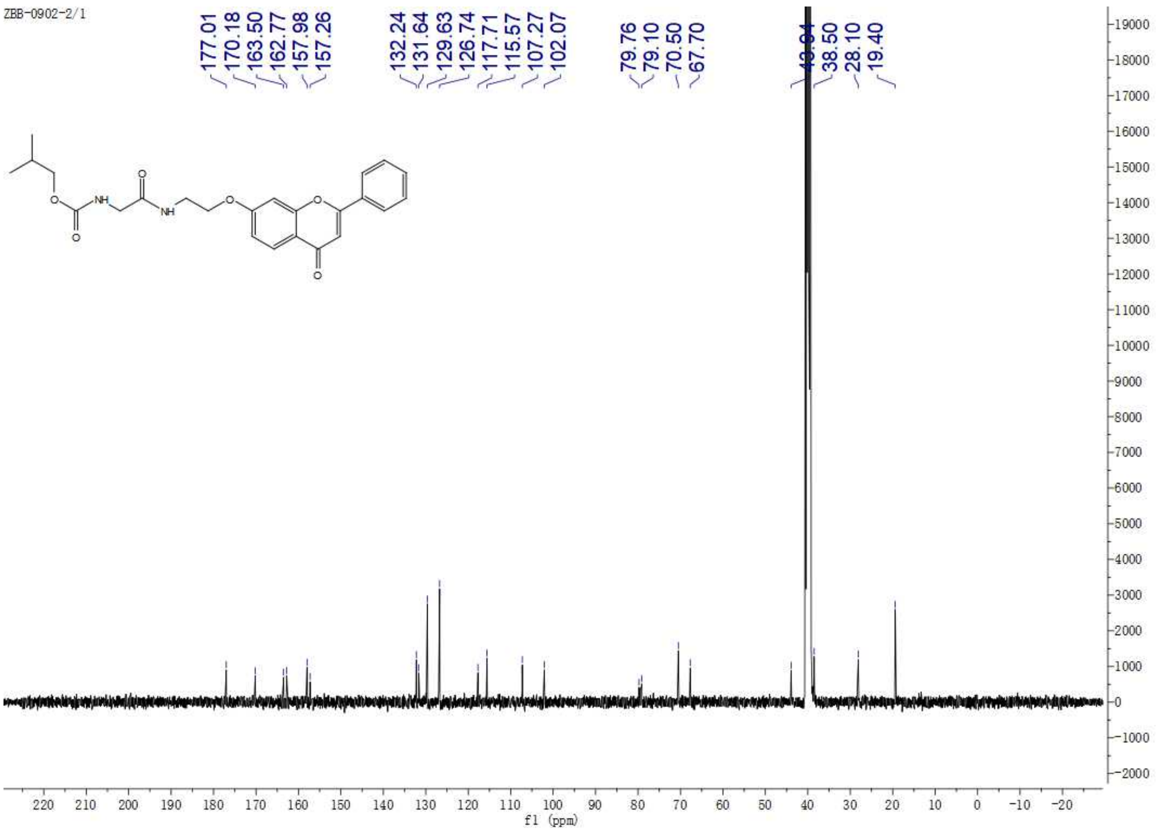

Figure S18. ^{13}C NMR spectrum of **4b**

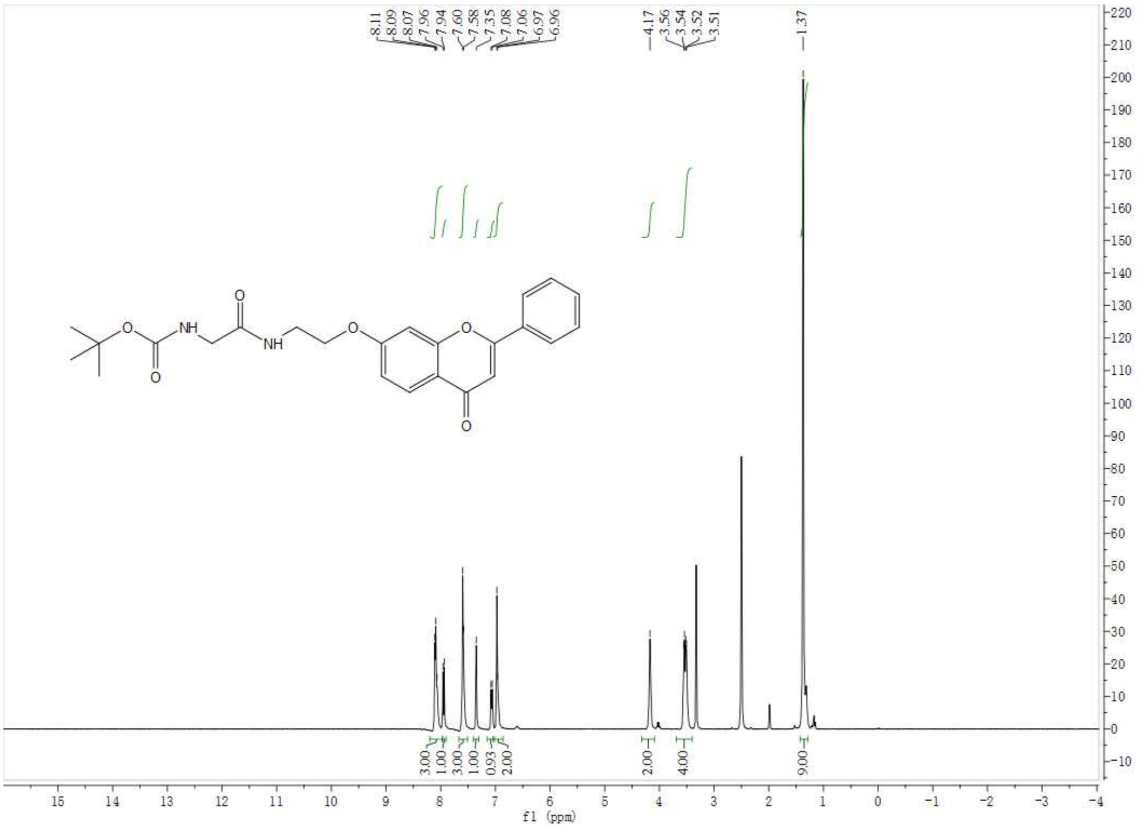

Figure S19. ^{1}H NMR spectrum of **4c**

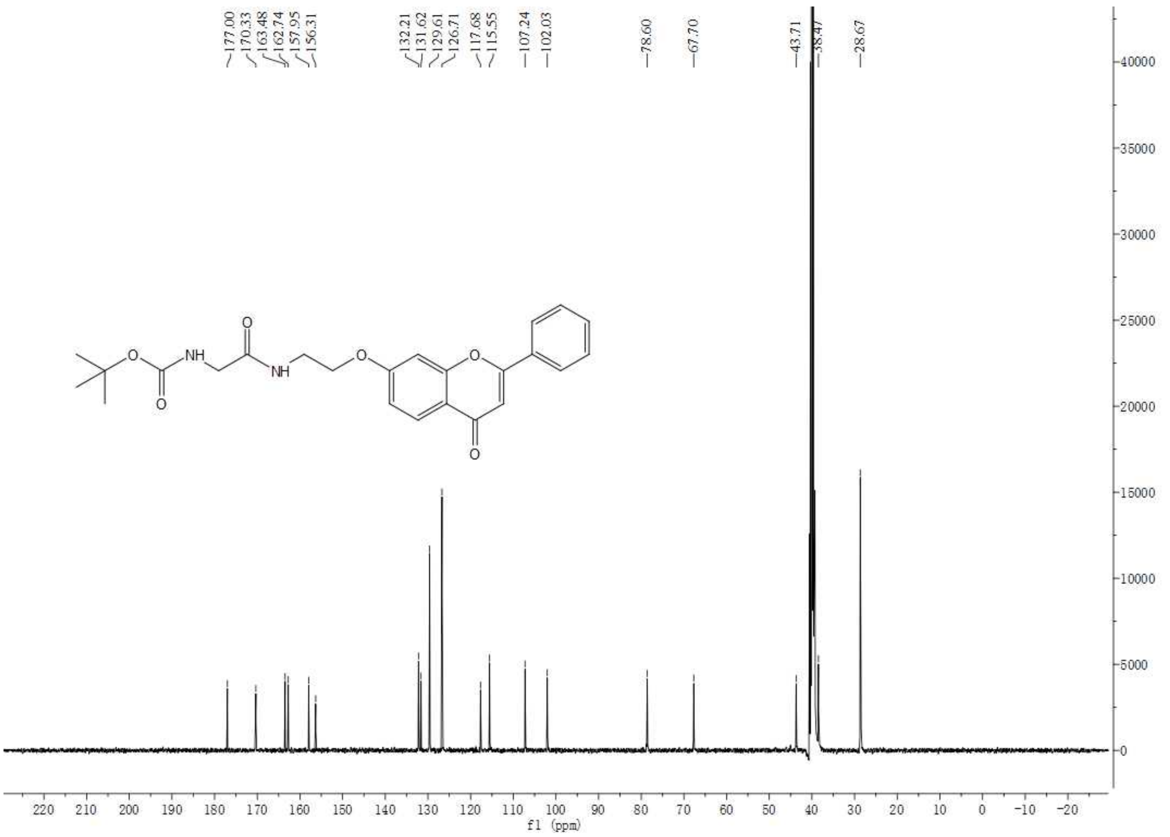

Figure S20. ^{13}C NMR spectrum of **4c**

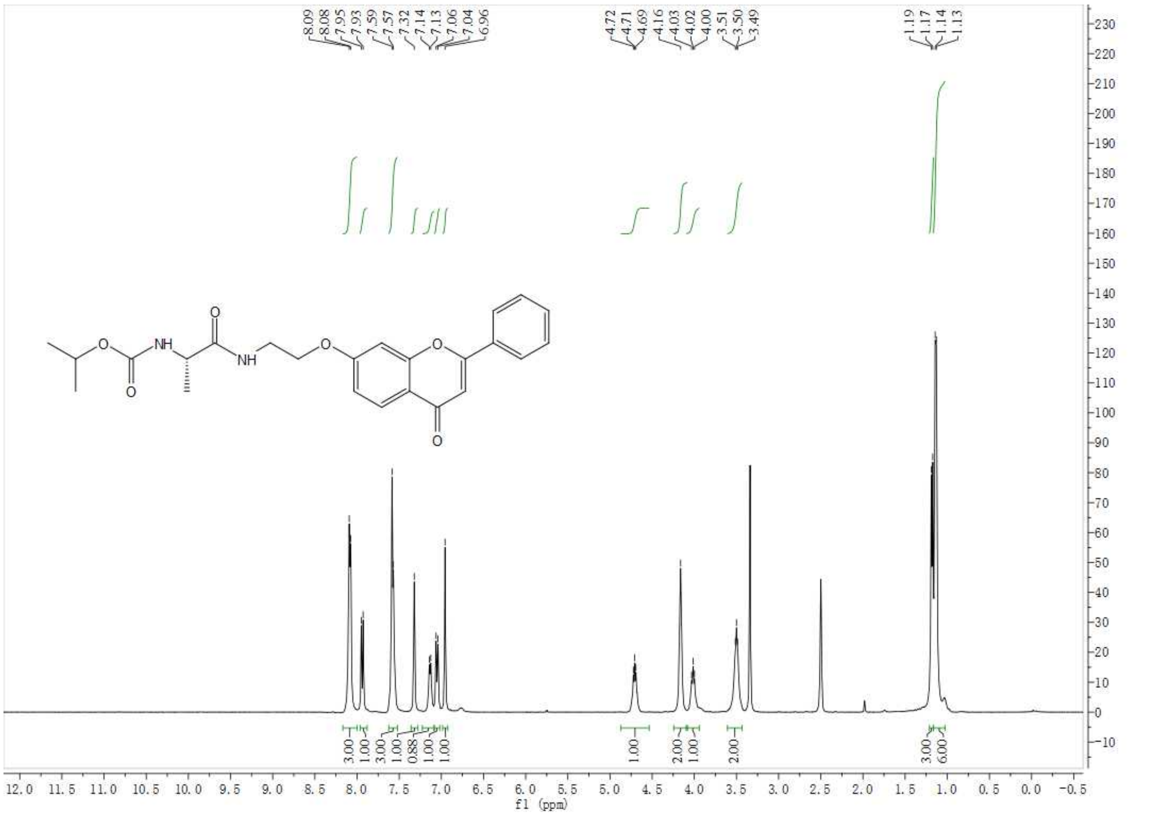

Figure S21. ^{1}H NMR spectrum of **4d**

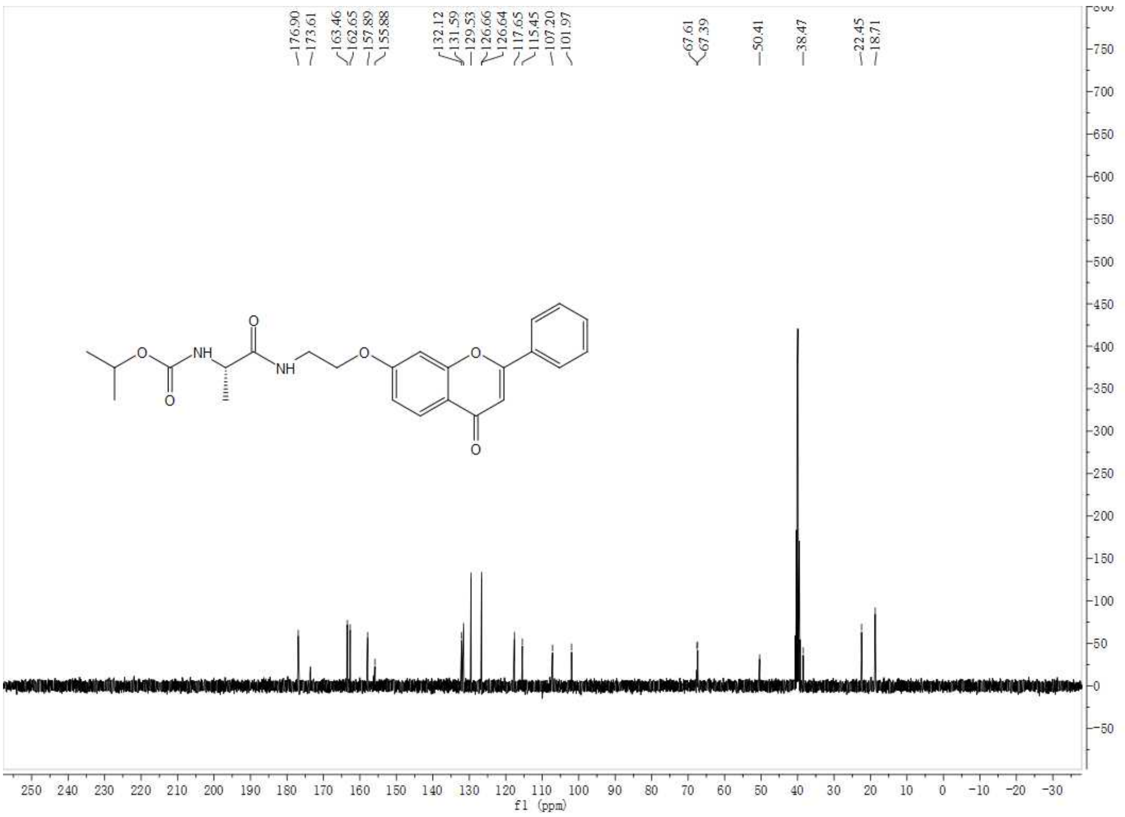

Figure S22. ^{13}C NMR spectrum of **4d**

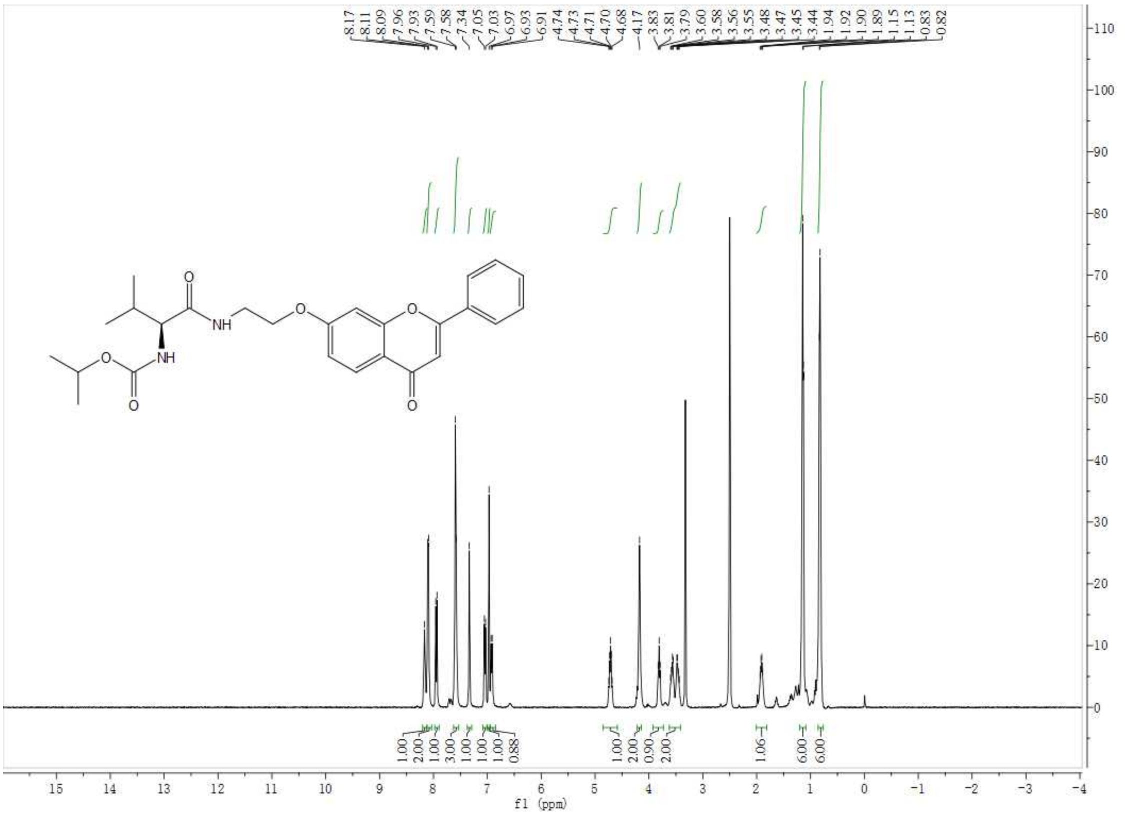

Figure S23. ^{1}H NMR spectrum of **4e**

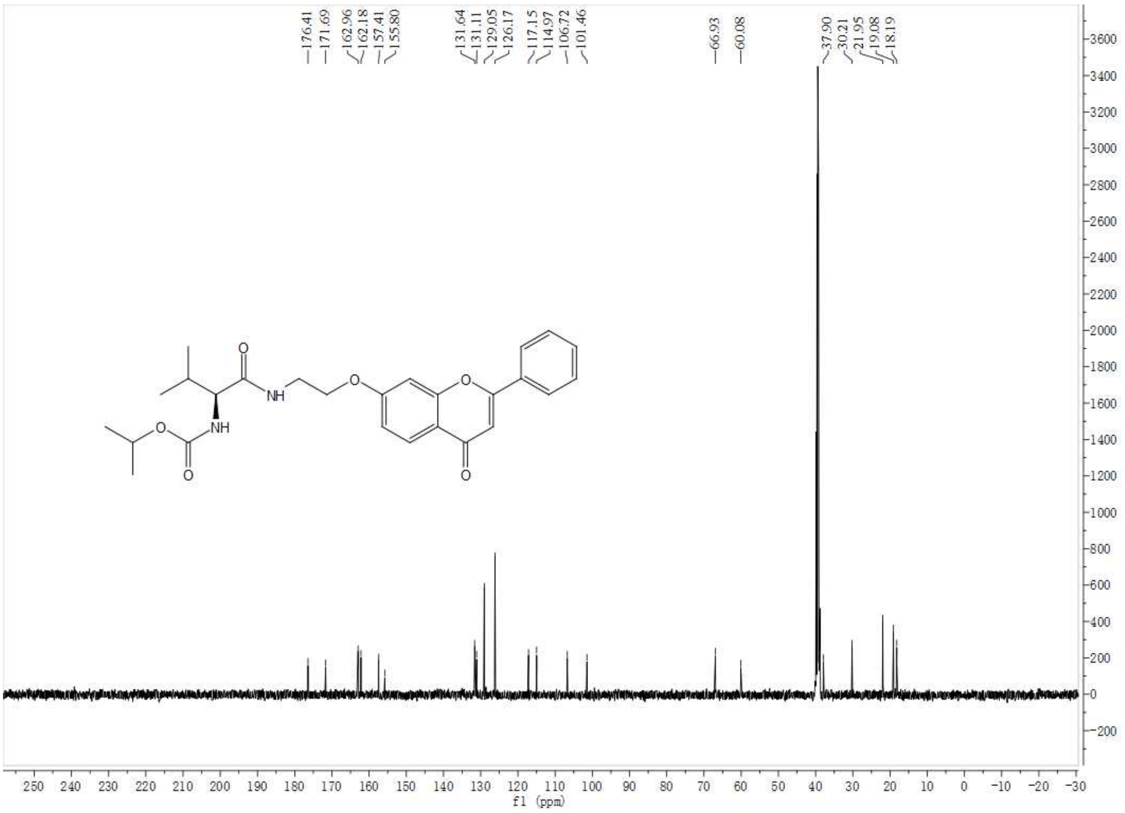

Figure S24. ^{13}C NMR spectrum of **4e**

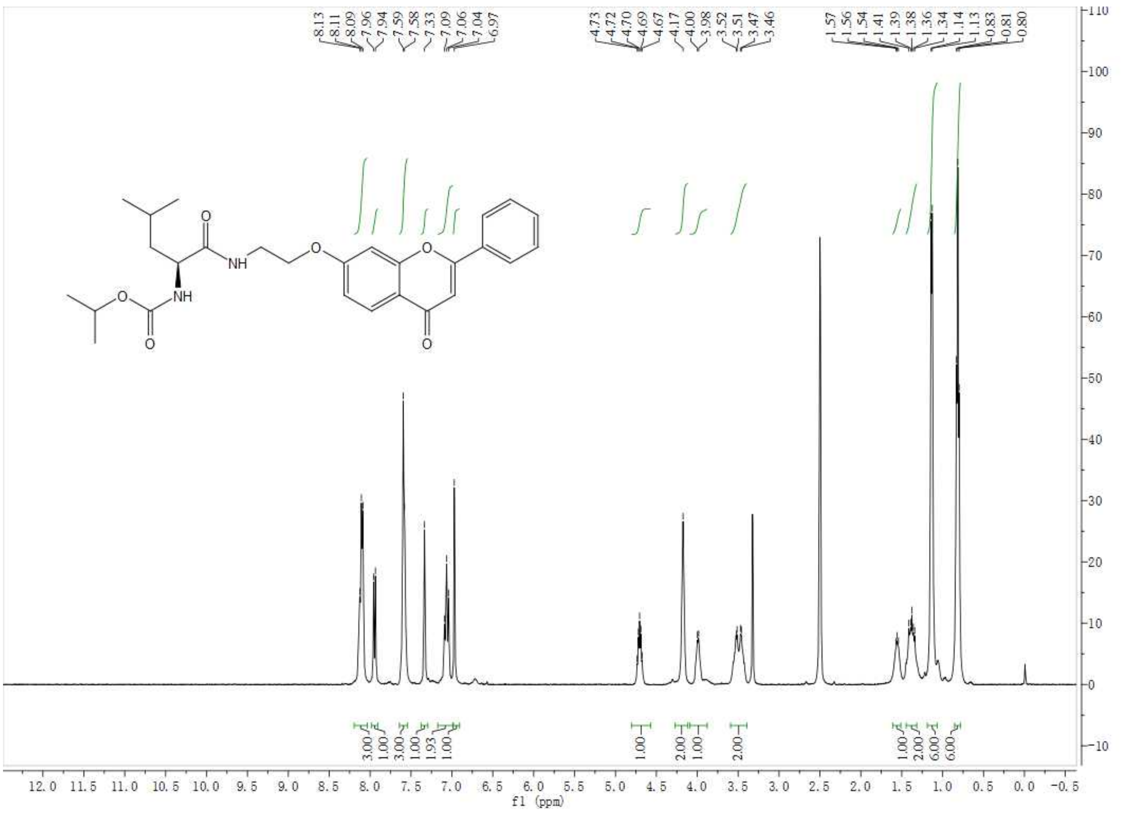

Figure S25. ¹H NMR spectrum of **4f**

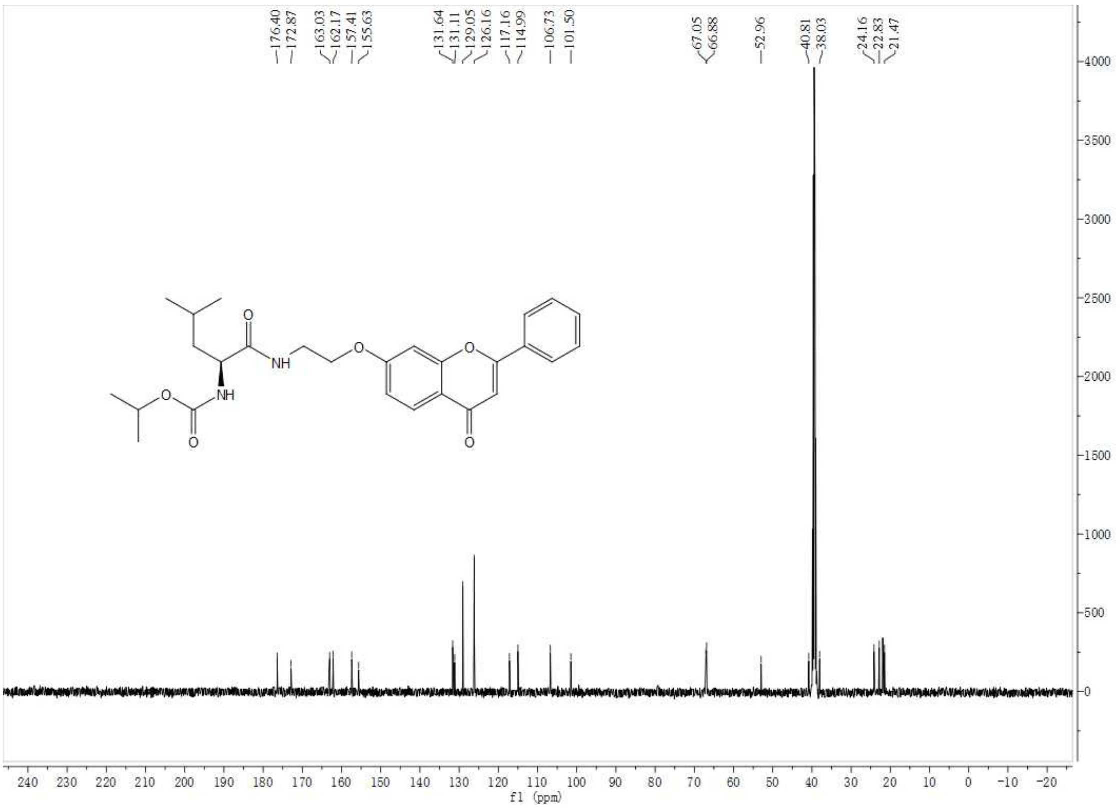

Figure S26. ¹³C NMR spectrum of **4f**

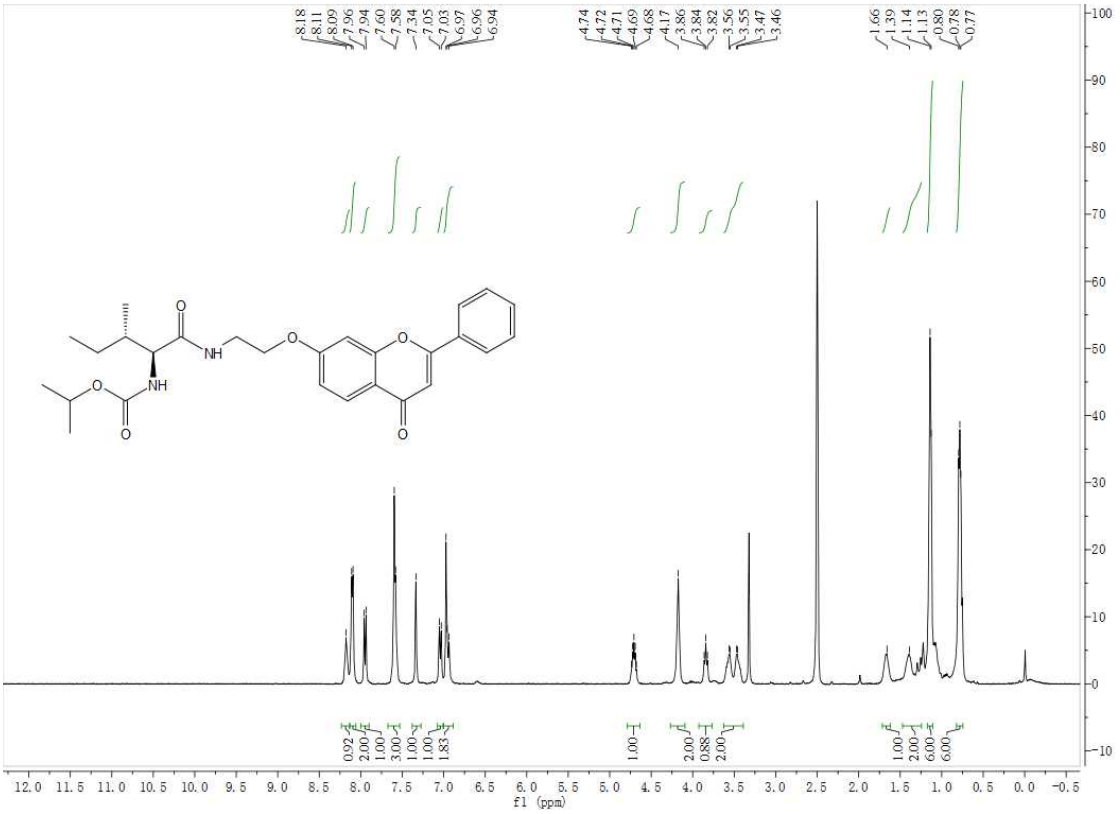

Figure S27. ^{1}H NMR spectrum of **4g**

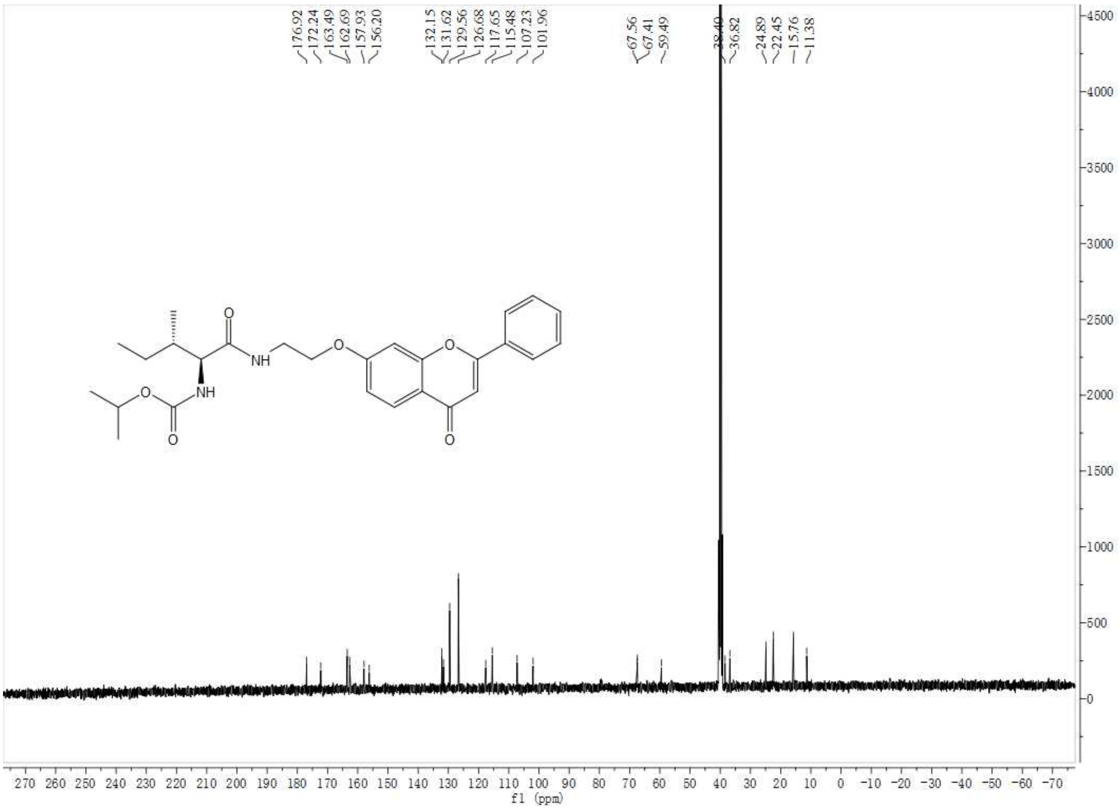

Figure S28. ^{13}C NMR spectrum of **4g**

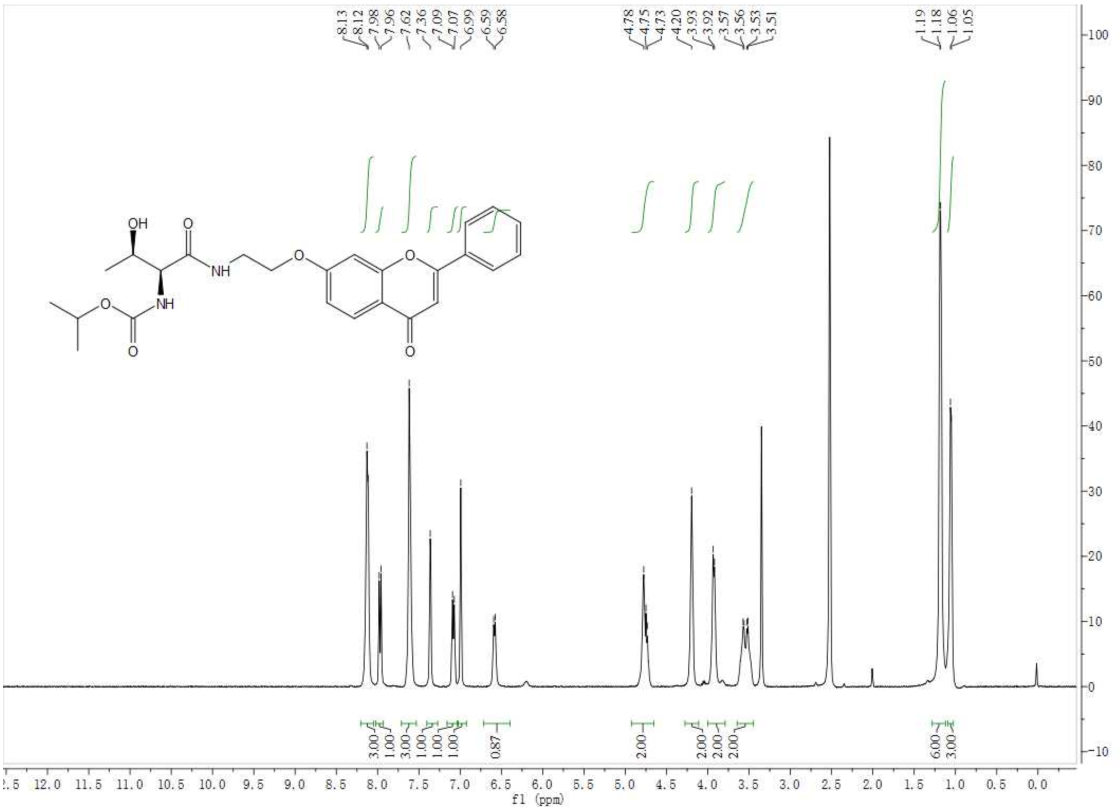

Figure S29. ^{1}H NMR spectrum of **4h**

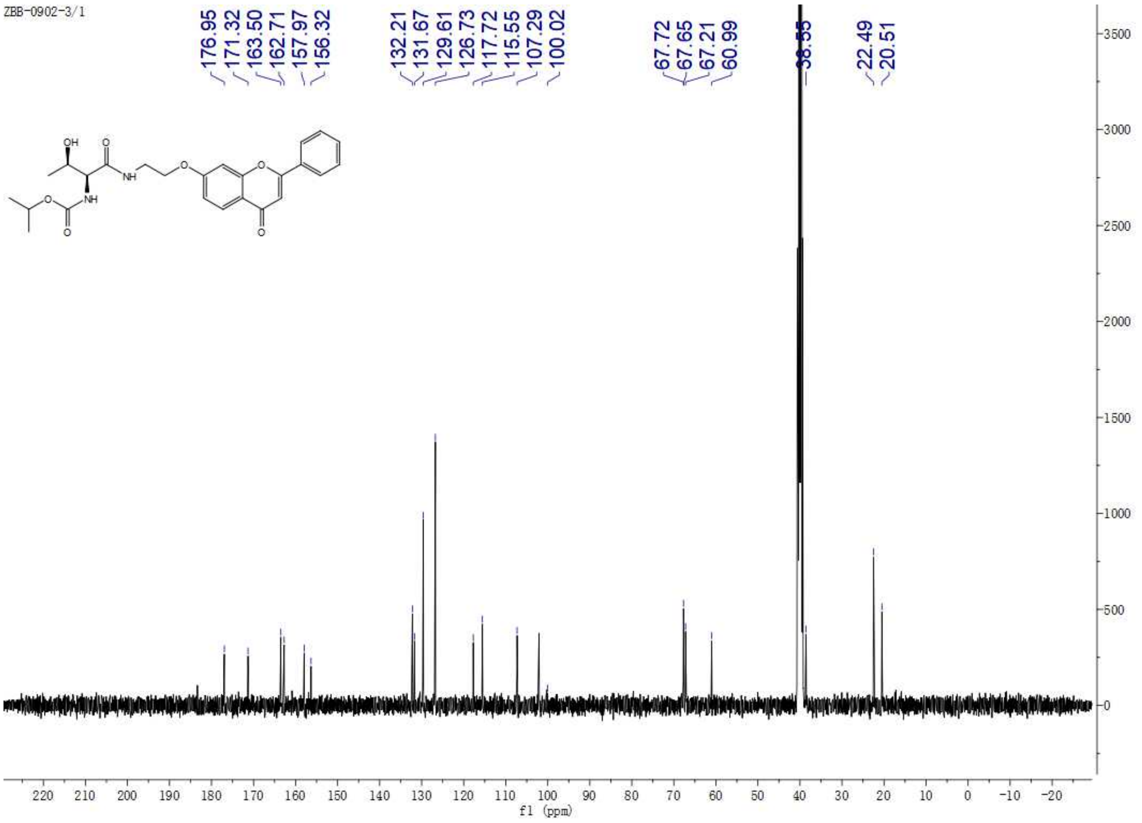

Figure S30. ^{13}C NMR spectrum of **4h**

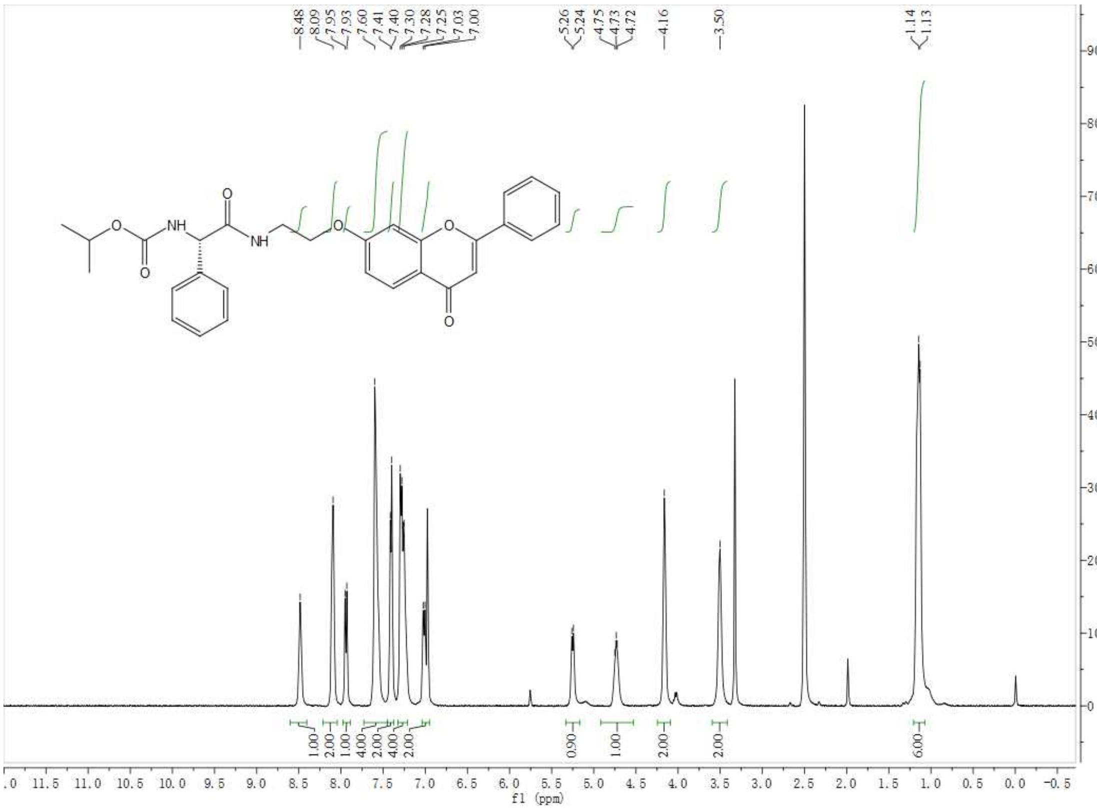

Figure S31. ^{1}H NMR spectrum of **4i**

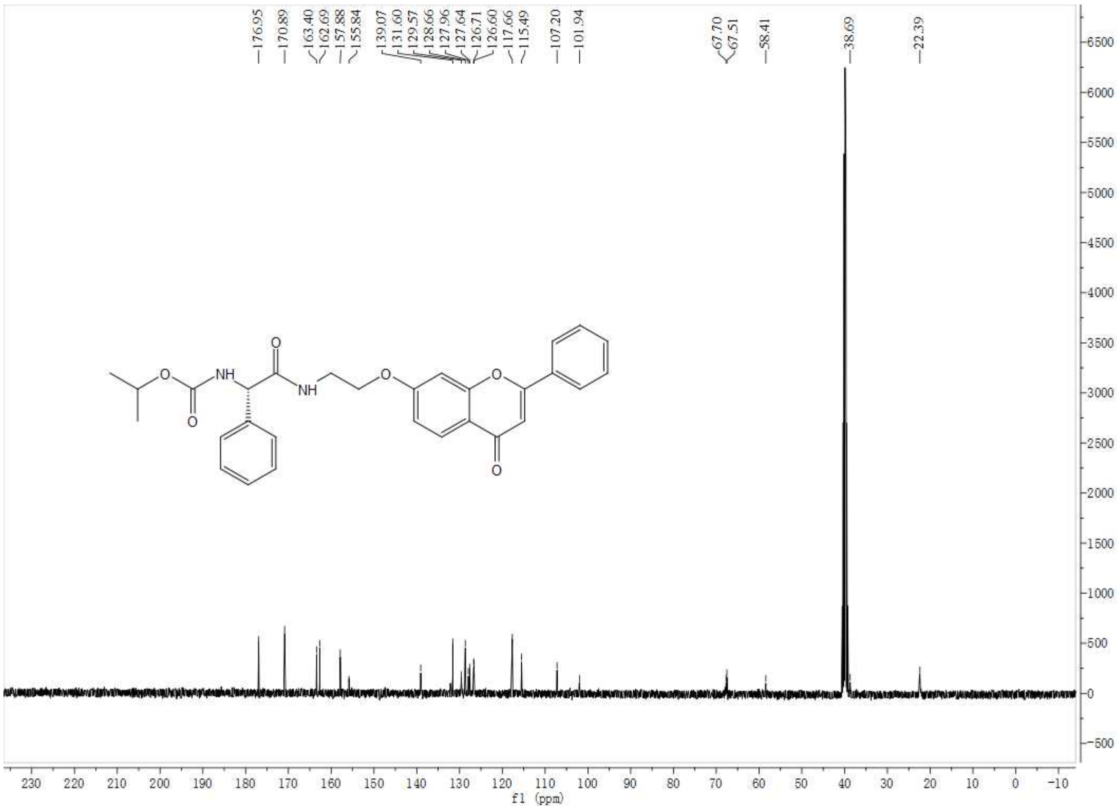

Figure S32. ^{13}C NMR spectrum of **4i**

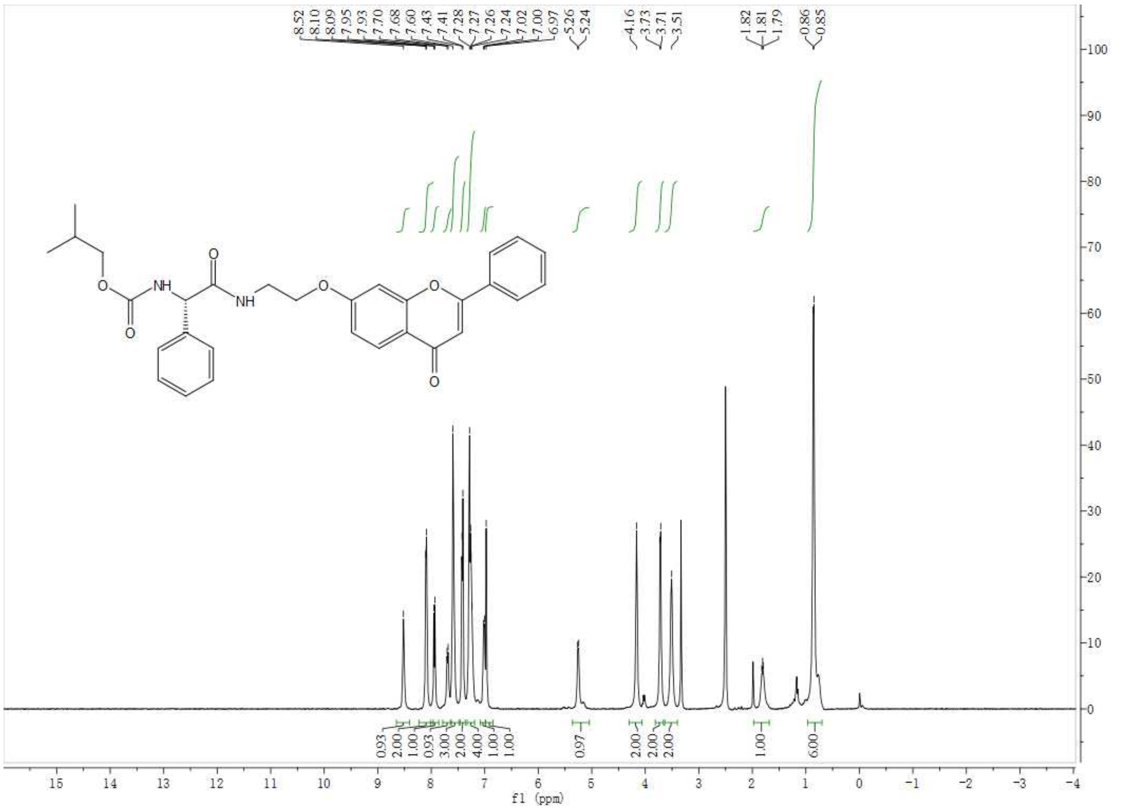

Figure S33. ^{1}H NMR spectrum of **4j**

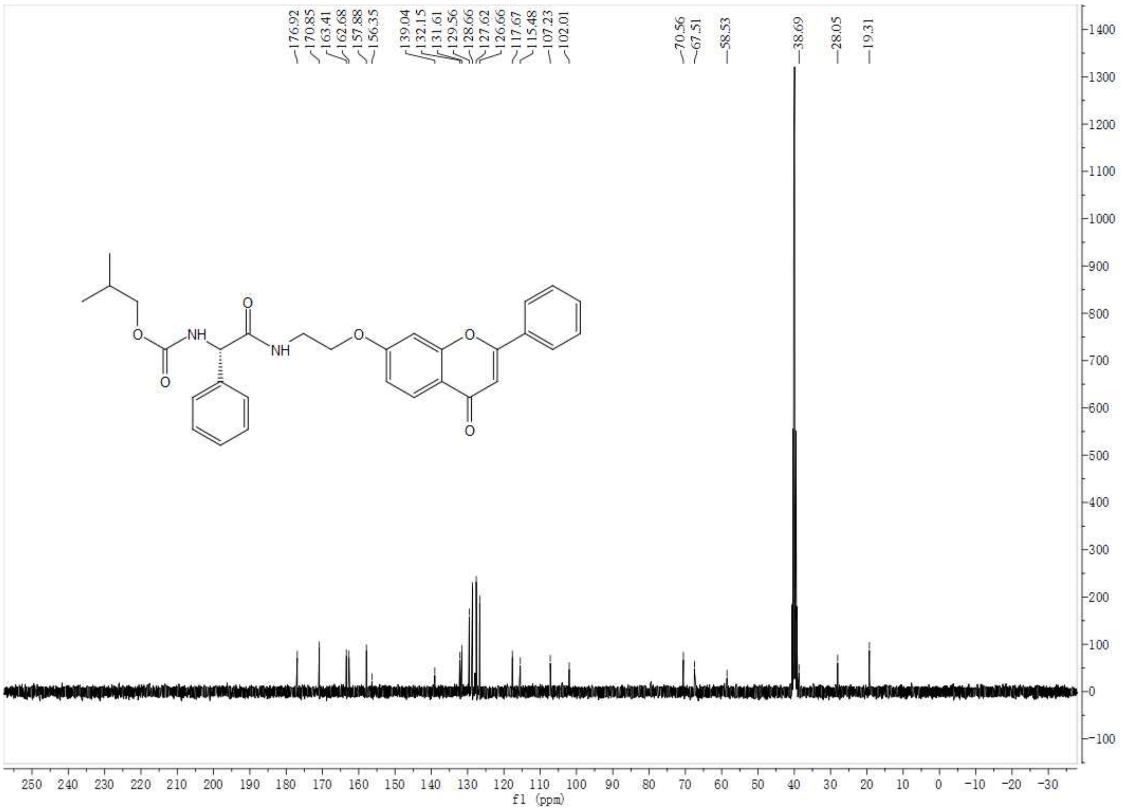

Figure S34. ^{13}C NMR spectrum of **4j**

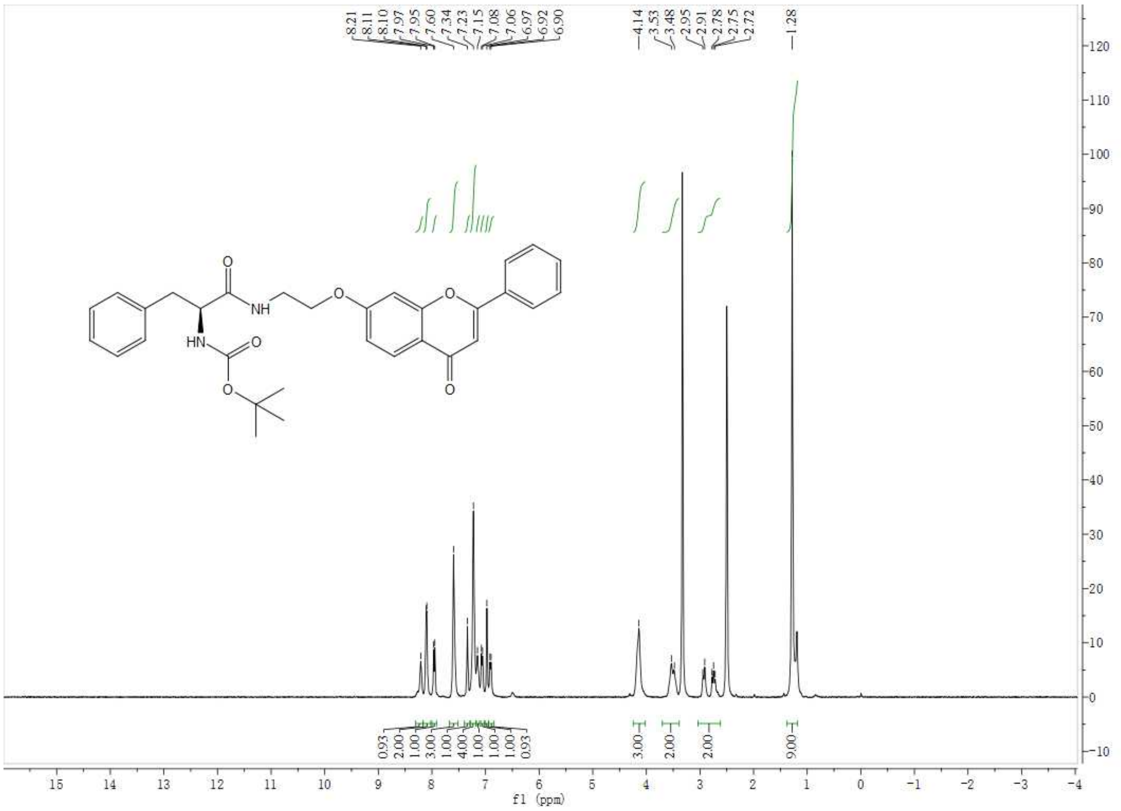

Figure S35. ^{1}H NMR spectrum of **4k**

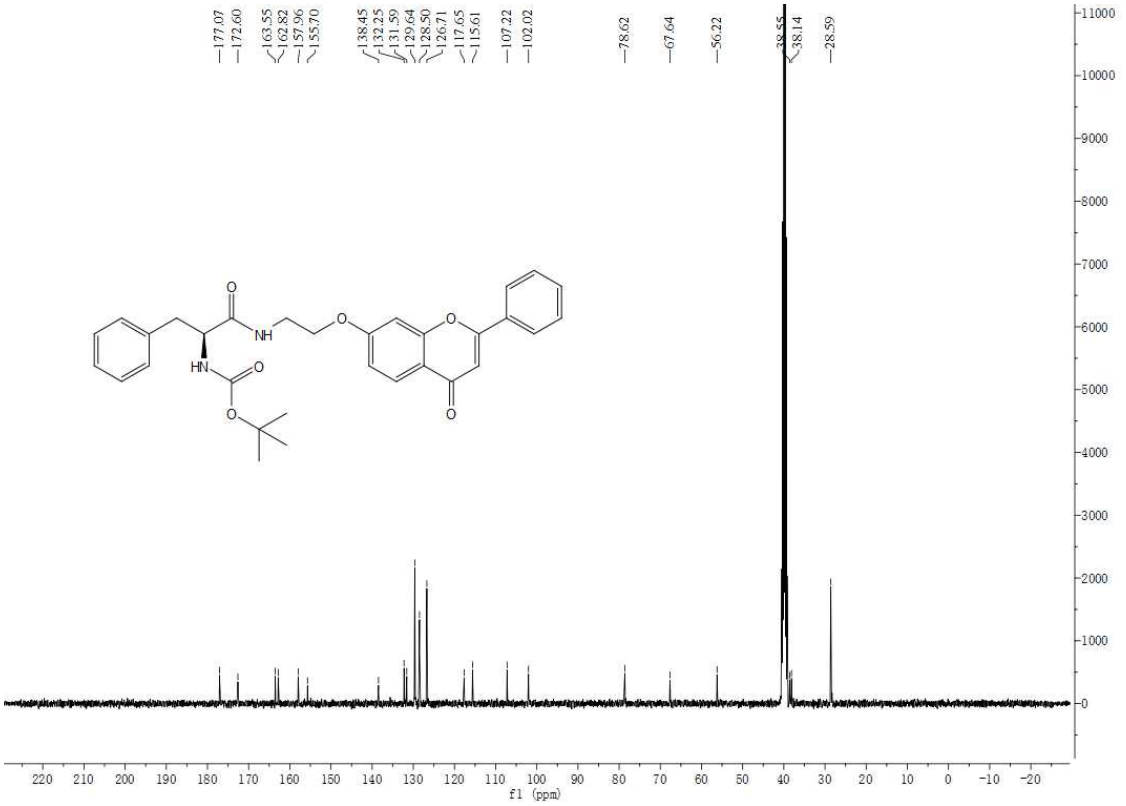

Figure S36. ^{13}C NMR spectrum of **4k**

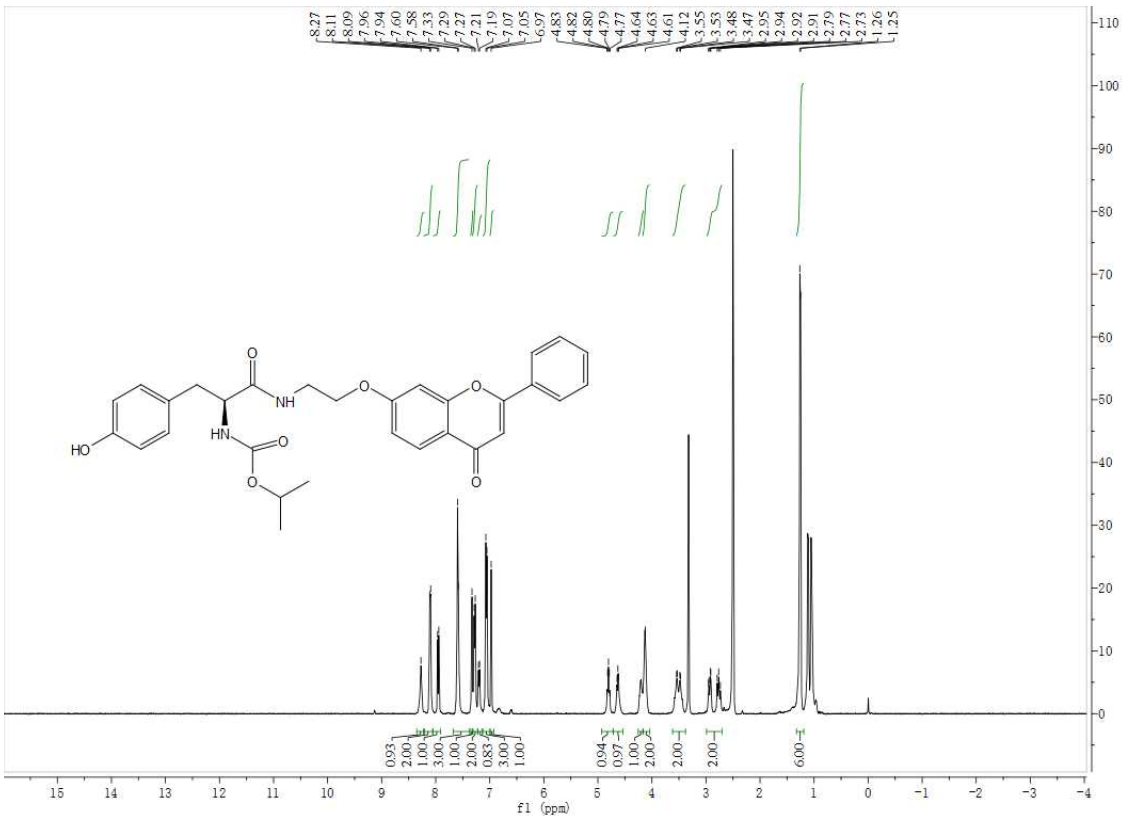

Figure S37. ^{1}H NMR spectrum of **4l**

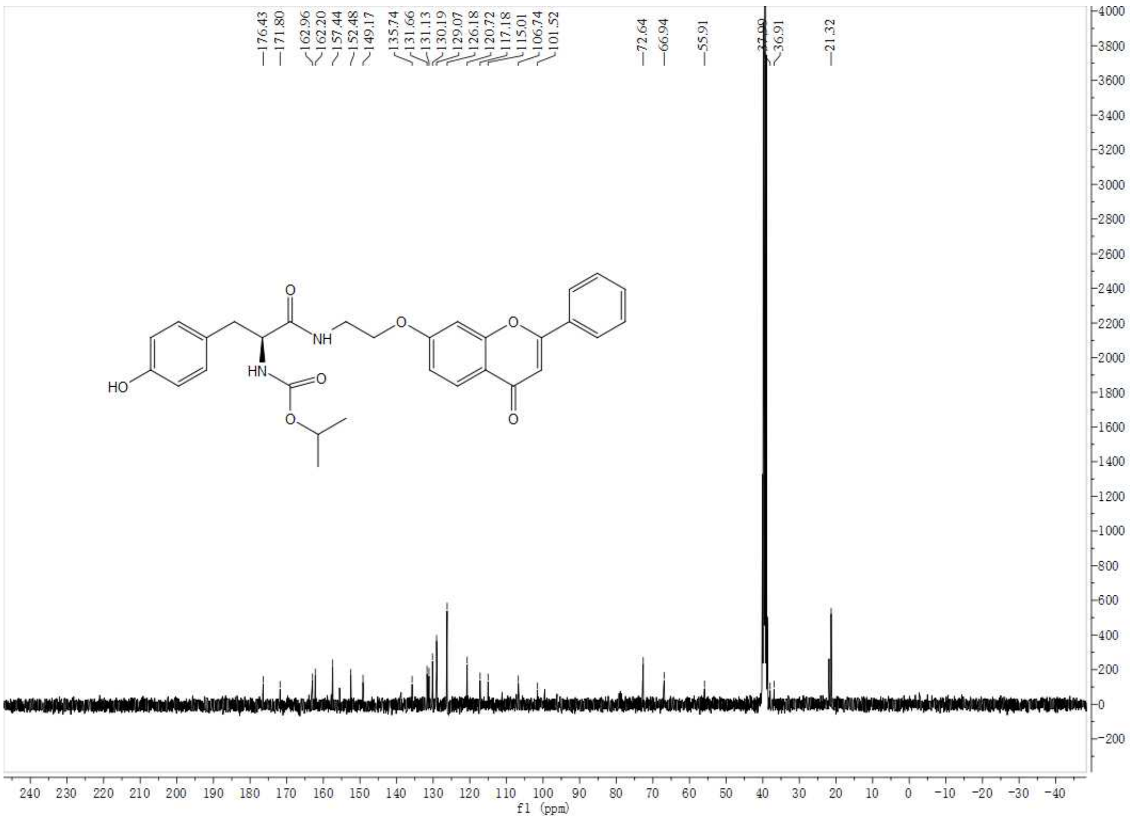

Figure S38. ^{13}C NMR spectrum of **4l**

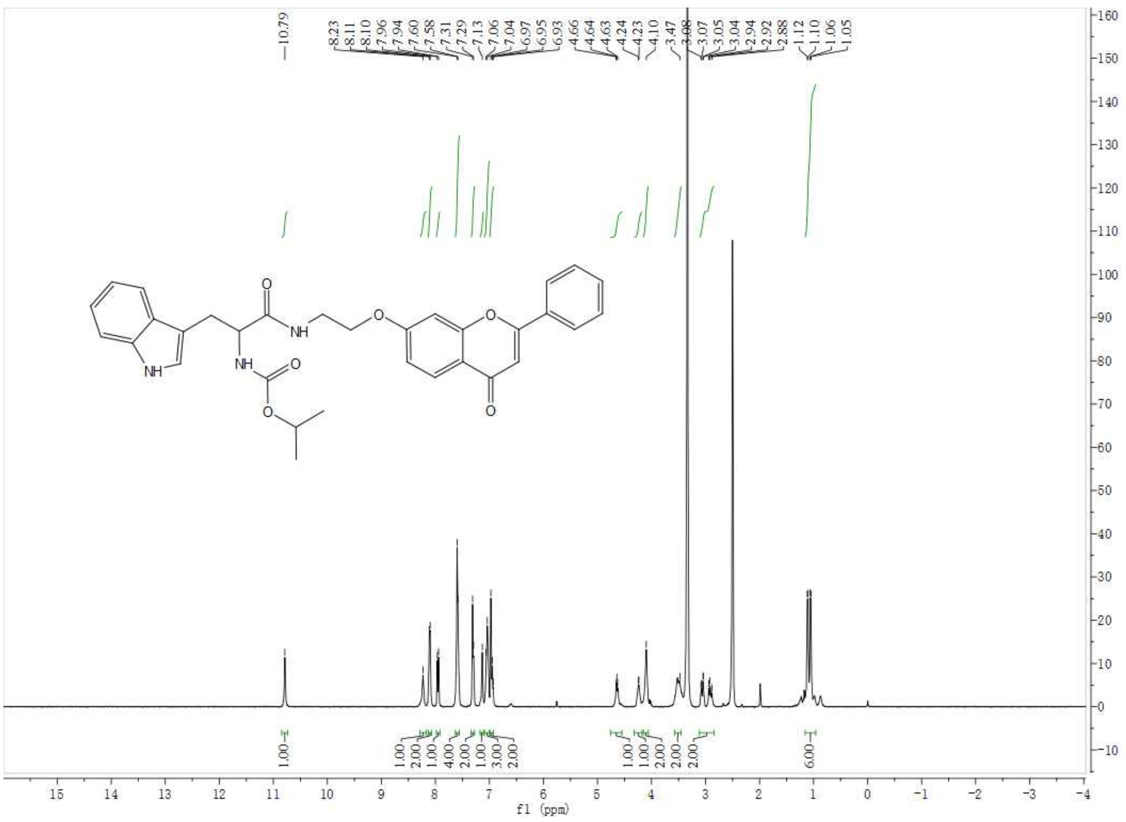

Figure S39. ^{1}H NMR spectrum of **4m**

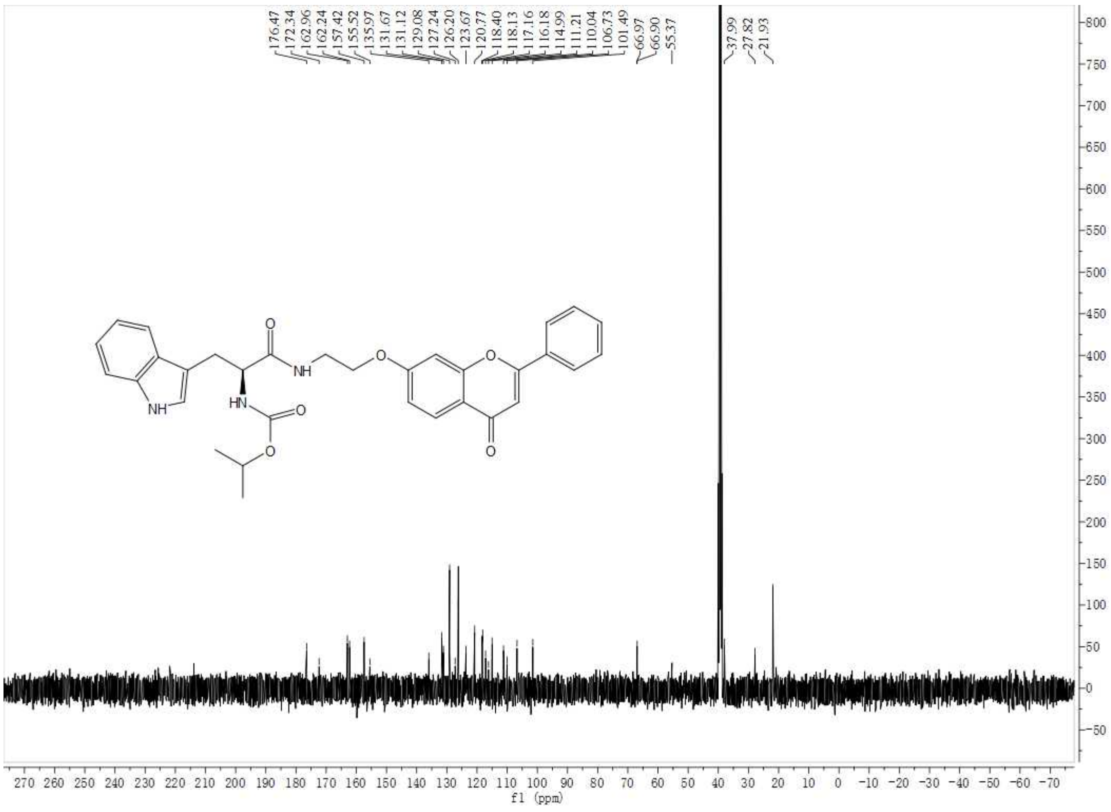

Figure S40. ^{13}C NMR spectrum of **4m**

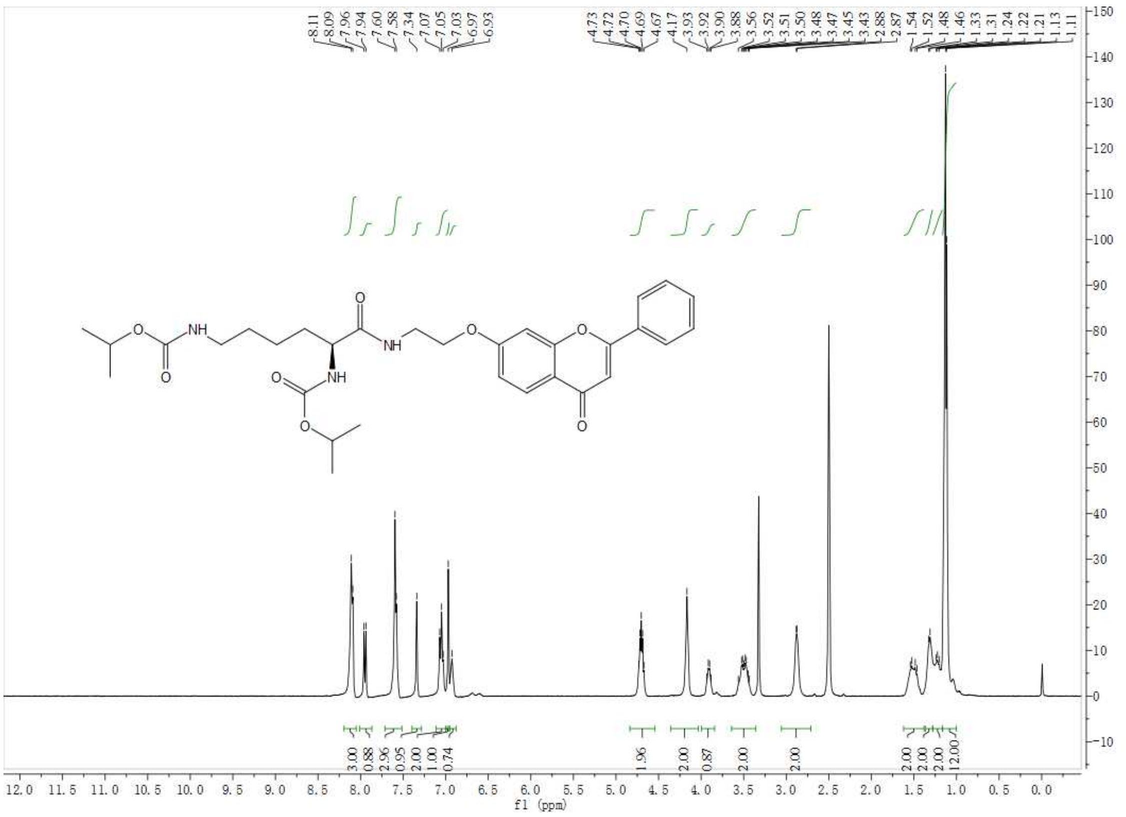

Figure S41. ^1^H NMR spectrum of **4n**

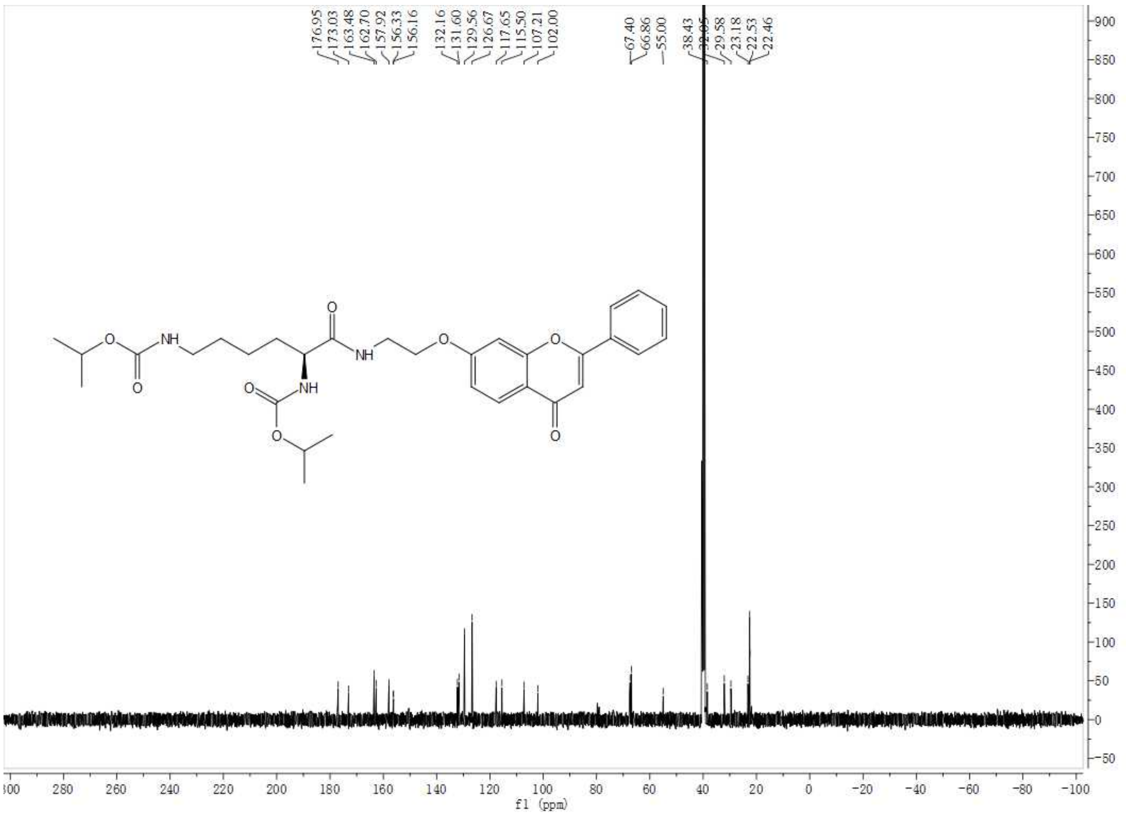

Figure S42. ^13^C NMR spectrum of **4n**

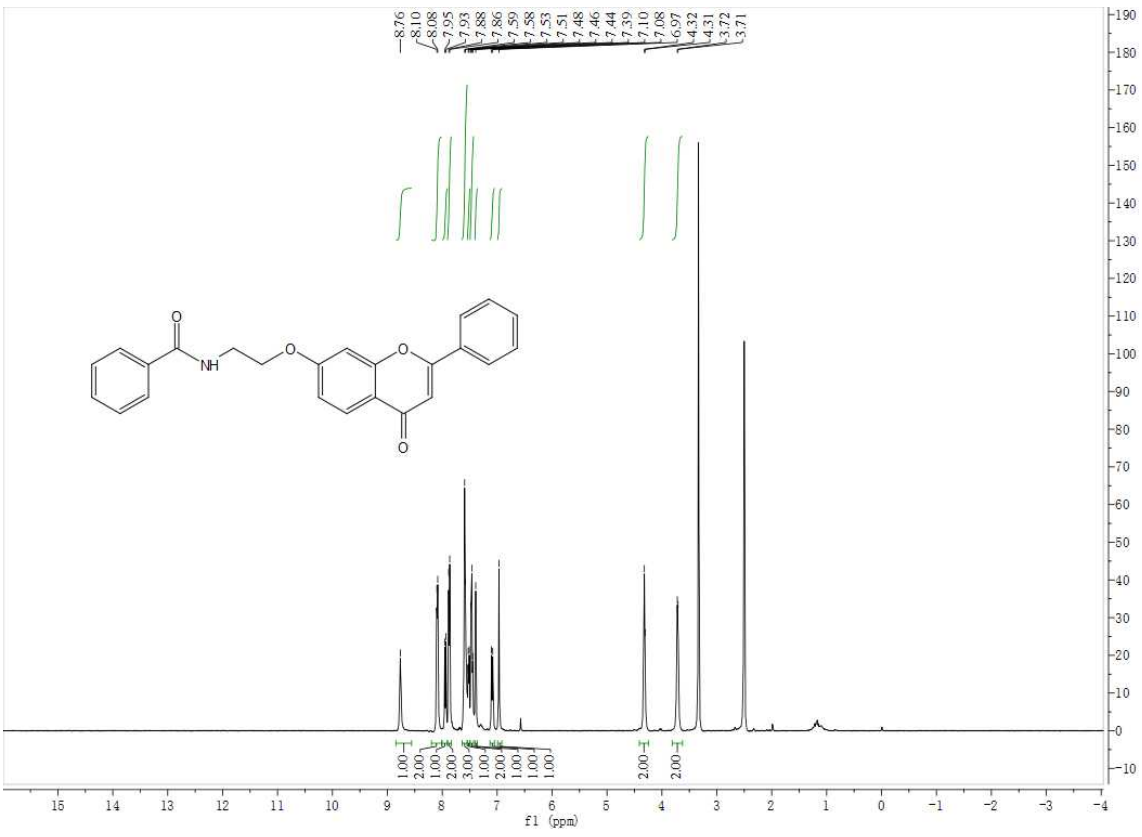

Figure S43. ¹H NMR spectrum of **5a**

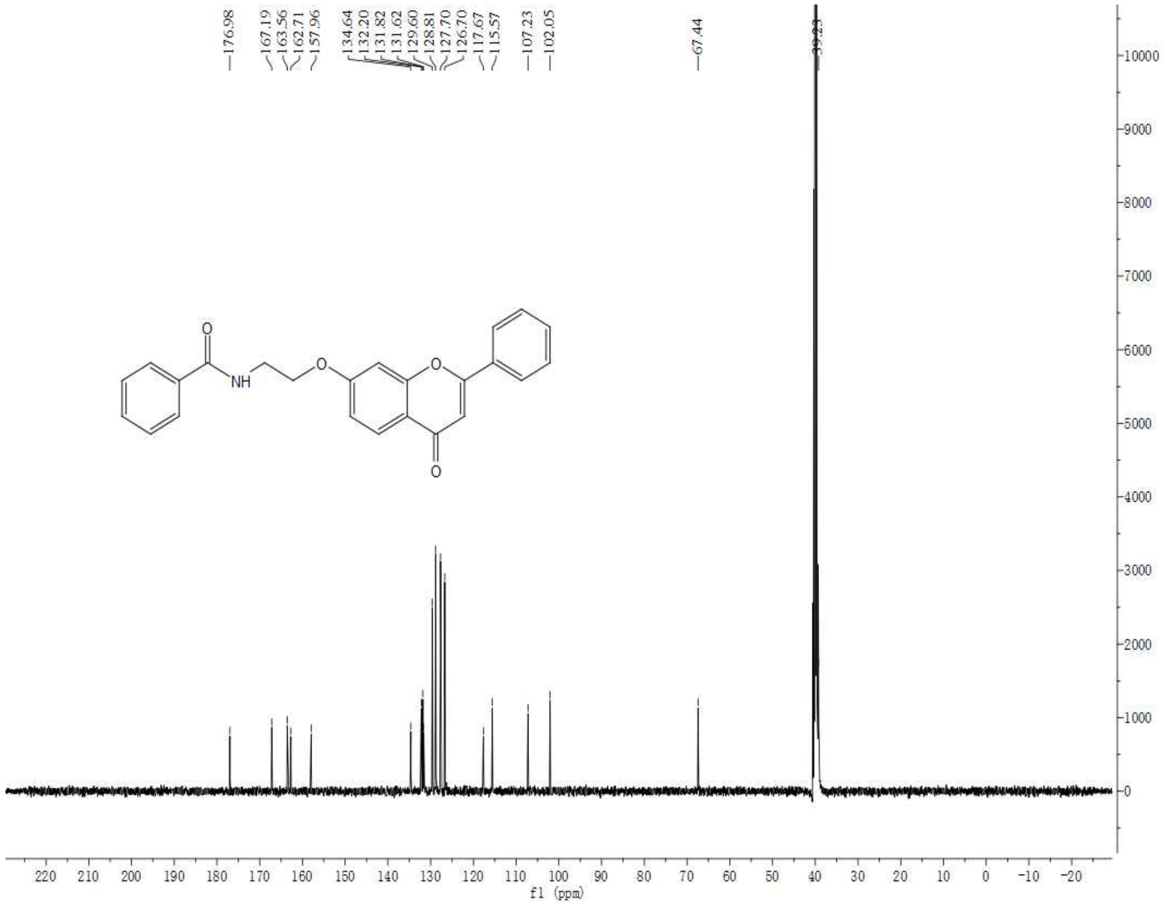

Figure S44. ^{13}C NMR spectrum of **5a**

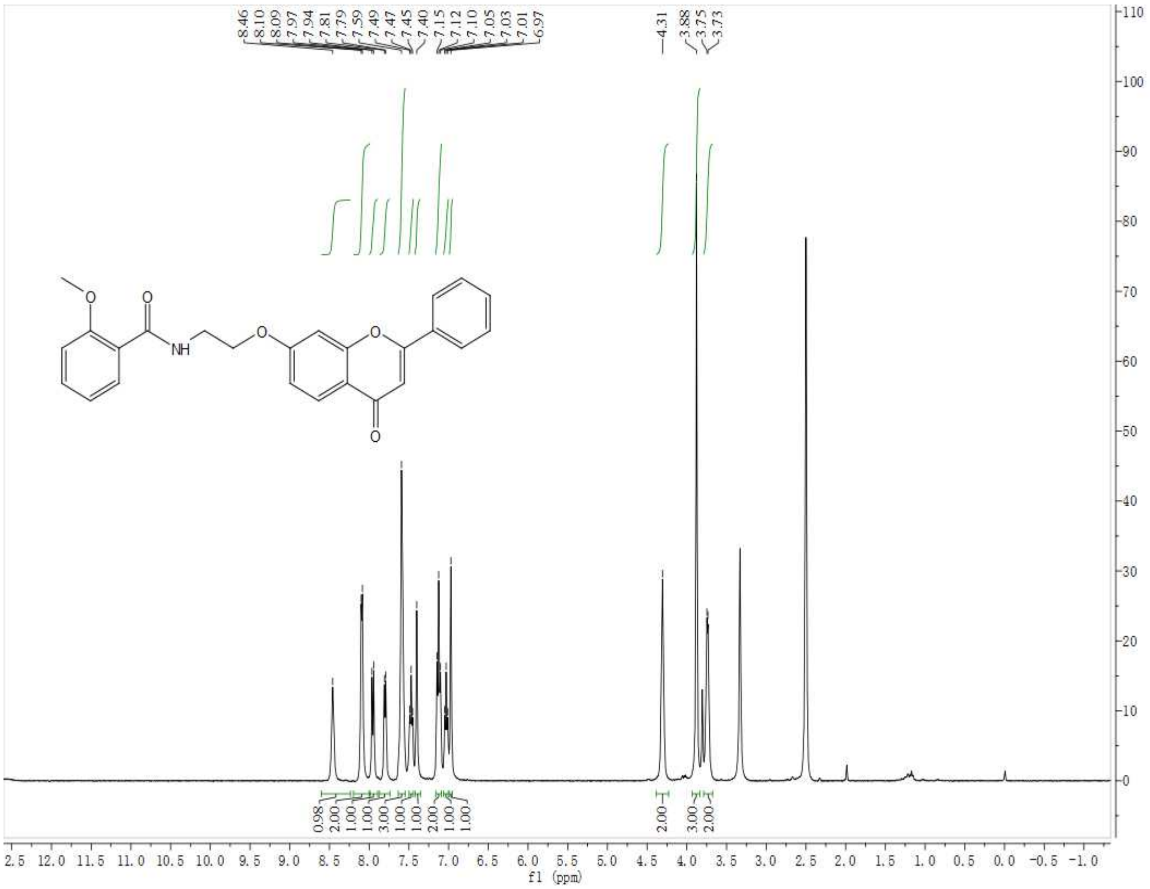

Figure S45. ^{1}H NMR spectrum of **5b**

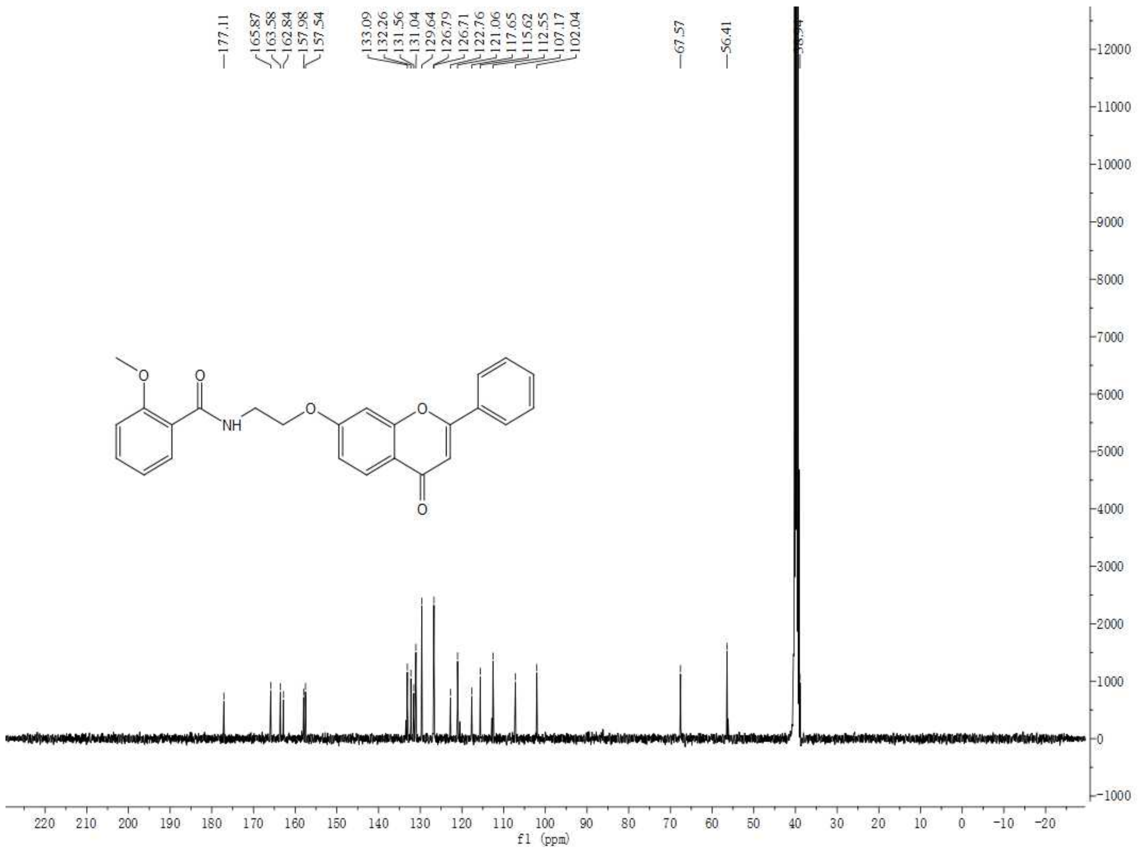

Figure S46. ^{13}C NMR spectrum of **5b**

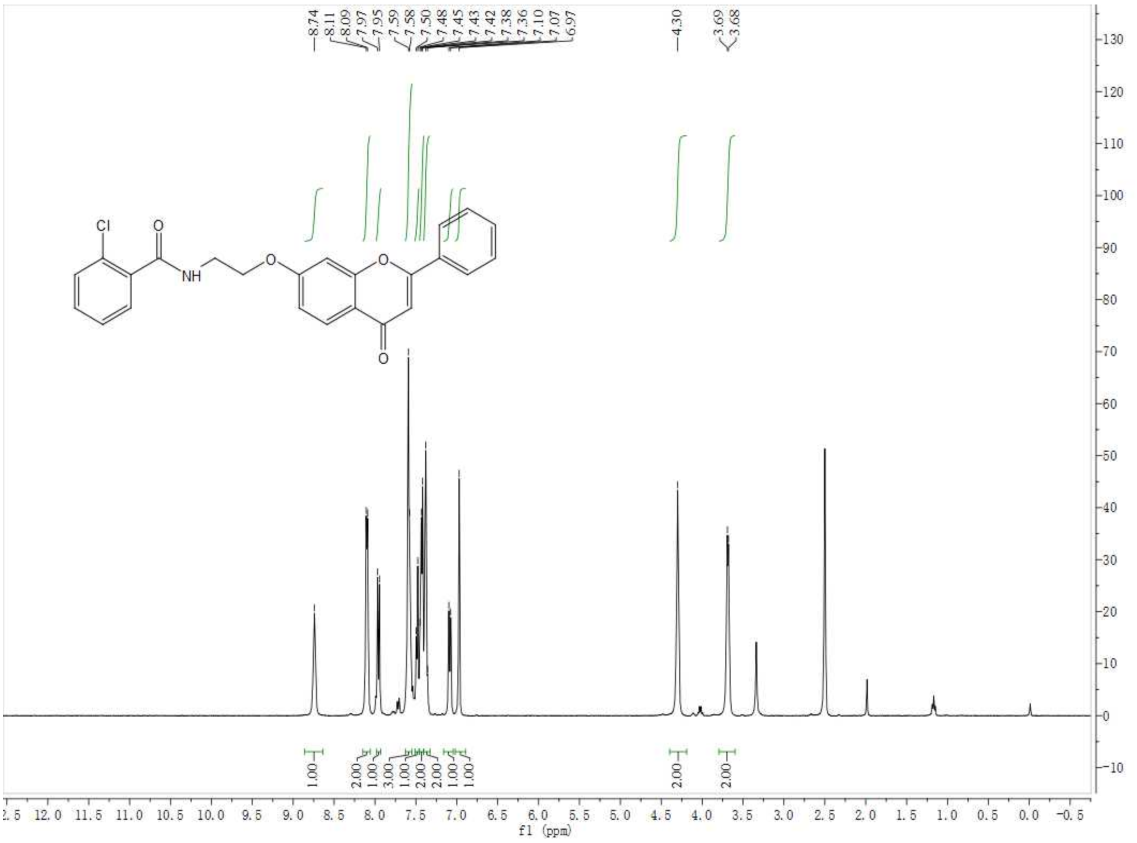

Figure S47. ^{1}H NMR spectrum of **5c**

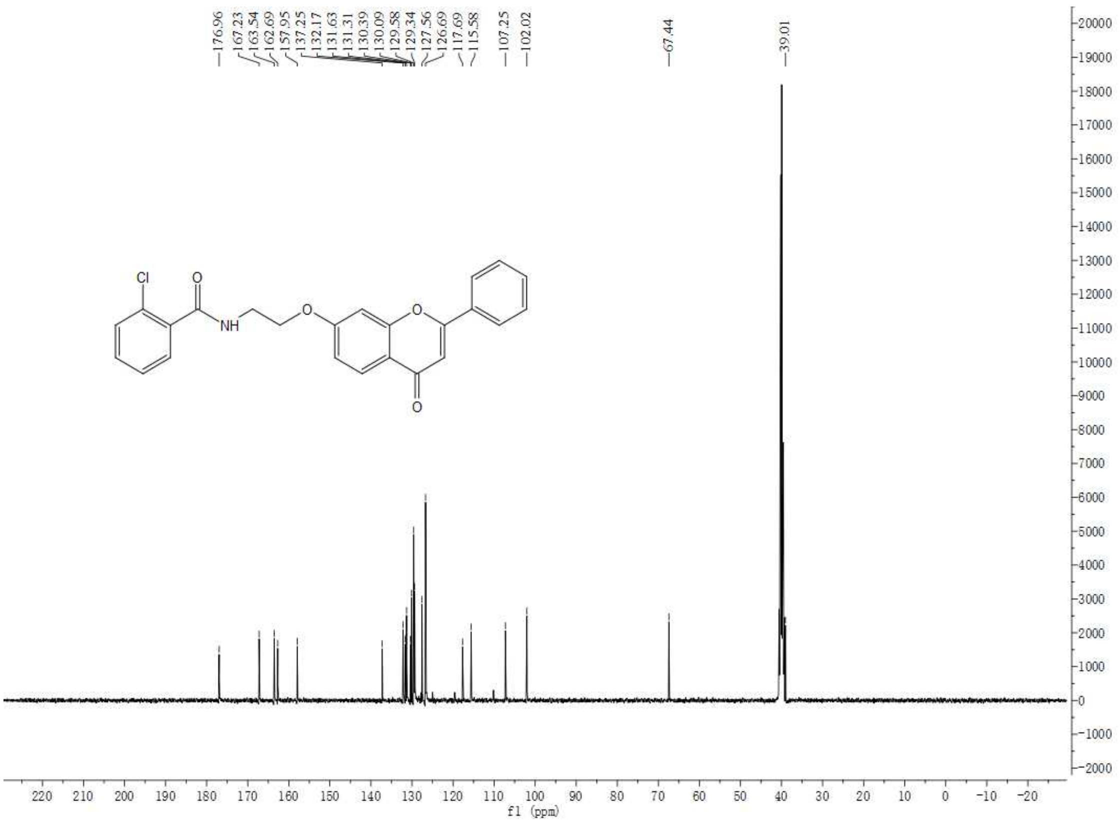

Figure S48. ^{13}C NMR spectrum of **5c**

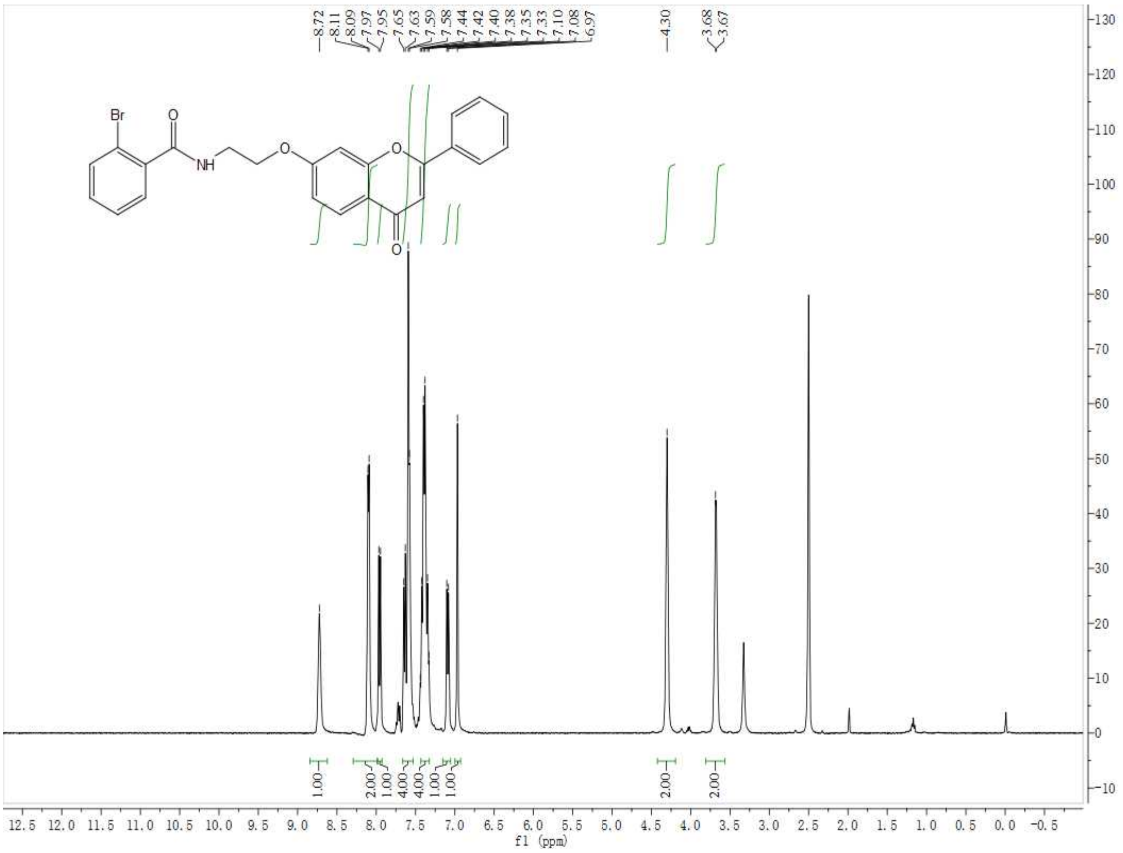

Figure S49. ^{1}H NMR spectrum of **5d**

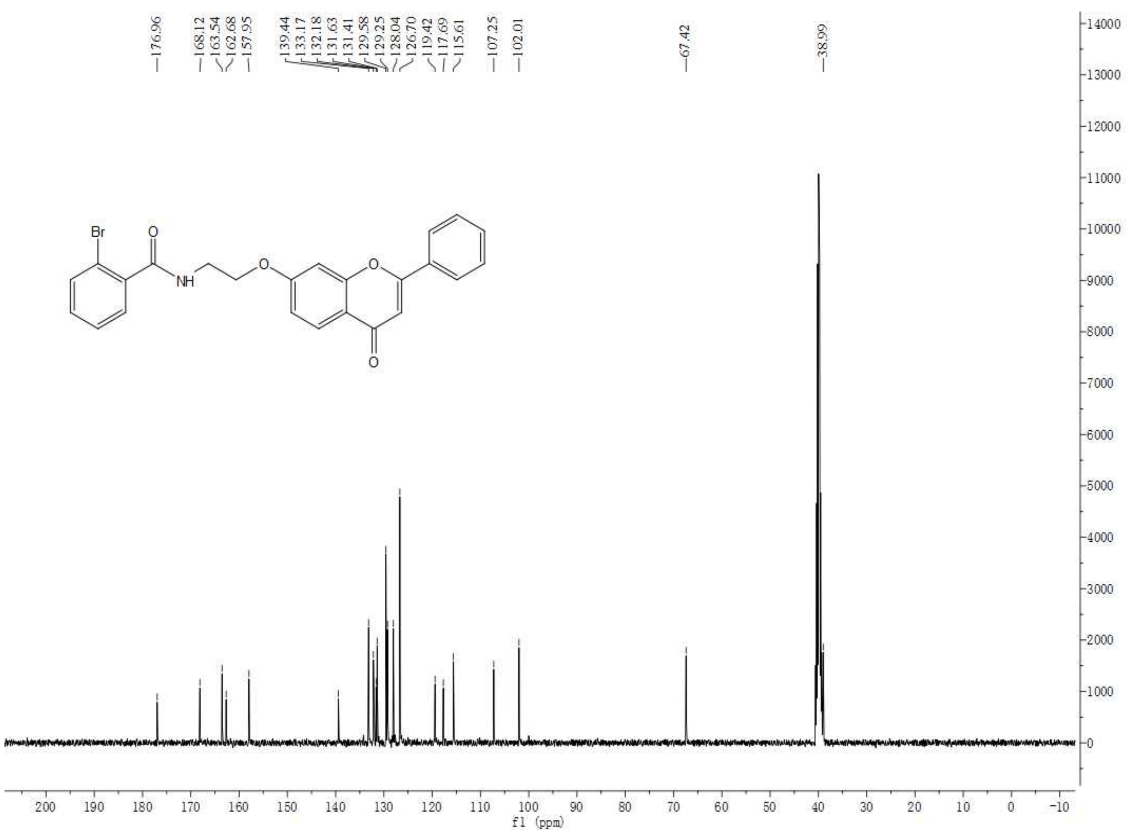

Figure S50. ^{13}C NMR spectrum of **5d**

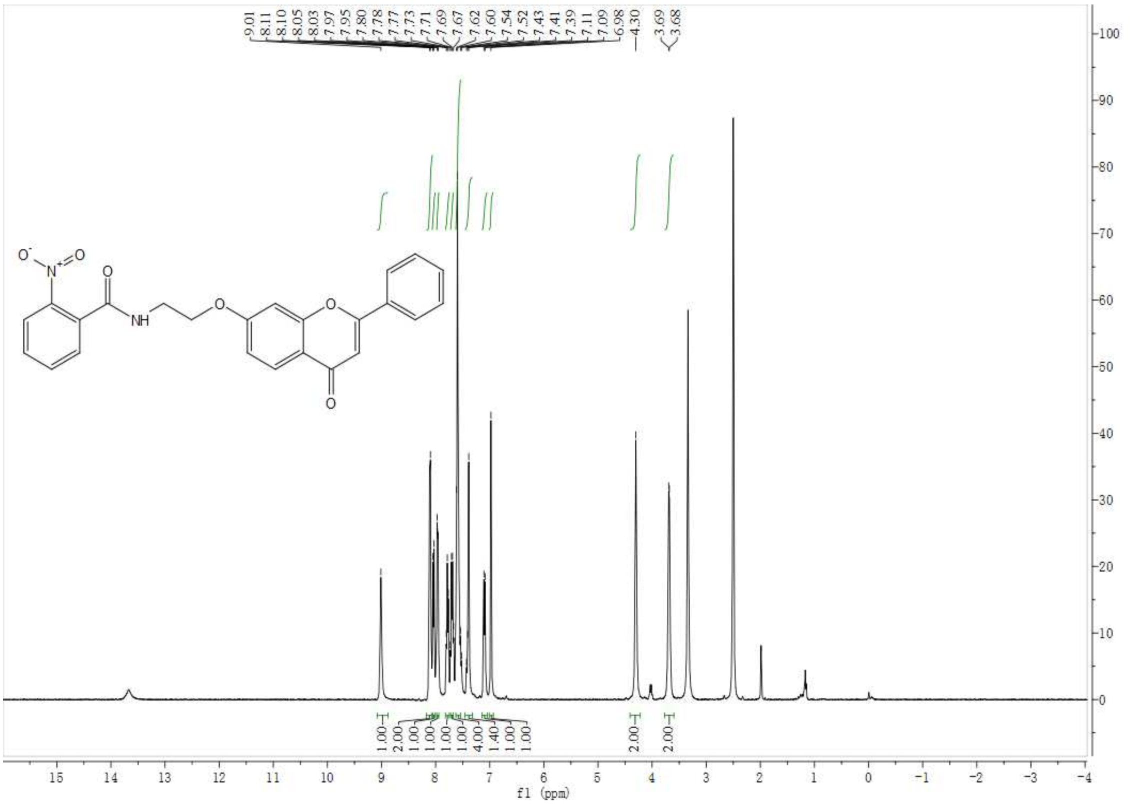

Figure S51. ^{1}H NMR spectrum of **5e**

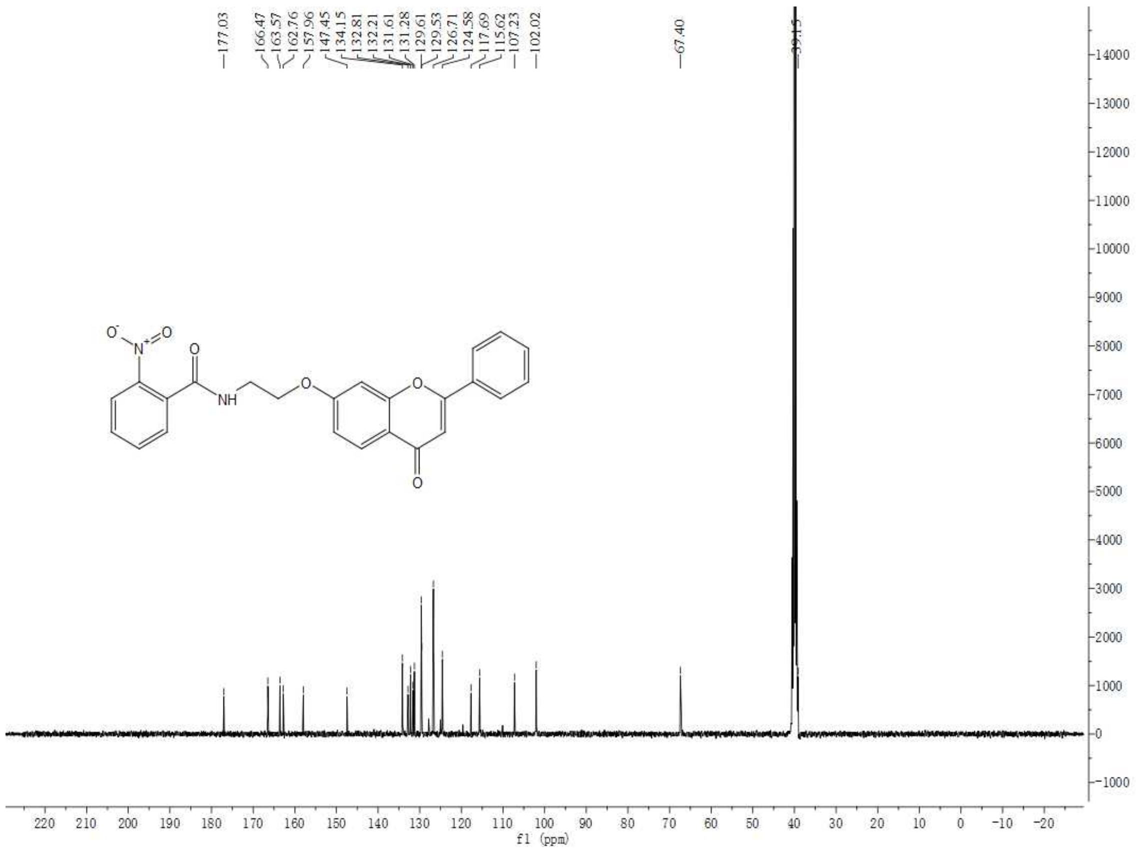

Figure S52. ^{13}C NMR spectrum of **5e**

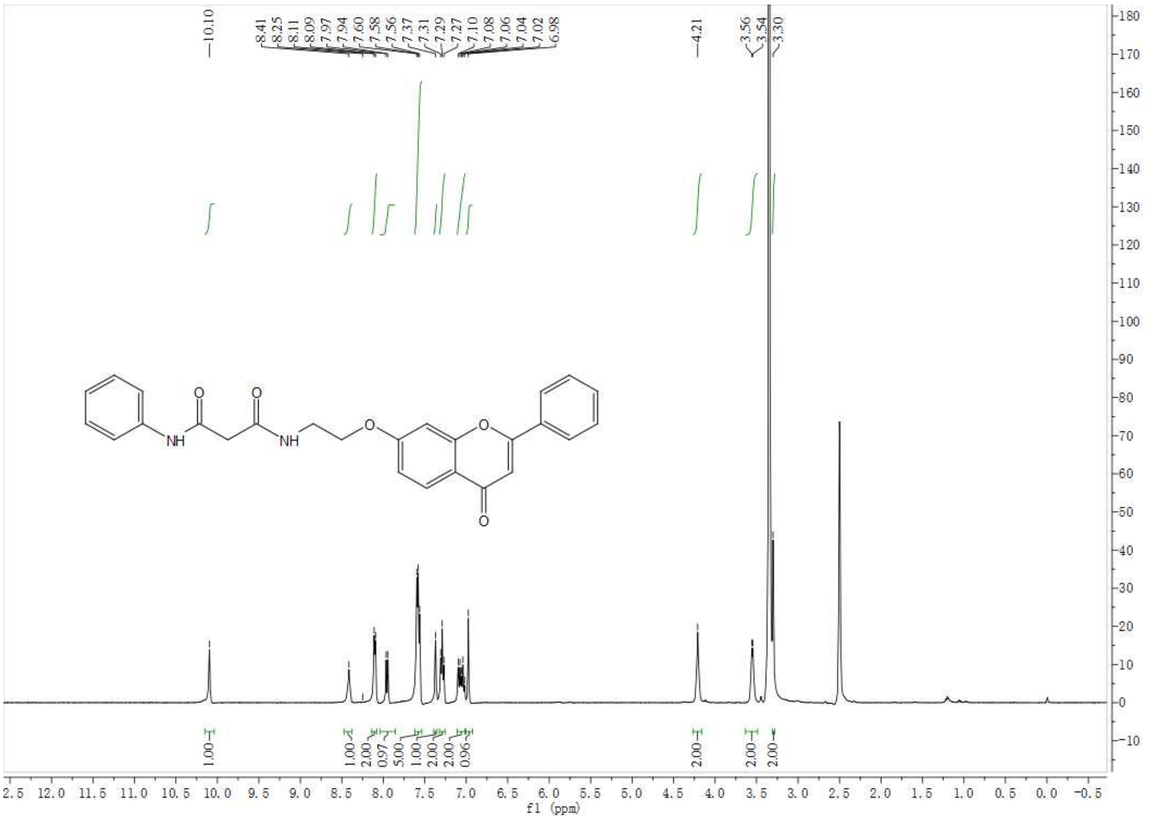

Figure S53. ^{1}H NMR spectrum of **6a**

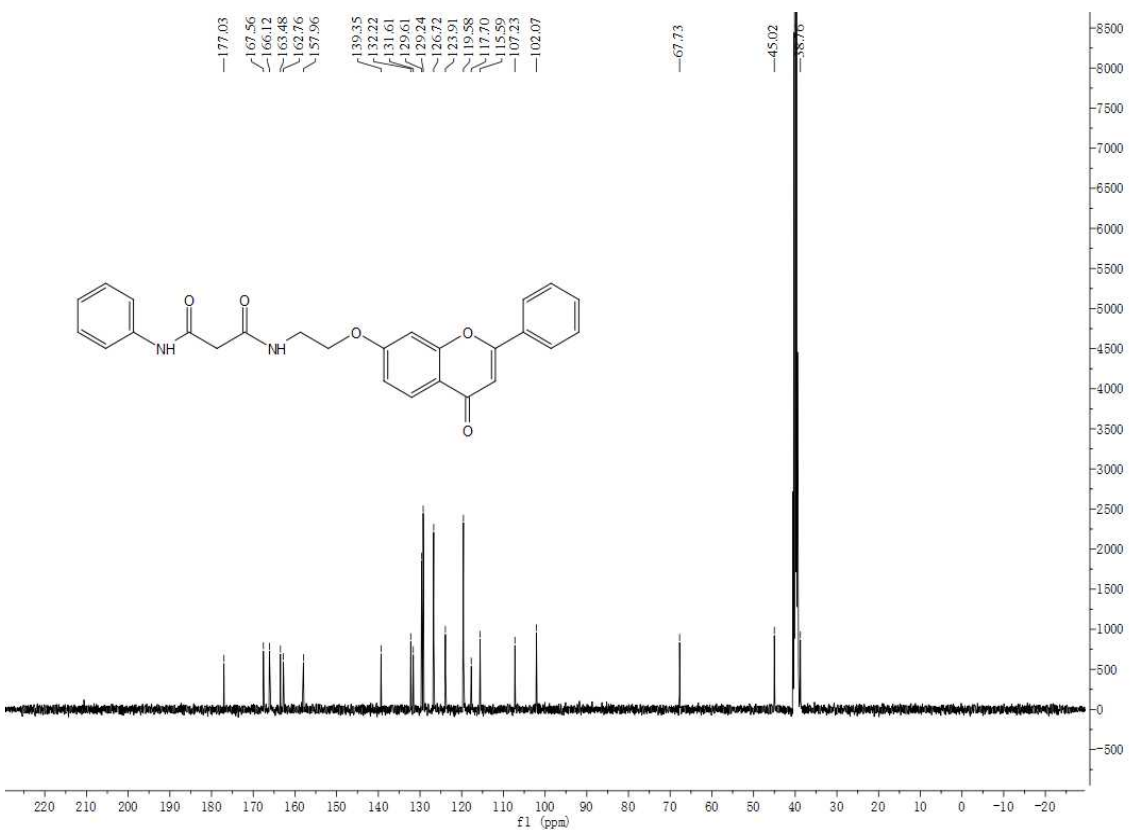

Figure S54. ^{13}C NMR spectrum of **6a**

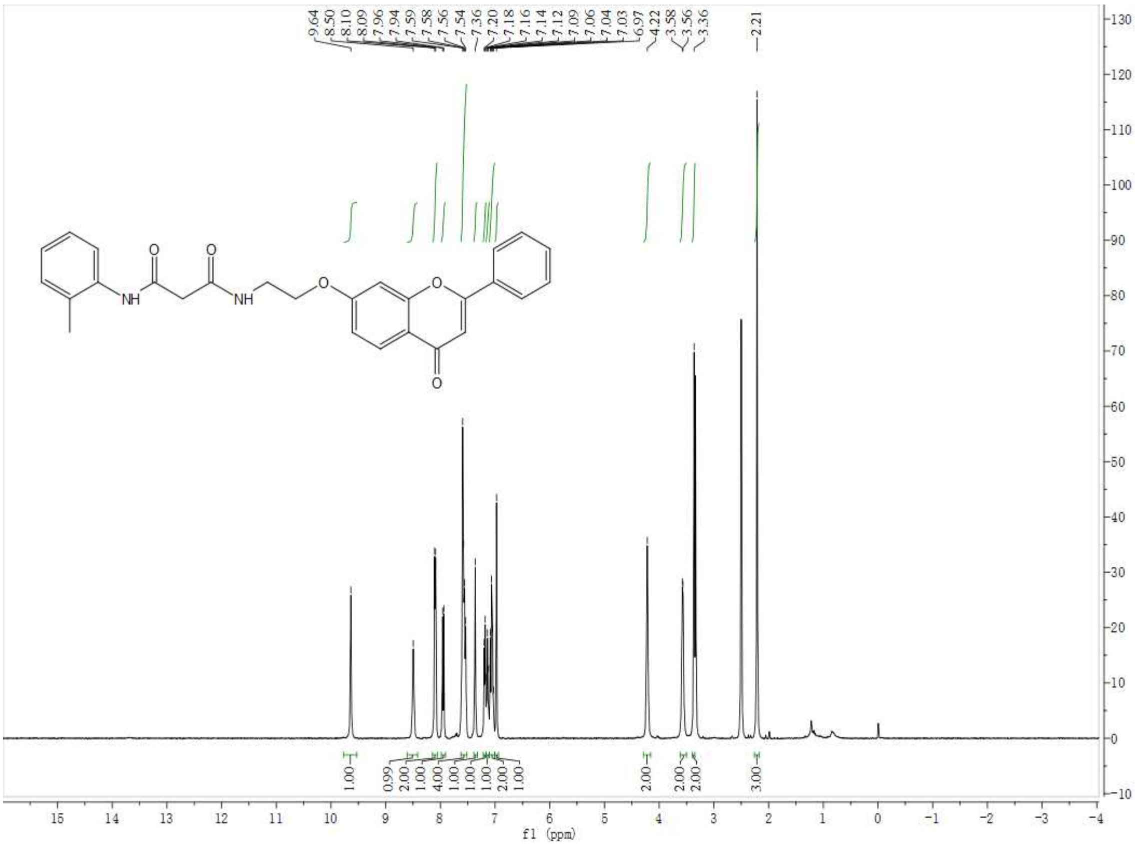

Figure S55. ^{1}H NMR spectrum of **6b**

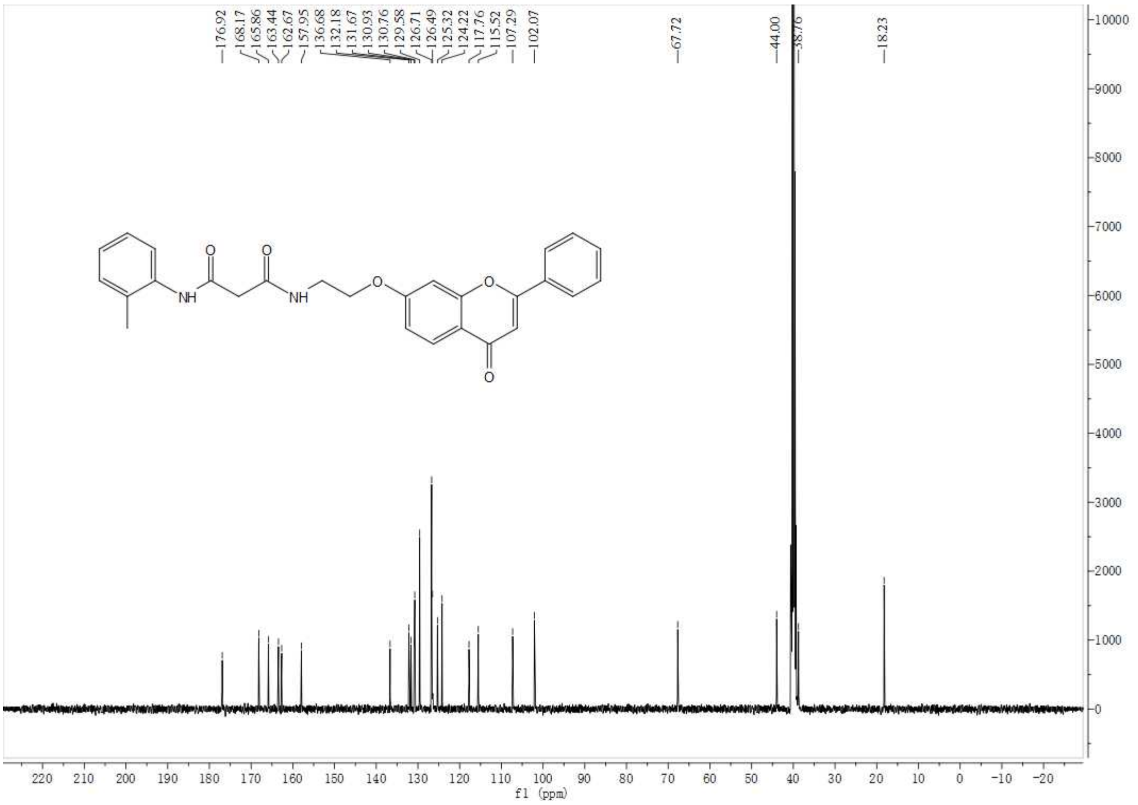

Figure S56. ^{13}C NMR spectrum of **6b**

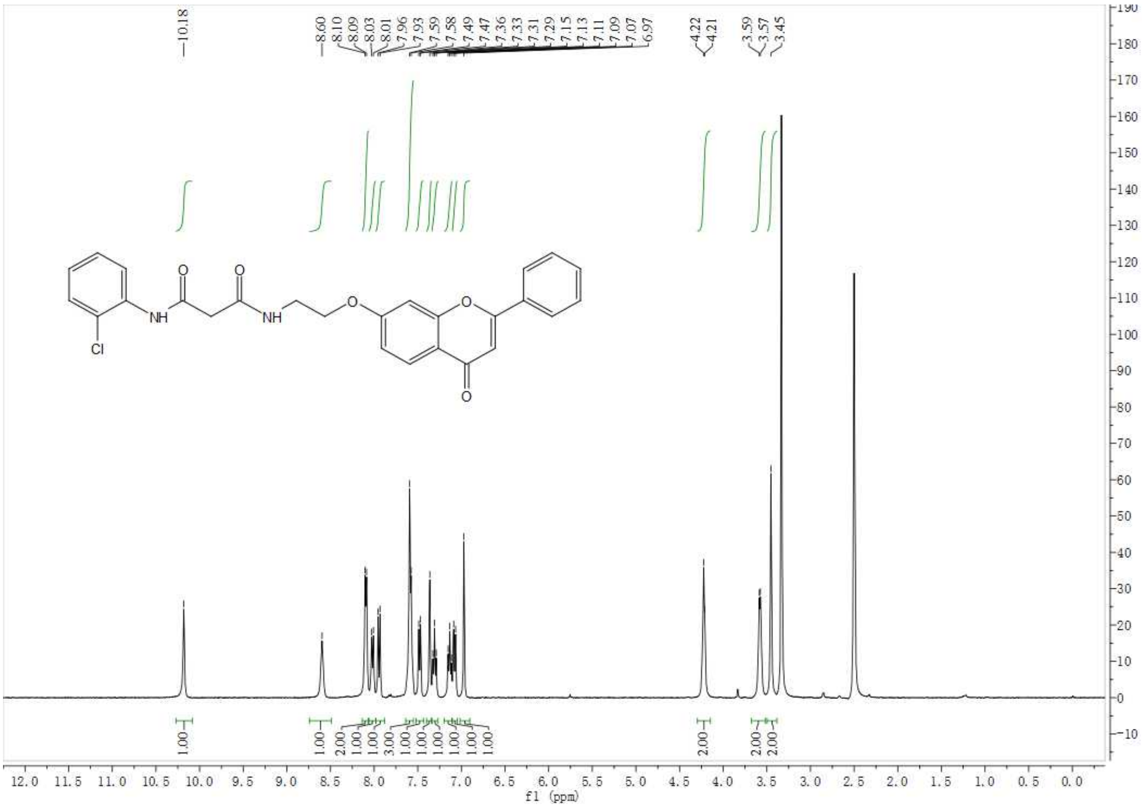

Figure S57. ^{1}H NMR spectrum of **6c**

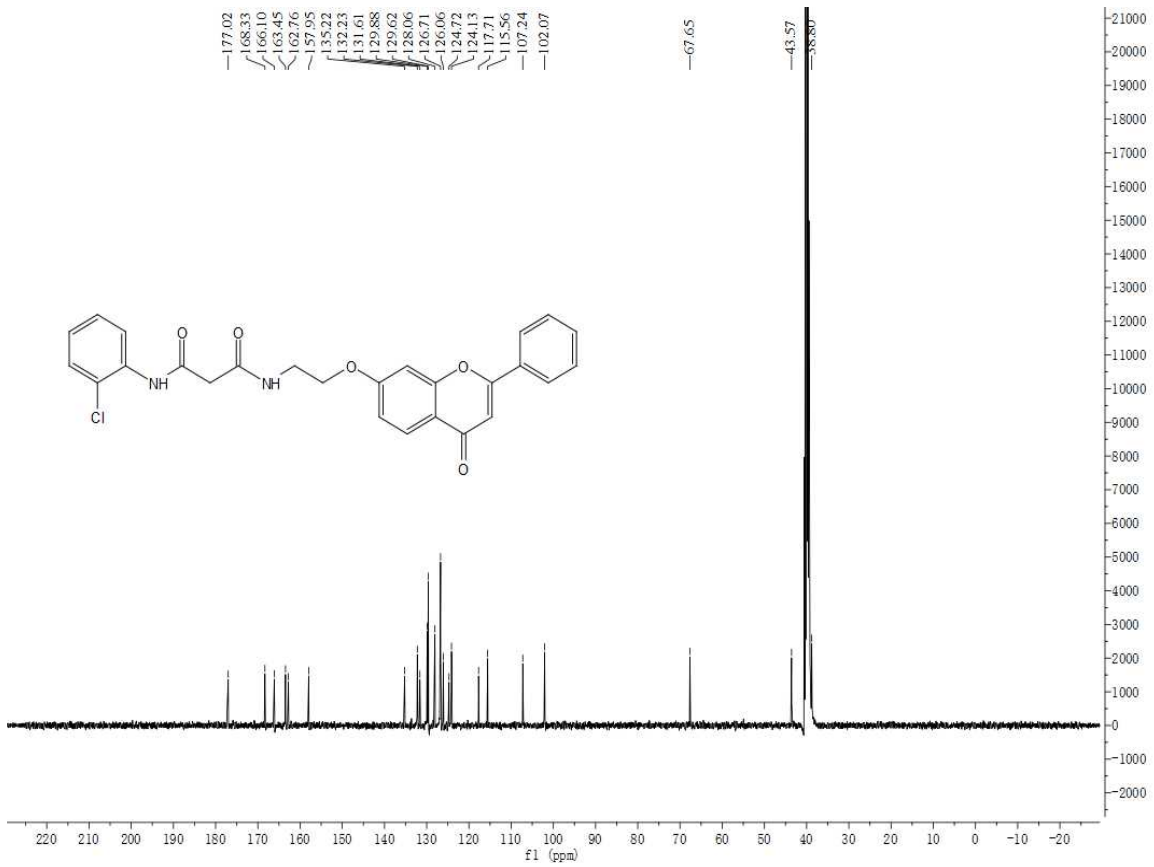

Figure S58. ^{13}C NMR spectrum of **6c**

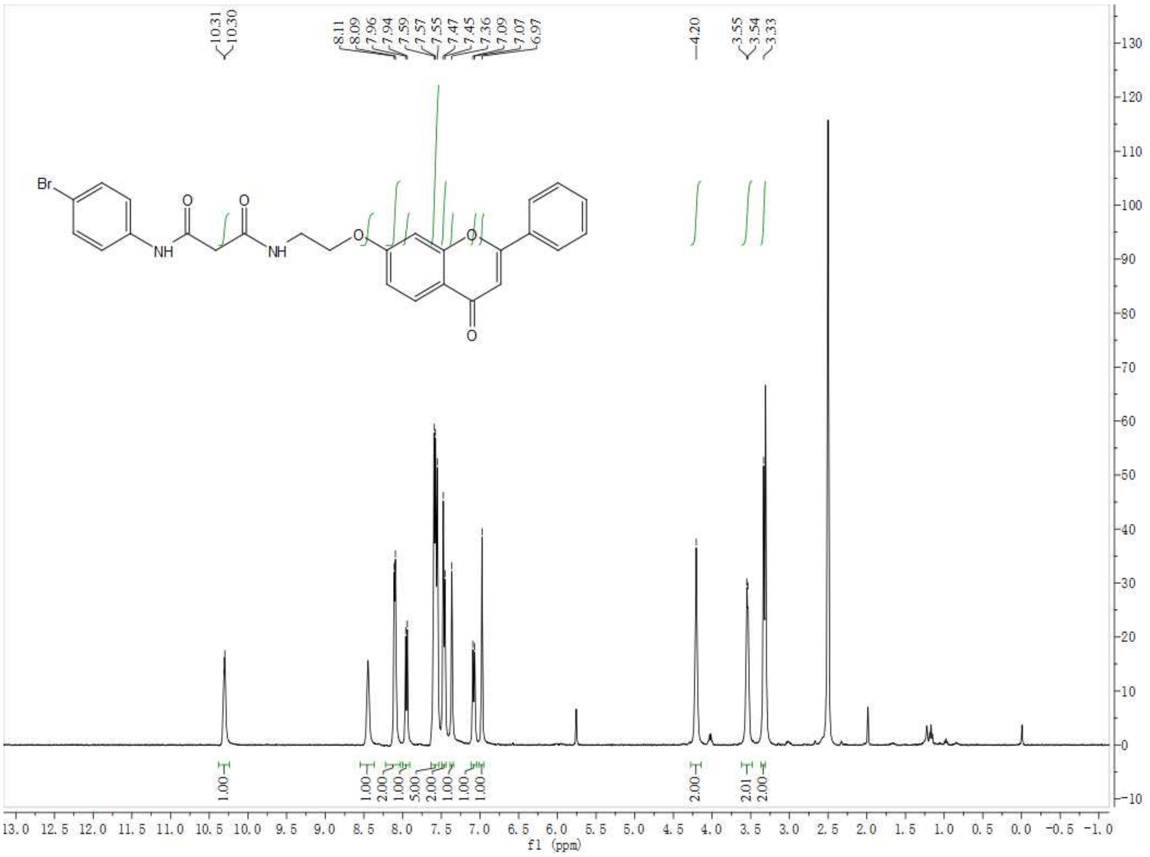

Figure S59. ^{1}H NMR spectrum of **6d**

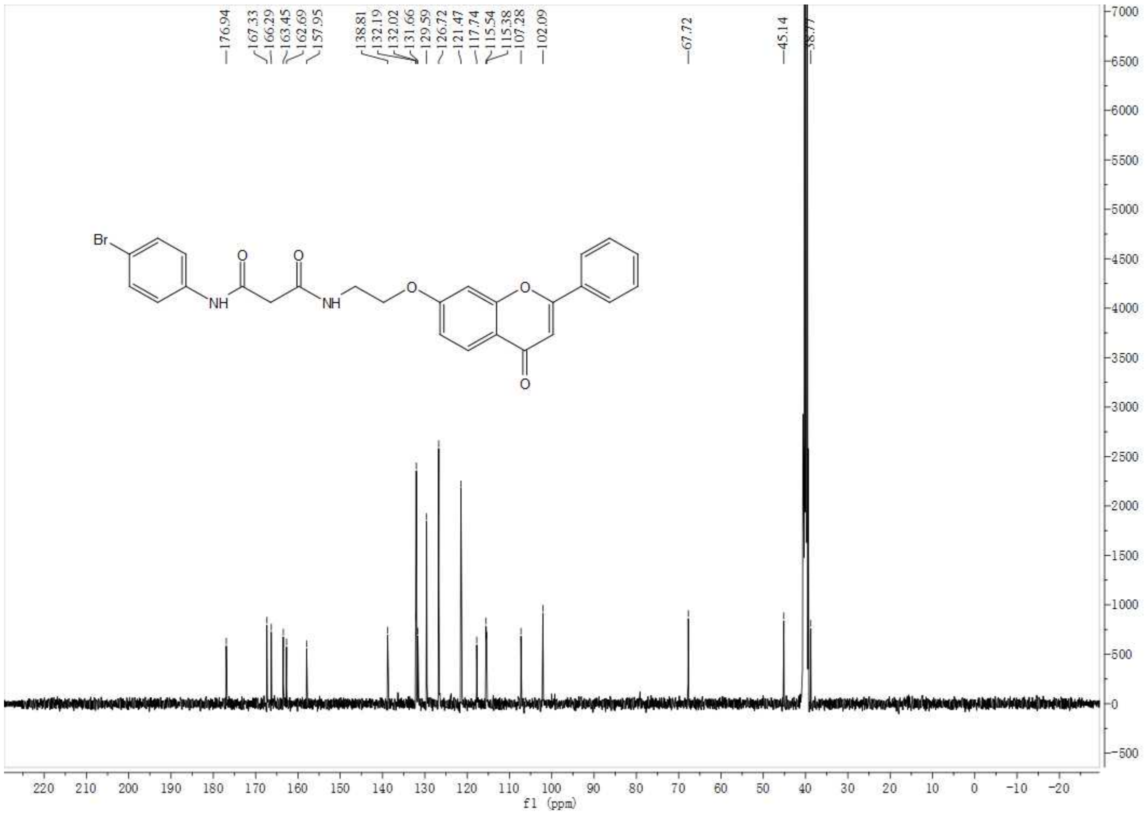

Figure S60. ^{13}C NMR spectrum of **6d**

Section S3: Molecule docking results of 1 and 2a with TMV CP.

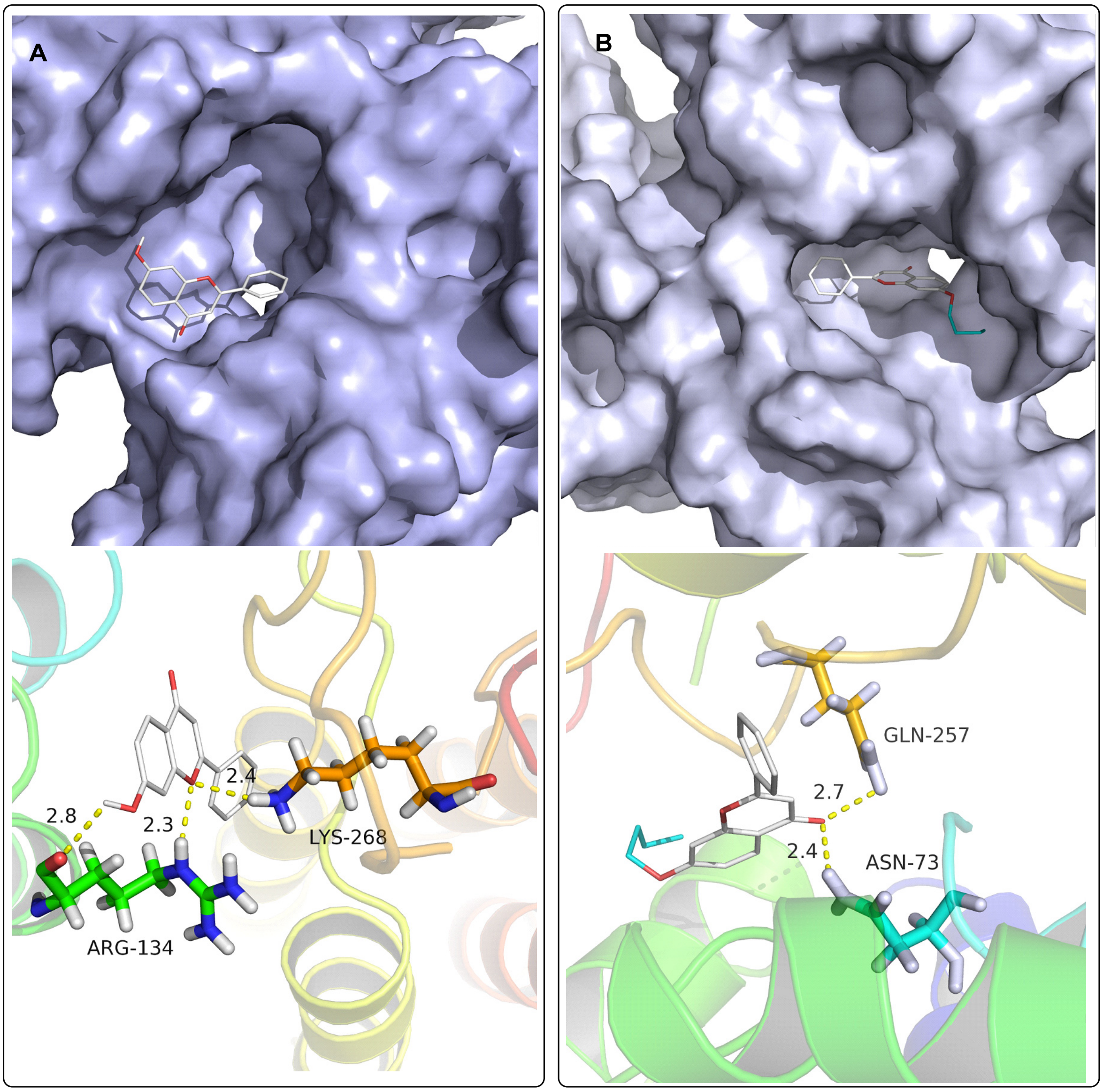

Figure S61. Molecule docking results of **1** (A) and **2a** (B) with TMV CP
